# Single-cell multiregion dissection of Alzheimer’s disease

**DOI:** 10.1038/s41586-024-07606-7

**Published:** 2024-07-24

**Authors:** Hansruedi Mathys, Carles A. Boix, Leyla Anne Akay, Ziting Xia, Jose Davila-Velderrain, Ayesha P. Ng, Xueqiao Jiang, Ghada Abdelhady, Kyriaki Galani, Julio Mantero, Neil Band, Benjamin T. James, Sudhagar Babu, Fabiola Galiana-Melendez, Kate Louderback, Dmitry Prokopenko, Rudolph E. Tanzi, David A. Bennett, Li-Huei Tsai, Manolis Kellis

**Affiliations:** 1 Picower Institute for Learning and Memory, MIT, Cambridge, MA USA; 2 Department of Brain and Cognitive Sciences, MIT, Cambridge, MA USA; 3 University of Pittsburgh Brain Institute, University of Pittsburgh School of Medicine, Pittsburgh, PA USA; 4 Department of Neurobiology, University of Pittsburgh School of Medicine, Pittsburgh, PA USA; 5 Computer Science and Artificial Intelligence Laboratory, MIT, Cambridge, MA USA; 6 Broad Institute of MIT and Harvard, Cambridge, MA USA; 7 Computational and Systems Biology Program, MIT, Cambridge, MA USA; 8 Harvard-MIT Health Sciences and Technology Program, MIT, Cambridge, MA USA; 9 Human Technopole, Milan, Italy; 10 Genetics and Aging Research Unit, McCance Center for Brain Health, Department of Neurology, Massachusetts General Hospital and Harvard Medical School, Boston, MA USA; 11 Rush Alzheimer’s Disease Center, Chicago, IL USA; 12 Present Address: Department of Computer Science, Stanford University, Stanford, CA USA

**Keywords:** Alzheimer's disease, Molecular neuroscience, Gene expression

## Abstract

Alzheimer’s disease is the leading cause of dementia worldwide, but the cellular pathways that underlie its pathological progression across brain regions remain poorly understood^1–3^. Here we report a single-cell transcriptomic atlas of six different brain regions in the aged human brain, covering 1.3 million cells from 283 post-mortem human brain samples across 48 individuals with and without Alzheimer’s disease. We identify 76 cell types, including region-specific subtypes of astrocytes and excitatory neurons and an inhibitory interneuron population unique to the thalamus and distinct from canonical inhibitory subclasses. We identify vulnerable populations of excitatory and inhibitory neurons that are depleted in specific brain regions in Alzheimer’s disease, and provide evidence that the Reelin signalling pathway is involved in modulating the vulnerability of these neurons. We develop a scalable method for discovering gene modules, which we use to identify cell-type-specific and region-specific modules that are altered in Alzheimer’s disease and to annotate transcriptomic differences associated with diverse pathological variables. We identify an astrocyte program that is associated with cognitive resilience to Alzheimer’s disease pathology, tying choline metabolism and polyamine biosynthesis in astrocytes to preserved cognitive function late in life. Together, our study develops a regional atlas of the ageing human brain and provides insights into cellular vulnerability, response and resilience to Alzheimer’s disease pathology.

## Abstract (web summary)

A regional atlas of the ageing human brain—spanning six distinct anatomical regions from individuals with and without Alzheimer’s dementia—provides insights into cellular vulnerability, response and resilience to Alzheimer’s disease pathology

## Main

Alzheimer’s disease (AD) is characterized by pathological protein aggregation in a stereotyped pattern across multiple brain regions^1,4^. Post-mortem diagnosis of AD is staged by the severity and distribution of these pathological hallmarks: extracellular amyloid-β deposits and intracellular neurofibrillary tangles (NFTs) in neurons. Tangles are first seen in the entorhinal cortex (EC) (Braak stages I–II), then the hippocampus and thalamus (Braak stages III–IV) and finally the neocortex (Braak stages V–VI), a sequence that is typically synchronous with cognitive decline from mild cognitive impairment to severe dementia^1,2,4–7^. Understanding the cellular architecture of affected brain regions has important implications for early and region-specific therapeutic interventions and may shed light on the molecular mechanisms underlying the regional progression of pathology. Although some brain regions relevant to AD have been studied individually at scale or jointly in samples from a few individuals^8–16^, a comprehensive molecular characterization of region-specific differences in AD is currently lacking and could capture differences in regional molecular architecture^17–24^ and region-specific neuronal and glial subtype alterations in AD and in cognitive resilience to AD pathology^3,25–27^.

Here we present a transcriptomic atlas of the human brain spanning six distinct anatomical regions from persons with and without Alzheimer’s dementia as a basis for understanding disease-associated differences. We profile the transcriptomes of over 1.3 million nuclei from the EC, hippocampus (HC), anterior thalamus (TH), angular gyrus (AG), midtemporal cortex (MT) and prefrontal cortex (PFC) from 48 individuals, 26 of whom have a pathologic diagnosis of AD. We annotate region-specific neuronal and glial subtype diversity, present an online resource for navigating this atlas (http://compbio.mit.edu/ad_multiregion) and provide mechanistic insights into cellular vulnerability, response and resilience to AD.

## A multiregion atlas of AD

**Fig. 1:**
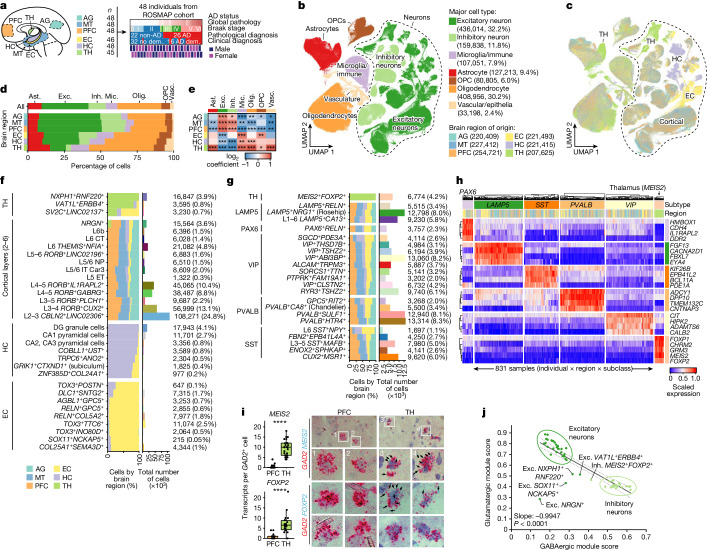
snRNA-seq analysis of six distinct regions of the aged human brain. **a**, snRNA-seq profiling summary, covering 283 samples across 6 brain regions from 48 participants from ROSMAP, showing global pathology, Braak stage and pathological (26 AD and 22 non-AD) or clinical diagnosis of AD (16 AD dementia (dem.) and 32 no dementia). **b**,**c**, Joint uniform manifold approximation and projection (UMAP), coloured by major cell type (**b**) and region of origin (**c**). **d**, The regional composition of major cell types. **e**, Relative enrichment of major cell types across regions by quasi-binomial regression. False discovery rate (FDR)-corrected *P* values are indicated by asterisks; ***P < 0.001, **P < 0.01, *P < 0.05. **f**,**g**, Global breakdown, region composition, enrichment and number of nuclei for excitatory (**f**) and inhibitory (**g**) neuronal subtypes. **h**, Gene expression analysis of the top four markers per inhibitory subclass, averaged at the sample by subclass level (columns). **i**, RNAscope validation of *FOXP2* and *MEIS2* as markers of the unique thalamus subtype, with quantification (left) performed using Student’s *t*-tests and representative images (right). The blue puncta represent *MEIS2* (top) or *FOXP2* (bottom) transcripts and red puncta represent *GAD2* transcripts. *FOXP2*: *n* = 19 (PFC) and *n* = 22 (TH) cells; *MEIS2*: *n* = 35 (PFC) and *n* = 26 (TH) cells; each dot represents an individual cell, pooled from eight samples (four individuals; each had one PFC and one thalamus sample). **j**, Glutamatergic versus GABAergic scores for all neuron subtypes. The dotted lines represent the 95% confidence interval around the linear fit. *P* values were calculated using two-sided *F* tests. Ast., astrocytes; exc., excitatory neurons; inh., inhibitory neurons; mic., microglia/immune cells; olig., oligodendrocytes; vasc., vascular/epithelial cells.

**Extended Data Fig. 1:**
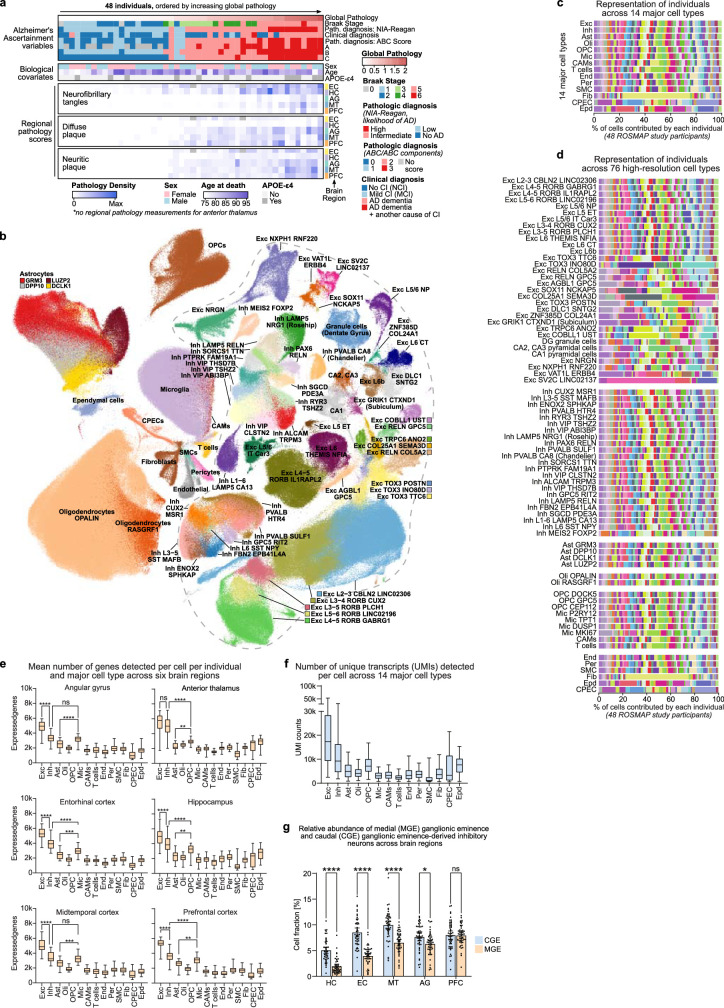
Overview of the study sample and major cell type annotations. **a**, Metadata overview: a total of 283 post-mortem brain tissue samples from 24 male and 24 female study participants were analysed across Alzheimer’s disease progression (AD). Top two panels show metadata at the individual level and bottom three panels show region-specific pathology measurements of neurofibrillary tangle burden (nft), neuritic plaque burden (plaq_n), and diffuse plaque burden (plaq_d). Individuals (columns) are ordered according to their global AD pathology burden. **b**, Joint UMAP of 1.3 M cells across 14 major cell types coloured and labelled by 76 high-resolution subtypes. **c**,**d**, Representation of individuals across cell types. The stacked bar plots show the proportion of cells contributed by each study participant across 14 major cell types (**c**) and 76 high-resolution cell types (**d**). **e**-**f**, Box plots of the number of genes detected per cell across all major cell types (**e**) and mean number of unique transcripts detected per cell per individual and major cell type across the six brain regions analysed (**f**). Within each box, horizontal lines denote median values; boxes extend from the 25th to the 75th percentile of each group’s distribution of values; whiskers extend from the 5th to the 95th percentile. ****P < 0.0001, ***P < 0.001, **P < 0.01; ns, P > 0.05; (ordinary one-way ANOVA corrected for multiple comparisons using Bonferroni’s multiple comparisons test). **g**, Relative abundance of inhibitory neurons originating from the medial (MGE) ganglionic eminences (SST and PVALB) and the caudal (CGE) ganglionic eminence (VIP, PAX6, and LAMP5) across brain regions. The bar plots show the mean fraction of cells per individual and brain region (AG, HC, MT, PFC: n = 48; TH: n = 45; EC: n = 46). The fraction of cells was computed relative to all the cells isolated from a brain region of an individual. Data are expressed as mean with 95% confidence intervals and individual data points are shown (two-way ANOVA corrected for multiple comparisons using Bonferroni’s multiple comparisons test).

**Extended Data Fig. 2:**
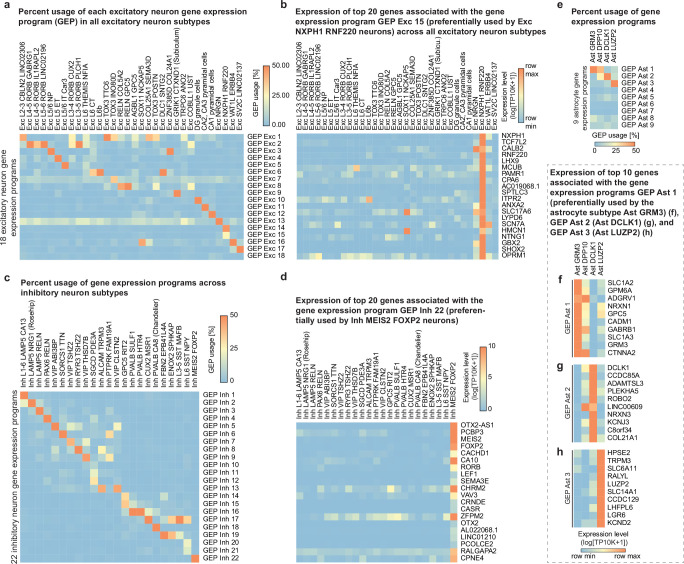
Gene expression programs. **a**, Heat map showing percent usage of all excitatory neuron gene expression programs (GEPs) (rows) in all excitatory neuron subtypes (columns). **b**, relative expression level of the top 20 genes associated with the gene expression program GEP Exc 15 (preferentially used by Exc NXPH1 RNF220 neurons) across all excitatory neuron subtypes. **c**, Heat map showing percent usage of all inhibitory neuron gene expression programs (GEPs) (rows) in all inhibitory neuron subtypes (columns). **d**, Expression level of the top 20 genes associated with the gene expression program GEP Inh 22 (preferentially used by Inh MEIS2 FOXP2 neurons) across all inhibitory neuron subtypes. **e**, Heat map showing percent usage of all astrocyte gene expression programs (GEPs) (rows) in all astrocyte subtypes (columns). **f**-**h**, Relative expression level of the top 10 genes associated with the gene expression programs GEP Ast 1 (preferentially used by the astrocyte subtype Ast GRM3) (**f**), GEP Ast 2 (preferentially used by the astrocyte subtype Ast DCLK1) (**g**), and GEP Ast 3 (preferentially used by the astrocyte subtype Ast LUZP2) (**h**) across all astrocyte subtypes.

**Extended Data Fig. 3:**
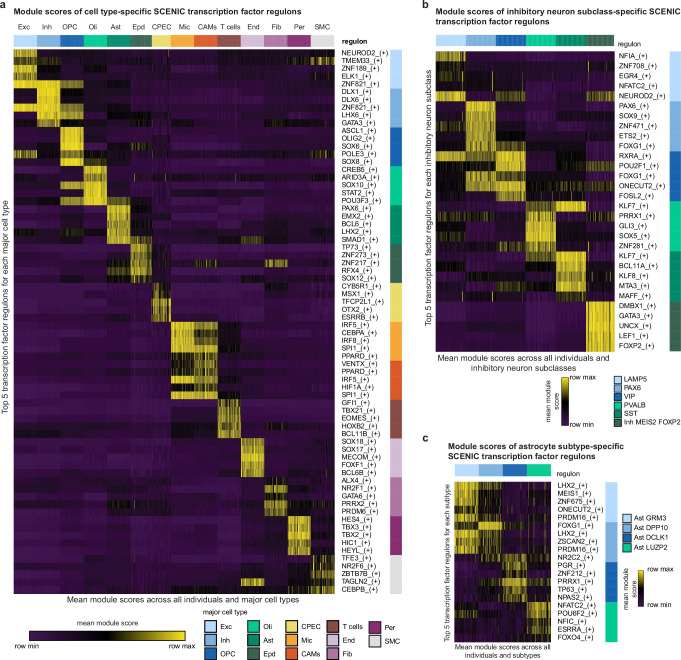
Cell and subtype-specific transcription factor regulators. **a**, Identification of major cell type-specific SCENIC transcription factor regulons. The heat map shows the module score of the top 5 transcription factor regulons (rows) for each major cell type across all individuals and major cell types (columns). **b**, Identification of inhibitory neuron subclass-specific SCENIC transcription factor regulons. The heat map shows the module score of the top 5 transcription factor regulons (rows) for each subclass across all individuals and subclasses of inhibitory neurons (columns). **c**, Identification of astrocyte subtype-specific SCENIC transcription factor regulons. The heat map shows the mean module score of the top 5 transcription factor regulons (rows) across all individuals and astrocyte subtypes (columns).

To characterize cellular diversity in the human brain, and the genes, pathways and cell types that underlie AD progression across brain regions, we performed single-nucleus RNA-sequencing (snRNA-seq) analysis of nuclei isolated from 283 post-mortem brain samples across six brain regions from 48 participants in the Religious Order Study (ROS) or the Rush Memory and Aging Project (MAP)^28^ (together, ROSMAP; Fig. 1a). We selected 48 participants on the basis of pathologic diagnosis of AD (stratified by NIA-Reagan score of 26 (with AD) and 22 (without AD; labelled non-AD)) and on the basis of clinical diagnosis of Alzheimer’s dementia (*n* = 16) versus non-dementia (*n* = 32)^29,30^ (Fig. 1a, Extended Data Fig. 1a and Supplementary Table 1). From these 48 individuals, we profiled six brain regions: the EC (221,493 cells), which is affected in early AD (stages I–II); the HC (221,415) and TH (207,625), which are affected in mid-AD (stages III–IV); and the AG (220,409), MT (227,412) and PFC (254,721), which are affected in late AD (stages V–VI), for a total of 1.35 million transcriptomes of independent nuclei after removing doublets, low-quality cells and highly sample-specific clusters. We annotated 76 high-resolution cell types in 14 major cell type groups, including 32 excitatory neuron subtypes (436,014 nuclei, 32.2% of total) and 23 inhibitory subtypes (159,838 nuclei, 11.8% of total) (Extended Data Fig. 1b–d, Supplementary Figs. 1 and 2 and Supplementary Table 2). We characterized these cell types in terms of their transcriptome size and proliferative status, compared our atlas with previously published data across species^31–33^ (Extended Data Fig. 1e,f and Supplementary Figs. 3–5) and identified broad cell type identity programs using non-negative matrix factorization (NMF)^34^ and transcriptional regulons using SCENIC^35,36^ (Extended Data Figs. 2 and 3 and Supplementary Tables 3 and 4).

To gain insights into the cellular architecture of the human brain, we investigated differences in the composition of major cell types between the six brain regions. The fraction of neurons increased significantly from the TH (14.4% neurons) to the three-layer allocortical HC (32.2%), the entorhinal periallocortex (36.6%) and the six-layered neocortical regions (AG, MT and PFC, 58.9%) (Fig. 1b–e and Supplementary Fig. 6). Glia, including astrocytes, oligodendrocytes, oligodendrocyte precursor cells (OPCs) and microglia/immune cells, tended to be less abundant in neocortical samples (Fig. 1b–e), in agreement with previous studies in humans^37,38^ and mice^39,40^ (Supplementary Fig. 7a–d). Differences in the composition of major cell types between regions were reproducibly observed across study participants, irrespective of the individual’s disease status (Supplementary Fig. 7e–h), suggesting that variability in the major cell type composition between regions is a fundamental characteristic of the human brain and is not affected by AD pathology.

## Neuronal diversity across brain regions

We first characterized the regional diversity of excitatory neuron subtypes, which were consistent across individuals and were either highly region-specific to the HC, EC and TH (7, 9 and 2 subtypes, respectively) or were predominantly shared across neocortical regions (12 subtypes) (Fig. 1f and Supplementary Fig. 8–12). Hippocampal subtypes included neurons from the highly structured CA1 and CA2/CA3 subfields and dentate gyrus and the more entorhinal-proximal subiculum and para/presubiculum areas^9^. EC-specific subtypes that clustered separately from neocortical subtypes for the same layers were often marked by expression of *RELN*, *TOX3* and *GPC5*, and contained subtypes from both the lateral (L2 *RELN*^*+*^*GPC5*^*+*^) and medial (L2 *TOX3*^*+*^*POSTN*^*+*^) EC^41–43^ (Supplementary Fig. 10). Excitatory neurons in the TH were predominantly composed (74%) of a subtype (*NXPH1*^*+*^*RNF220*^*+*^) that was not observed in the neocortex and is predicted to be regulated by *LHX9*, *SOX2*, *SHOX2* and *TCF7L2*^34,36^ (Extended Data Fig. 2a,b and Supplementary Figs. 9e–i and 11n,o). We found that the thalamic–neocortex separation is conserved in mice and recapitulated both this divide and thalamic marker genes in independent single-cell, bulk and microarray data in both mice and humans^8,39,40,43^ (Supplementary Fig. 12).

In contrast to excitatory neuron subtypes, the majority of inhibitory neuron subtypes (22 out of 23 subtypes) were observed in all five cortical regions (Fig. 1g and Supplementary Figs. 13–17), although some inhibitory subtypes had regional biases, including *PVALB*^*+*^*HTR4*^*+*^ and *CUX2*^*+*^*MSR1*^*+*^ (enriched in neocortex), layer 6 *SST*^*+*^*NPY*^*+*^ (EC and HC) and *GPC5*^*+*^*RIT2*^*+*^ (EC), suggesting that there are significant differences in inhibitory neuron composition between the neocortex and allocortex (Fig. 1g and Supplementary Fig. 14). Moreover, in the HC, EC and MT, caudal ganglionic eminence-derived GABAergic neurons (*VIP*^*+*^*LAMP5*^*+*^) were significantly more abundant than medial ganglionic eminence-derived neurons (*SST*^*+*^*PVALB*^*+*^), but these two major clades were not significantly different in the PFC (Extended Data Fig. 1g). By contrast, the TH contained a single unique, thalamus-specific inhibitory subtype (*MEIS2*^*+*^*FOXP2*^*+*^) marked by genes that are involved in neurite outgrowth, such as the semaphorins *SEMA3C* and *SEMA3E*, *DISC1* and *SPON1*, and receptors for serotonin (*HTR2A*), acetylcholine (*CHRM2*, *CHRNA3*) and glutamate (*GRM3*) (Fig. 1g,h and Supplementary Figs. 14 and 15). These genes were in a single inhibitory program (Inh-22, from NMF) that included the SCENIC-predicted subtype regulators *FOXP2* and *LEF1*^34,36^ (Extended Data Figs. 2c,d and 3b). We recapitulated this thalamic difference and program genes in the mouse thalamus and human lateral geniculate nucleus (dLGN) using previously published single-cell data (Supplementary Figs. 16 and 17). To validate the localization and specificity of markers of the thalamic inhibitory neuron subtype, we performed in situ hybridization for both *FOXP2* and *MEIS2* with *GAD2* on TH and PFC post-mortem brain samples from four individuals, and found significant thalamus-specific co-localization of both marker genes with *GAD2* (Fig. 1i).

**Extended Data Fig. 4:**
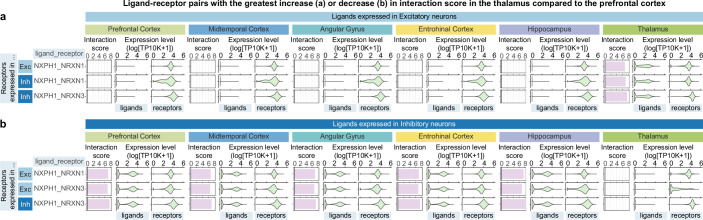
Region-specific cell-cell communication. **a**-**b**, Ligand-receptor pairs with the greatest increase (**a**) or decrease (**b**) in interaction score in the thalamus compared to the prefrontal cortex. Bar plots show the interaction scores for the ligand-receptor pairs indicated. The interaction score was calculated by applying the minus log10 transformation to the robust rank aggregation (RRA) score. A lower RRA score indicates that a ligand-receptor interaction is ranked consistently higher than expected by chance across multiple prediction methods. Violin plots show the expression of the ligand (left) and receptor (right) in the cell types and brain regions indicated.

As thalamic *MEIS2* neurons expressed several typically glutamatergic neuron genes, we determined glutamatergic and GABAergic module scores for every neuronal cell to further examine the chimeric nature of this subtype (Supplementary Fig. 15g–k and Supplementary Table 5). These scores matched the cortical excitatory and inhibitory split and were negatively correlated both across and within broad neuronal classes (Fig. 1j and Supplementary Fig. 15g,h). Both thalamic *MEIS2*^+^ inhibitory and *NXPH1*^+^ excitatory neurons had intermediate scores, placing them between the cortical excitatory and inhibitory clusters, suggesting that they are less polarized with regard to the expression of cortical glutamatergic versus GABAergic programs (Fig. 1j and Supplementary Fig. 15i–k). Predicted cell–cell communication interactions were mostly shared across multiple regions, but the thalamus had multiple differential interactions (Supplementary Figs. 18 and 19). The top thalamus-specific interactions were between excitatory *NXPH1* and neuronal *NRXN1* or *NRXN3*, whereas inhibitory neurons expressed *NXPH1* in the other regions, suggesting that neurexophilin signalling swaps from excitatory neurons in the thalamus to inhibitory neurons in cortical brain regions (Extended Data Fig. 4).

## Glial diversity annotated by gene modules

**Fig. 2:**
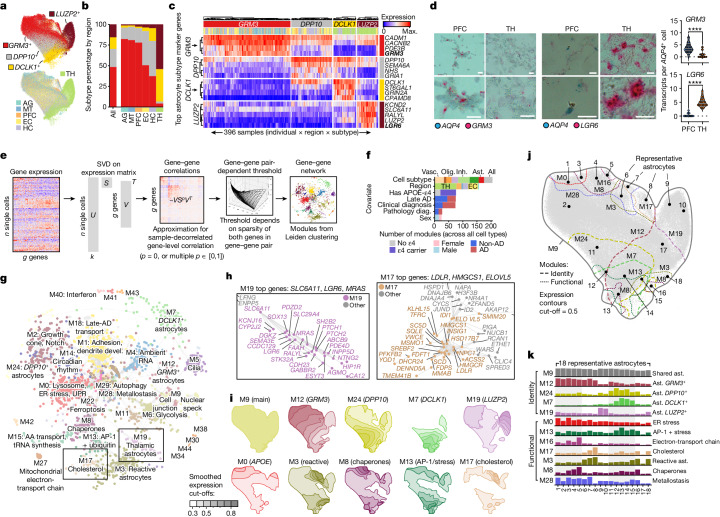
Astrocyte diversity across regions annotated by gene expression modules. **a**, UMAP plot for astrocyte nuclei, coloured by astrocyte subtype or brain region of origin. **b**, Global breakdown and regional composition of astrocyte subtypes. **c**, Gene expression heat map for the top markers of each astrocyte subclass, averaged to sample by subtype and scaled to the row maximum (max.). **d**, RNAscope validation of *GRM3* and *LGR6* as markers of *AQP4*^+^ neocortical and TH astrocytes, respectively (bold markers in **c**). Representative images (left) showing *AQP4* transcripts (blue puncta) and *GRM3* or *LGR6* transcripts (red puncta). Scale bars, 20 μm. Quantification (right) was performed using two-tailed unpaired Student’s *t*-tests; ****P < 0.0001. Each dot represents an individual cell, pooled from eight samples (four individuals; each with one PFC and one thalamus sample). *GRM3*: *n* = 37 (PFC) and *n* = 23 (TH) cells; *LGR6*: *n* = 17 (PFC) and *n* = 23 (TH) cells. **e**, The framework for detecting gene expression modules using scdemon. **f**, The number of modules enriched for each covariate across all module sets (hypergeometric test, *P* < 0.001). Bar plots are coloured by the covariate level for which the modules are enriched (or by the major cell type used for module discovery for cell subtype). Diag., diagnosis. **g**,**h**, Gene–gene network (**g**) and magnification of the indicated regions (**h**) for astrocyte modules, with insets for M19, a subtype identity module for LUZP2 astrocytes (**h**, left) and M17, a functional program involved in cholesterol biosynthesis (**h**, right). AA, amino acid. **i**, Contour plots on the astrocyte UMAP for module expression of five identity (top row) and five functional (bottom row) programs. Expression was smoothed on a 500 × 500 grid with a 2D Gaussian kernel (size = 25 × 25; *σ* = 1). **j**,**k**, Module contours showing regions of top expression on the astrocyte UMAP for selected identity modules (**j**) and corresponding module scores (**k**) for the 18 labelled representative cells across the astrocyte UMAP for selected identity and expression models, scaled to the maximal expression of each module. ER, endoplasmic reticulum; SVD, singular value decomposition.

We next tested whether glial cells also had transcriptional differences between brain regions. We identified multiple transcriptionally distinct subsets for each major glial cell type and determined their characteristic marker genes (Fig. 2a and Supplementary Fig. 20–25). Among glial cell types, astrocytes had the highest regional heterogeneity, containing both highly neocortex-enriched (*GRM3*^+^*DPP10*^+^) and thalamus-enriched (*LUZP2*^+^*DCLK1*^+^) subtypes (Fig. 2a–c and Supplementary Fig. 20). Region-specific astrocyte subtypes were experimentally validated using RNA in situ hybridization (Fig. 2d) and confirmed by analysing a separate snRNA-seq dataset^14^ (Supplementary Fig. 23m). Cortical astrocytes were enriched for markers involved in glutamate processing and transport, whereas hippocampus- and anterior thalamus-enriched *DCLK1* astrocytes had lower glutamate transporter activity and were enriched instead for focal-adhesion-related genes (Fig. 2c and Supplementary Fig. 25a). Thalamic astrocytes (*LUZP2*^+^) expressed GABA-uptake genes *SLC6A1* and *SLC6A11* at much higher levels compared with other subtypes, even though the proportion of inhibitory neurons was not markedly higher in the thalamus (Fig. 2c). Notably, the thalamic *MEIS2*^+^*FOXP2*^+^ interneurons shared multiple markers with neocortical *GRM3* astrocytes, including *GRM3*, *MEIS2* and *VAV3*^39,40,43^ (Supplementary Fig. 23n), suggesting that astrocytes in evolutionarily newer regions may share some functions with inhibitory neurons in older regions.

**Extended Data Fig. 5:**
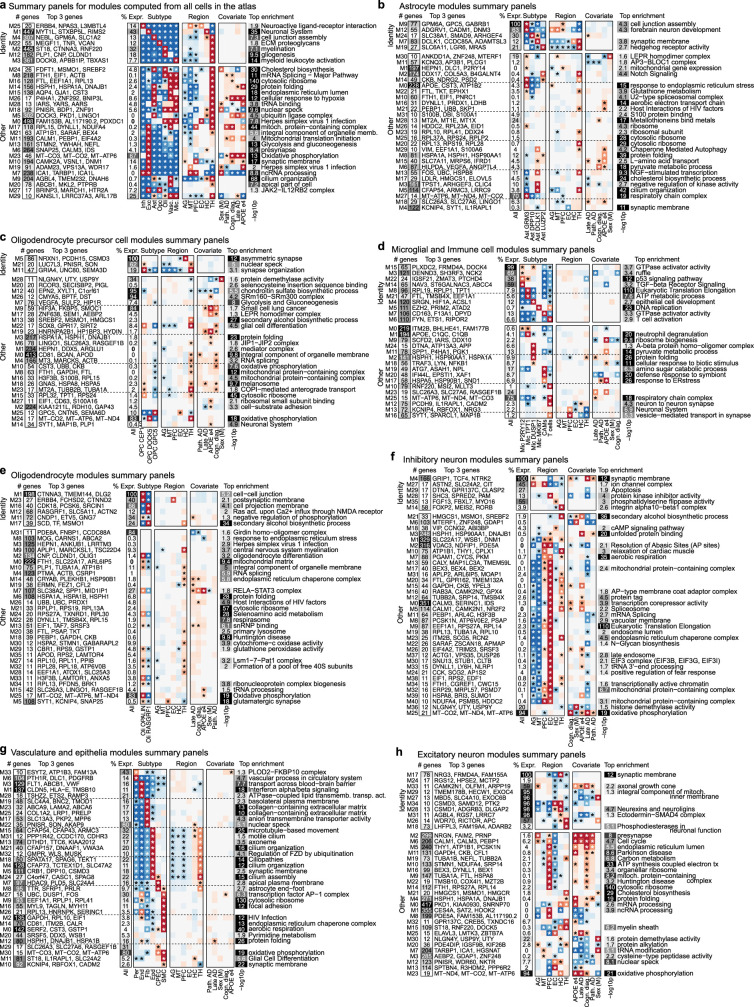
Module summary panels across modules. **a**-**h**, Overview of gene expression modules with at least 10 genes each across all cells and across major cell types, showing the module name, number of genes, percent expression, top module genes, enrichments by subtype (except for neuron subtypes, see Supplement), covariates, and regions, and the top functional enrichment for each module. Percent expression is the percent of cells whose average expression (log1p, normalized) of the module is above 1. Covariate enrichments are performed by hypergeometric test, comparing the intersection of cells with z-scored module expression of at least 1 vs. with z < 1 against a particular level of a covariate of interest (e.g. cells from the entorhinal cortex region or cells of a specific subtype). Panels summarize modules found in all cells (**a**), astrocytes (**b**), OPCs (**c**), microglia and immune cells (**d**), oligodendrocytes (**e**), inhibitory neurons (**f**), vasculature and epithelia (**g**), and excitatory neurons (**h**). All modules except vasculature and epithelia modules are split into identity vs. other, where identity modules are highly enriched in a single subtype and have an average expression greater than 1 (log1p, normalized) for over 50% of the subtype’s cells.

**Extended Data Fig. 6:**
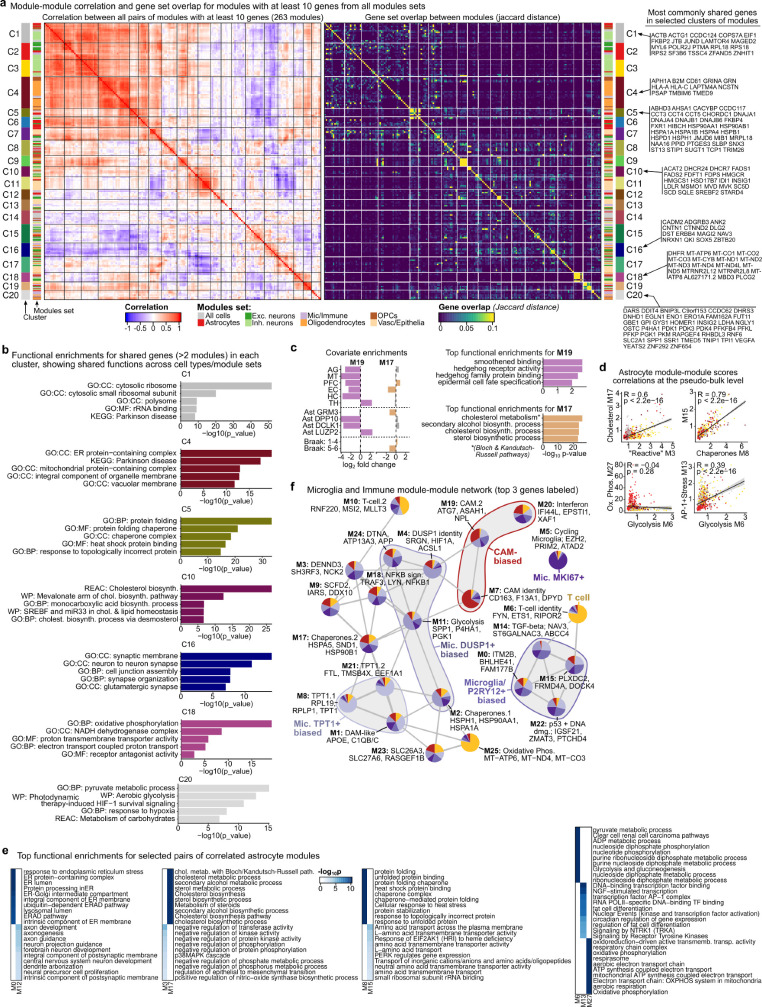
Cross-module clustering and comparison. **a**, Module-module correlation (Pearson correlation) and gene set overlap (Jaccard distance) for modules with at least 10 genes from all sets of modules (263 modules in total). Heatmaps are ordered by the hierarchical clustering of the correlation matrix and cuts represent 20 clusters cut from the hierarchical clustering dendrogram. Left and right side bars label rows by their modules set of origin (major cell type colours and grey for all cells). The most commonly shared genes in selected clusters of modules are shown on the right of the gene set overlap heatmap. **b**, Functional enrichments for each cluster of modules for the shared genes (>2 modules) in each cluster (only clusters with significant enrichments shown). Up to 5 enrichments shown, ordered by p-value, labelled by their source and only keeping terms with fewer than 500 genes. **c**, Covariate and functional enrichments for example astrocyte modules M19 (thalamus identity program) and M17 (cholesterol metabolism and biosynthesis program). Region, subtype, and covariate enrichments performed at cell level by stratifying cells with z-score > 1 and testing for regional or subtype enrichment (see Methods). Functional enrichments performed using gprofiler2, keeping terms with fewer than 500 genes. **d**, Scatterplots and correlation of scores for selected pairs of astrocyte modules. Each dot represents the module expression scores for a subtype in a specific sample and is coloured by the astrocyte subtype. Grey area represents the 95% confidence interval around the linear fit. **e**, Functional enrichments for selected astrocyte modules, showing top 10 functional enrichments for each pair of compared correlated modules (and for M6, M13, M27 together). Only terms with fewer than 500 genes shown. **f**, Microglial and immune modules network from correlation of module pairs at the subtype by sample level (edges shown where FDR-adjusted p-value < 0.05). Nodes are coloured by module’s relative expression in each of the microglial and immune subtypes and groups highlight sets of subtype-biased modules.

We developed a method, single-cell decorrelated module networks (scdemon), to identify gene expression modules from highly correlated sets of genes in atlas-scale snRNA-seq datasets (Fig. 2e). Highly imbalanced cell type composition in single-cell datasets, in which rare cellular states are outnumbered by common cell types, can lead to under-recovery of gene–gene interactions, especially for genes that are expressed at low levels. To account for these issues, our method estimates a sample-decorrelated gene–gene correlation matrix, thresholds gene–gene pairs based on their sparsity and uses the adjusted matrix to identify modules of highly correlated genes (Methods). We used our method to identify modules both across all cells in the atlas and for each major cell type independently, and recovered modules expressed to varying degrees, ranging from identity modules for each glial cell type to a cell cycle module found in just 0.7% of microglia (Fig. 2f, Extended Data Fig. 5, Supplementary Figs. 26–36 and Supplementary Table 6). Cells expressing these modules were enriched for diverse aspects of our dataset, including cellular subtype identity (205 modules), brain region (156, with 77 thalamus specific and 34 EC specific), AD status (73), *APOE* genotype (78) and sex (24) (Fig. 2f and Supplementary Table 7). We hierarchically clustered modules across the cell types and found that many cell types expressed gene programs for cholesterol biosynthesis (C10), chaperones (C5), ribosomes (C1 and C2), ER protein processing (C7), oxidative phosphorylation (C18), synapse interaction (C16), and glycolysis and response to hypoxia (C20) (Extended Data Fig. 6a,b).

Using this approach, we identified 32 modules in astrocytes, including an astrocyte-wide program (M9, expressed in >99% of astrocytes) marked by *GPM6A* and *GPC5* and enriched for cell junction assembly, and subtype- and region-specific identity programs such as thalamus-associated M19 (*SLC6A11*, *LGR6*, *MRAS*), which were enriched for sonic hedgehog signalling, M12 (*GRM3*: forebrain neuron development) and M7 (*DCLK1*: synaptic membrane) (Fig. 2g,h and Supplementary Fig. 31). Other modules spanned a diverse set of functions, including metallostasis, RNA splicing, glycolysis, oxidative phosphorylation and cholesterol biosynthesis and were shared by multiple subtypes (Fig. 2i–k and Extended Data Figs. 5b and 6a–c). For example, chaperone-enriched and APOE-ε4-associated M8 was expressed in multiple different astrocyte subtypes and regions, and expression of AD-associated M28 (metallostasis) overlapped with expression of both *APOE*^+^ (M0) and reactive (M3) astrocytes (Fig. 2i–k). Module–module correlations across samples revealed co-expressed programs, such as reactive astrocytes (M3, marked by *TPST1*, *CLIC4* and *EMP1*) with cholesterol biosynthesis module M17 (*r* = 0.60), and glycolysis (M6) with AP-1 module M13 (*r* = 0.39, including *FOS*/*JUN* and ubiquitin), a pair that is potentially co-expressed in astrocytes under metabolic stress (Extended Data Fig. 6d,e).

In contrast to astrocytes, immune cells showed little regional specificity and oligodendrocyte-lineage cells had thalamus-enriched subtypes with minor transcriptomic differences to neocortex-enriched subtypes (Supplementary Figs. 21–25). Immune modules included identity programs, such as for T cells (M6), macrophages (M7) and cycling microglia (*MKI67*^+^, M5) as well as modules found across immune cells and enriched for genes involved in NF-κB signalling (M18), interferon (M20), p53 and DNA damage response (M22) and TGFβ signalling (M14) (Extended Data Fig. 6f and Supplementary Fig. 32). Oligodendrocyte-lineage modules showed high regional specificity, and two OPC modules—thalamus-enriched M11 and EC-enriched M25—were marked by synapse-associated genes such as neural adhesion-related *SEMA3D*, *SEMA6D* and *CNTN5*, and glutamate receptor *GRIA4*, suggesting a role for OPC sensation and response to neuronal activity in specific brain regions (Extended Data Fig. 5c,e and Supplementary Figs. 33, 34 and 36).

## Vulnerable neuronal subtypes in AD

**Fig. 3:**
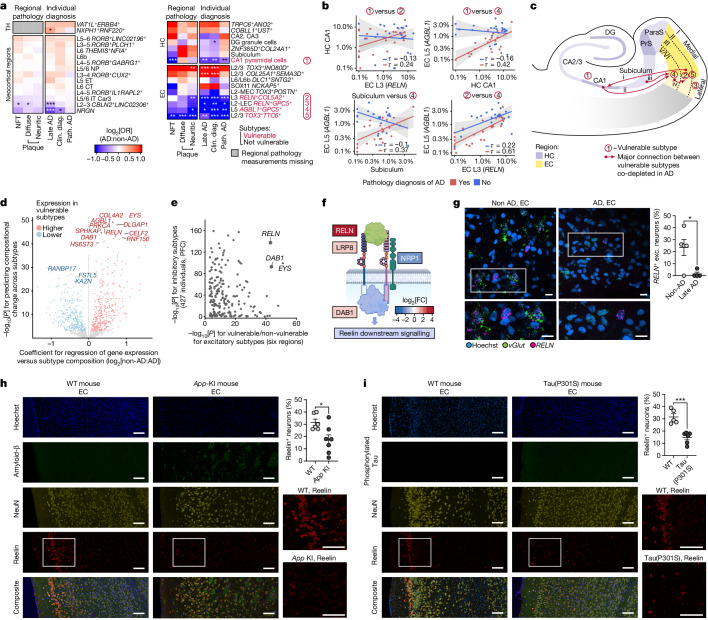
Subtype-specific neuronal vulnerability in AD. **a**, Compositional differences in excitatory neuron subtype enrichment and depletion in AD by quasi-binomial regression with FDR correction. Clin. diag., clinical diagnosis; path. AD, pathologic AD. **b**, Scatter plot and correlations (Kendall’s *τ*) of the subtype fraction between four pairs of neuronal subtypes in the HC and EC (linear fit with 95% confidence intervals). **c**, Schematic of the HC and EC, highlighting the locations of vulnerable excitatory subtypes and co-depleted connections. **d**, Genes associated with excitatory neuron subtype vulnerability across all brain regions. Linear regression between normalized sample + subtype-level gene expression and log_2_[OR] for late-AD, with FDR-corrected *P* values. **e**, Genes associated with excitatory and inhibitory subtype vulnerability (FDR-corrected *P* values, only genes significantly and positively associated with excitatory subtype vulnerability). **f**, Schematic of Reelin signalling pathway genes that are differentially expressed in vulnerable inhibitory subtypes (colour indicates the log_2_-transformed fold change in expression between vulnerable and non-vulnerable subtypes). The diagram was created using BioRender. **g**, In situ hybridization (RNAscope) validation of depletion of *RELN*^*+*^ excitatory neurons in the EC of individuals with AD relative to individuals without AD. Representative images (left) include Hoechst (blue), *vGlut* transcripts (green puncta) and *RELN* transcripts (magenta puncta). Scale bars, 20 μm. Quantification (right) was performed using unpaired two-tailed Student’s *t*-tests (*P* = 0.0242). Data are mean ± s.e.m. *n* = 5 (non-AD) and *n* = 4 (AD) individuals. **h**,**i**, Immunohistochemistry analysis of Reelin, NeuN and amyloid-β (**h**) or phosphorylated tau (**i**) in 12-month-old *App-KI* mice (**h**) or 9-month-old Tau(P301S) transgenic mice (**i**), showing depletion of Reelin-positive neurons in the ECs of the KI and transgenic mice compared with those of the wild-type controls. Representative images (left) show Hoechst (blue); amyloid-β (**h**; D54D2) or phosphorylated-tau (**i**) (green); NeuN (yellow); and Reelin (red). Scale bars, 100 μm (**h** and **i**). Quantification (right) was performed using unpaired two-tailed Student’s *t*-tests; *P* = 0.0181 (*App*-KI, **h**; *n* = 7 (*App*-KI) and *n* = 6 (wild type) mice) and *P *= 0.0005 (Tau(P301S), **i**; *n* = 6 mice (Tau(P301S)) and *n* = 5 (wild type) mice). Data are mean ± s.e.m. ParaS, parasubiculum; PrS, presubiculum. Source Data

**Extended Data Fig. 7:**
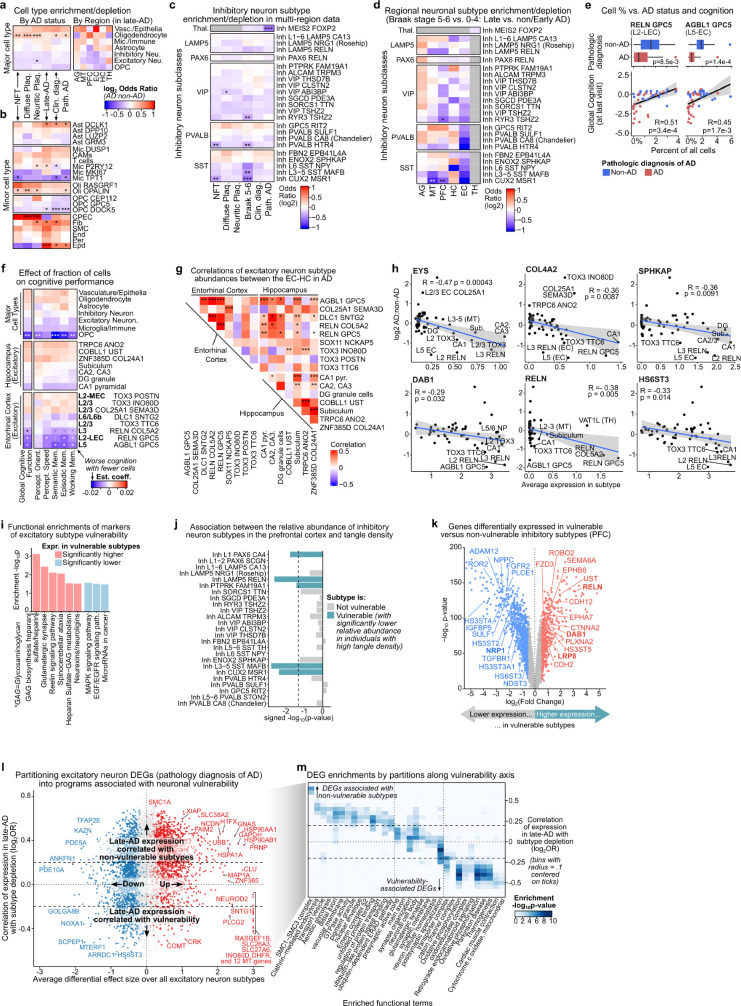
Neuronal vulnerability, connectivity, and markers of vulnerability. **a**, Compositional differences for major cell types in AD by quasi-binomial regression with FDR-correction. log_2_ OR shown both for each AD variable across regions (left) and for each region in late-AD (right). Analysis performed for individual-level AD status and region-level pathology measurements. Pathologic diagnosis of AD (Path. AD) was stratified by NIA-Reagan score (26 AD and 22 non-AD) and clinical diagnosis was stratified as AD dementia (n = 16) and non-CI (n = 32). **b**-**c**, Compositional differences for glial subtypes and inhibitory neuron subtypes according to individual-level AD status and region-level pathology measurements (as in **a**). Grey boxes indicate interactions that are not computed due to MEIS2 FOXP2 specificity to the thalamus, where we do not have measured regional scores. **d**, Compositional differences in inhibitory neuron subtypes in late AD (Braak Stage 5-6 vs. 1–4) in each region. Grey boxes indicate interactions that are not computed due to subtype regional specificity. **e**, Boxplots (top) of neuronal fraction for two vulnerable EC subtypes, split by AD status (AD: blue, non-AD: red), with p-values from one-sided Wilcoxon test. Scatter plots (bottom) of individuals’ global cognition at last visit against cell fraction for two AD-vulnerable entorhinal cortex subtypes, coloured by AD. Linear fit with 95% confidence interval shown in grey. **f**, Estimated effect size of cell fraction (log_10_) on scores for performance in various cognitive domains at last visit and combined scores from all domains (global). Linear regression FDR-corrected p-values (**adjusted p-value < 0.01, *<0.05, dot is <0.1). **g**, Full correlation matrix between subtype fraction between the hippocampus and entorhinal cortex in the same individuals, as described in the methods (***adjusted p-value < 0.001, **<0.01, *<0.05). **i**, Example genes predictive of subtype vulnerability. Scatterplots show average expression in the subtype across individuals against the effect size of the depletion or enrichment in AD as measured by the log_2_ odds-ratio for late-AD, as in the Methods. **i**, Functional enrichments and intersected genes for top 30 markers of subtype vulnerability (terms with <500 genes). **j**, Association (quasi-binomial regression) between the relative abundance of inhibitory neuron subtypes in the prefrontal cortex and the density of neurofibrillary tangles. Association scores (signed negative log10 FDR-adjusted P value, where the sign was determined by the direction (positive or negative) of the association) are shown. The dotted line indicates the significance level threshold of an FDR-corrected P value of 0.05. P values were derived using the glm function in R and adjusted for multiple testing via the Benjamini-Hochberg method. **k**, Volcano plot showing genes differentially expressed in vulnerable versus non-vulnerable inhibitory neuron subtypes (genes significantly higher in vulnerable subtypes in red, lower in blue). FDR-adjusted P values as determined by the R package ‘dreamlet’ are shown. **l**, Scatter plot of each tested gene’s average differential expression effect size in late-AD (y-axis) versus the correlation of its expression in a subtype and that subtype’s level of depletion in late-AD (x-axis). Dashed lines separate genes associated with vulnerability and non-vulnerability. **m**, Functional enrichments for each identified class of neuronal DEGs (terms <500 genes) on bins (along x-axis from **l**), from genes associated with vulnerability to those associated with non-vulnerability (only genes with biased effect sizes, see Methods). Dashed lines correspond to the same breaks as in (**l**).

After constructing our atlas across AD-affected brain regions, we examined how AD affects the cellular composition. At the level of major cell types, we observed slight, non-significant decreases in the number of both excitatory neurons (odds ratio (OR) = 0.94, individuals stratified by pathologic diagnosis of AD), inhibitory neurons (OR = 0.93) and OPCs (OR = 0.85), as well as an increase in the number of oligodendrocytes (OR = 1.14, adjusted *P* (*P*_adj_) = 0.01) and vascular cells (OR = 1.24), mostly driven by differences in the EC, HC and PFC regions, especially in late AD (Extended Data Fig. 7a,b). We next tested whether the fractions of region-specific neuronal subtypes were significantly altered relative to both individual-level pathologic and clinical diagnoses of AD and region-level NFT and plaque accumulation (Fig. 3a and Extended Data Fig. 7c,d). Among excitatory neurons, we identified one HC-specific (CA1 pyramidal neurons) and four EC-specific (L2 *RELN*^+^ lateral EC, L3 *RELN*^+^, L5 and L2/3 *TOX3*^+^*TTC6*^+^ neurons) subtypes that were significantly less abundant (OR = 0.38–0.66) in individuals with a pathologic diagnosis of AD (Fig. 3a and Supplementary Fig. 37a–c). Neocortical L2–3 neurons were also significantly less abundant in samples with high NFT levels and in individuals with neocortical NFT involvement (Fig. 3a). Individuals with lower percentages of these vulnerable excitatory neuron subtypes performed significantly worse on cognitive tasks, with the strongest observed impacts on episodic memory and global cognitive function for subtypes marked by *GPC5* (EC L5 and L2 *RELN*^+^)^2^ (Extended Data Fig. 7e,f). Notably, while the overall excitatory fraction was not associated with cognition, lower OPC fraction across regions and, in particular, in non-neocortex regions was significantly associated with impaired cognition (Supplementary Fig. 37d).

Given that these neuronal subtypes lie in highly interconnected regions, we next examined whether neuronal subtypes connected across regions were coordinately depleted. We found that vulnerable neuronal subtypes were co-depleted specifically in individuals with AD, with some of the strongest effects observed in established connections between the CA1, subiculum, EC–L3 and EC–L5 (Fig. 3b,c and Extended Data Fig. 7g). These included co-depletion for entorhinal L5 versus L5-projecting subiculum (Kendall’s *τ* = 0.37 (AD); −0.1 (non-AD)) or CA1 (*τ* = 0.42 (AD) and −0.16 (non-AD)); and for CA1 versus L2-lateral EC (LEC, *τ* = 0.26 (AD) and −0.07 (non-AD)) and L3 *RELN*^+^ (*τ* = 0.24 (AD) and −0.13 (non-AD)) EC neurons, both of which project in part to the CA1 subfield^44,45^ (Fig. 3b,c and Extended Data Fig. 7g).

We next investigate whether vulnerable subtypes share marker genes that might mediate their vulnerability, and identified 391 genes with significantly higher baseline (non-AD) expression in vulnerable subtypes (Fig. 3d and Supplementary Table 8). These included Reelin signalling pathway genes *RELN* and *DAB1*; kinase-associated genes *MAP2K5*, *PRKCA* and *SPHKAP*; and multiple genes associated with heparan sulfate proteoglycan biosynthesis (including *HS6ST3*, *XYLT1* and *NDST3*) (Fig. 3d and Extended Data Fig. 7h,i). Notably, while *RELN* expression, which is typically restricted to inhibitory neurons, was highly specific to two EC excitatory subtypes, its downstream partner *DAB1* was present across subtypes (Extended Data Fig. 7h,i and Supplementary Fig. 37e,f).

We next examined whether vulnerable inhibitory neuron subtypes in the PFC share characteristics with vulnerable excitatory neuron subtypes across our brain regions using single-cell transcriptomes from 621 ROSMAP study participants^27,46^. We identified specific inhibitory neuron subtypes that are depleted in individuals with a high tangle density burden, consistent with our previous findings^27^ (Extended Data Fig. 7j). Vulnerable and non-vulnerable inhibitory neuron subtypes differed in the expression of genes involved in neuron projection morphogenesis (*ROBO2*, *SEMA6A* and *EPHB6*), enzyme-linked receptor protein signalling pathways (*FGFR2*, *TGFBR1* and *PLCE1*) and heparan sulfate proteoglycan biosynthesis (Extended Data Fig. 7k and Supplementary Table 8). Notably, vulnerable inhibitory neuron subtypes expressed significantly higher levels of the Reelin signalling pathway components *RELN* and *DAB1*, mirroring the observed higher expression of these two genes in vulnerable excitatory neuron subtypes (Fig. 3e). Furthermore, the Reelin receptors LRP8 (also known as ApoER2) and NRP1 exhibited significantly different baseline expression in vulnerable compared with non-vulnerable inhibitory neuron subtypes (Fig. 3f).

To test the selective vulnerability of Reelin-expressing excitatory neurons in AD, we performed in situ hybridization (RNAscope) analysis of Reelin and vGlut (excitatory neuron marker) in EC tissue samples from both patients with AD and healthy individuals without AD. We found a significant decrease in the percentage of Reelin-expressing excitatory neurons in the EC of individuals with AD (Fig. 3g). To determine whether this finding was conserved in animal models of AD, we used immunohistochemistry to assess the expression of Reelin in the EC of both 12-month-old *App* knock-in (KI) mice and 9-month-old Tau(P301S) transgenic mice. We found that, relative to wild-type littermate controls, *App*-KI mice and Tau(P301S) mice had a significantly decreased percentage of Reelin-positive neurons in the EC (Fig. 3h,i), in agreement with our human transcriptomic data suggesting a selective vulnerability of Reelin-expressing neurons (Fig. 3d–f).

To understand how vulnerable subtypes are altered in AD, we computed differentially expressed genes (DEGs) for each excitatory neuron subtype (Methods and Supplementary Fig. 38a–c). We partitioned DEGs into sets associated with either vulnerable or non-vulnerable subtypes according to their expression levels in individuals with late AD (Extended Data Fig. 7l,m). DEGs linked to non-vulnerable subtypes were enriched for a diverse set of functions, including ubiquitin-ligase binding, heat-shock-family chaperones, ER protein processing and mediators of neuronal death, whereas vulnerability-associated DEGs were highly enriched only for mitochondrial oxidative phosphorylation but included *CRK* and *NEUROD2*, which are both associated with Reelin signalling^17,18^ (Extended Data Fig. 7l,m and Supplementary Fig. 38d–f). Some DEGs associated with non-vulnerable subtypes had higher differential effect sizes in the vulnerable subtypes, and showed additional enrichment for aerobic glycolysis (including *PGK1*, *LDHB* and *SLC2A3*) and clathrin-mediated endocytosis (including *AP2M1*/*AP2S1*, *OCRL* and *COPS8*) (Extended Data Fig. 7m).

## Regional expression differences in AD

**Extended Data Fig. 8:**
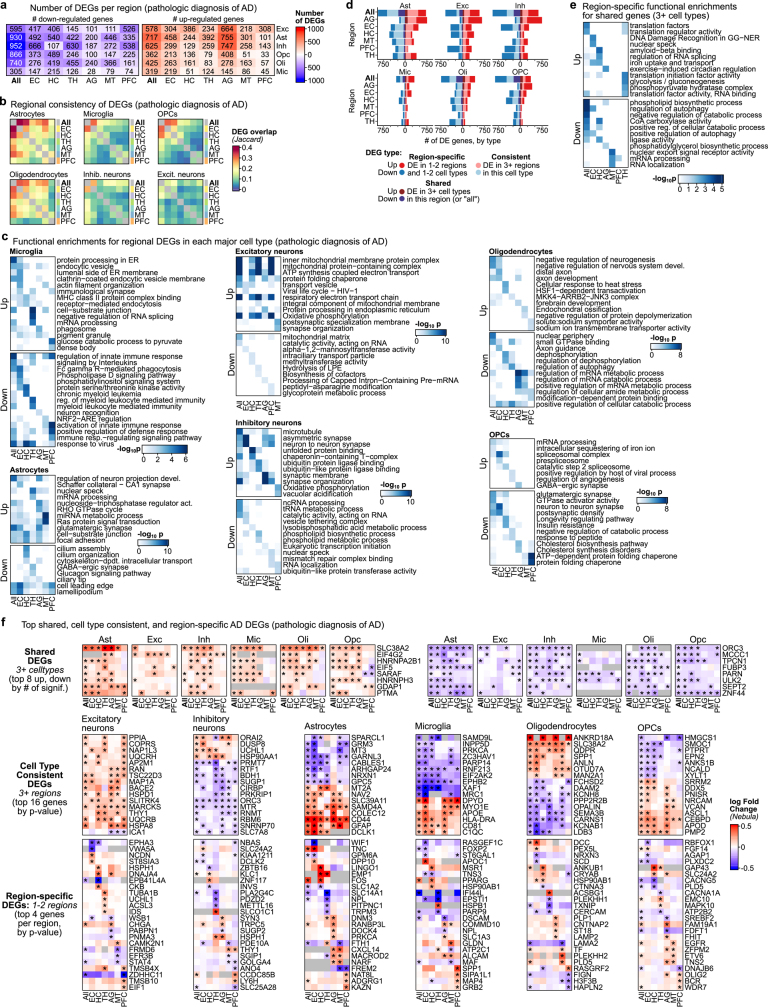
Regional differential expression and GWAS association. **a**, Number of up- and down-regulated differentially expressed genes (DEGs) with respect to pathologic diagnosis of AD for each major cell type, calculated in each region separately as well as jointly over all regions. **b**, Heatmaps of Jaccard similarity of DEGs across regions for each major cell type. **c**, Heatmap of -log_10_ p-values for functional enrichments showing the top pathways for AD DEG shared across 3+ cell types. Enrichments shown for DEGs calculated in each region and in all regions together (up to the top 3 pathways per analysis are shown). **d**, Barplot of number of DEGs per region and cell type, coloured by type of DEG, as determined by its shared differential expression across regions and cell types. **e**, Top functional enrichments for region-specific DEGs and DEGs shared across regions (≥3 regions) with up to the top 2 terms (<500 genes only) shown per region. Panels shown and computed separately for each major cell type. **f**, Heatmap of log fold change for top shared, cell-type consistent, and cell+region-specific DEGs in major cell types. GG-NER: global genome nucleotide excision repair.

To identify regional differences in cellular expression and function specific to individuals with pathologic AD, we computed DEGs for each major cell type in every region alone and across regions using a negative binomial linear mixed model framework, adjusting for both known covariates and potential unknown batch effects (Methods) (Extended Data Fig. 8a and Supplementary Table 9). Astrocytes and inhibitory and excitatory neurons showed the highest number of DEGs over all of the regions, with the largest number of changes in the EC (Extended Data Fig. 8a). Notably, neuronal DEGs showed little overlap across regions, indicating that neuronal differences in AD are primarily determined by subtype or region of origin (Extended Data Fig. 8b). By contrast, microglia and OPC DEGs overlapped within the non-neocortex regions, and astrocyte and oligodendrocyte changes were more consistent across all regions (Extended Data Fig. 8b). AD DEGs were consistent with published results both for region-specific DEGs and for DEGs computed jointly over all regions for multiple AD variables, and were further corroborated by comparisons with various independent studies^11,12,15,19,47–53^ (Supplementary Fig. 39).

Excitatory DEGs were strongly enriched for electron-transport functional terms across regions and showed weak region-specific enrichments for protein-folding-, ubiquitination- and synapse-associated terms (Extended Data Fig. 8c). Inhibitory DEGs were also broadly enriched for protein-folding- and synapse-associated terms and for oxidative phosphorylation uniquely in the thalamus (Extended Data Fig. 8c). While microglia DEGs were broadly enriched for clathrin-coated endocytosis (up) and viral response (down), they also had diverse region-specific enrichments, including upregulation of major histocompatibility complex type II (MHC-II) binding in the EC and HC, RNA processing in thalamus and glycolysis in the PFC and EC; and HC-driven downregulation of phagocytosis, phospholipase signalling and protein kinase activity (Extended Data Fig. 8c).

**Extended Data Fig. 9:**
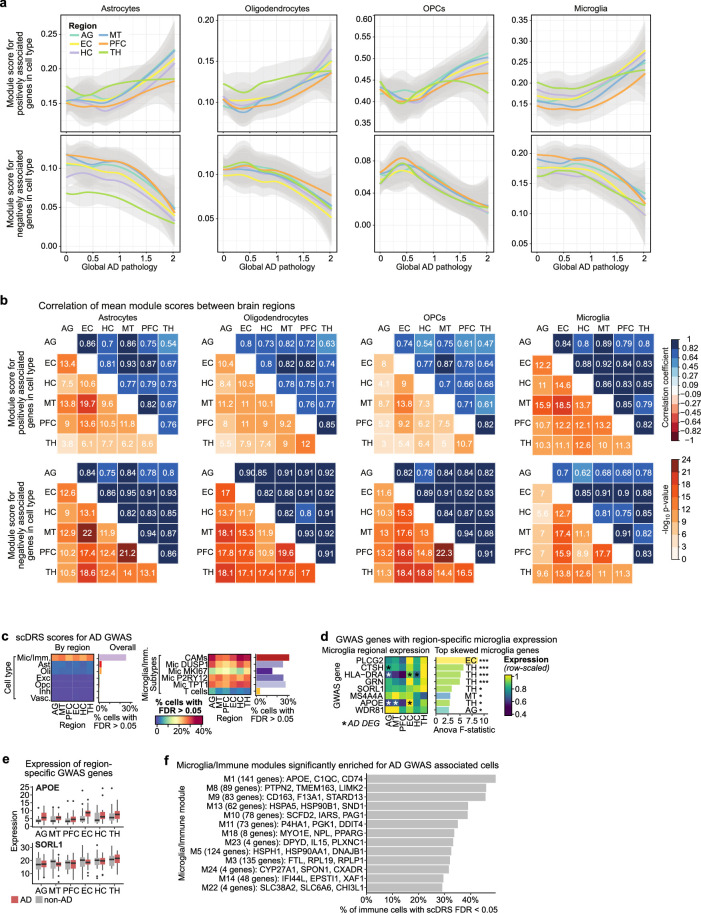
Inter-regional comparison of AD pathology-associated gene sets and region-specific GWAS enrichments. **a**-**b**, Seurat module scores of genes significantly positively (top) or negatively (bottom) associated with the global AD pathology variable in prefrontal cortex for astrocytes, oligodendrocytes, OPCs, and microglia across brain regions and the spectrum of global AD pathology burden. The gene sets used for computing the module scores (genes significantly associated with global AD pathology burden) were determined based on snRNA-seq data derived from prefrontal cortex tissue of 427 ROSMAP study participants. The scatterplots (**a** panels) illustrate the relationship between global AD pathology burden and the mean module score for the specified gene set, with this mean score calculated by averaging the module scores of all cells of the designated cell type isolated from an individual. A LOESS (Locally Estimated Scatterplot Smoothing) regression line with a 95% confidence interval is shown, and the regression lines are coloured by brain region. The central LOESS regression line represents the local measure of central tendency, calculated through locally weighted regression to reflect the smoothed relationship between the module scores indicated and global AD pathology burden. Interregional Pearson’s correlation analysis of mean module scores (**b** panels) was performed by first averaging the module scores of all cells of the cell type of interest from an individual study participant. The correlation analysis was then performed between regions based on these averaged scores. P values were calculated using the cor.test function in R and were adjusted for multiple testing using the p.adjust function with the Benjamini-Hochberg method. **c**, Heatmap (by region, left) and barplot (over all regions, right) showing the percentage of cells with significant scDRS (disease relevance scores) for AD GWAS. Rows are split into major cell type groups (top) and microglia and immune subtypes. **d**, Regional expression (heatmap, left) and F-statistic for region in predicting expression (barplot, right) for eight GWAS genes with significantly region-specific expression in microglia. Barplot is coloured by the top expressed region (regression coefficient). Heatmap is labelled with stars if the gene is a DEG for that region. **e**, Boxplots showing expression of two of the region-specific GWAS genes in individuals with and without a pathologic diagnosis of AD. **f**, Microglia/immune modules associated with AD GWAS. Fraction of microglia or immune cells with significant expression of each module (z-score > 2.5) and with significant scDRS scores (FDR < 0.05). Only significant modules are shown (adjusted p < 0.01, hypergeometric test with BH correction).

The majority of region-specific DEGs was either broadly shared (on average, 11% of genes were differentially expressed in 3+ cell types in a region) or were in cell-type-specific programs (40% of DEGs were in 3+ regions for a cell type) (Extended Data Fig. 8d,e). Such genes included *SLC38A2* and *EIF4G2* (broadly shared across regions) and *PRDX5*, *HLA-DRA* or *CD44*, upregulated DEGs in excitatory neurons, microglia and astrocytes, respectively (Extended Data Fig. 8f and Supplementary Fig. 40). Broadly shared genes across cell types showed region-specific enrichment, including for DNA damage (EC), amyloid-β binding and iron transport (HC) and glycolysis (thalamus) in upregulated genes as well as for phospholipid biosynthesis and autophagy in downregulated genes (Extended Data Fig. 8e). Gene sets based on DEGs for global AD pathology burden in the PFC across 427 individuals changed consistently in each region and glial cell type across global pathology, indicating that a significant component of the glial AD response is consistent across regions^27^ (Extended Data Figs. 8b–e and 9a,b). The remaining regional DEGs (on average, 48% of DEGs) highlighted region- and cell-type-specific changes. In microglia, these included *PPARG* and *MSR1*, upregulated in the HC, each associated with microglia polarization, as well as upregulation of lipoprotein modifier *APOC1* and downregulation of transcription factor *FOXP2* in the EC (Extended Data Fig. 8f and Supplementary Fig. 40).

We next examined which cell types and regions were most enriched for genes identified in genome-wide association studies (GWASs) of AD by computing GWAS scores for each cell using single-cell disease-relevance score (scDRS)^54,55^. Microglia and immune cells showed consistently high scores across regions, with the top scores for the microglia *TPT1*^+^ subtype and macrophages in the HC, thalamus and AG (Extended Data Fig. 9c). We examined whether GWAS genes showed region-specific differences in expression that might be linked to the region specificity of AD progression. We identified eight GWAS genes with region-specific expression in microglia, including *PLCG2* (EC), *APOE* and *SORL1* (thalamus), and *MS4A4A* (midtemporal cortex) (Extended Data Fig. 9c–f).

**Extended Data Fig. 10:**
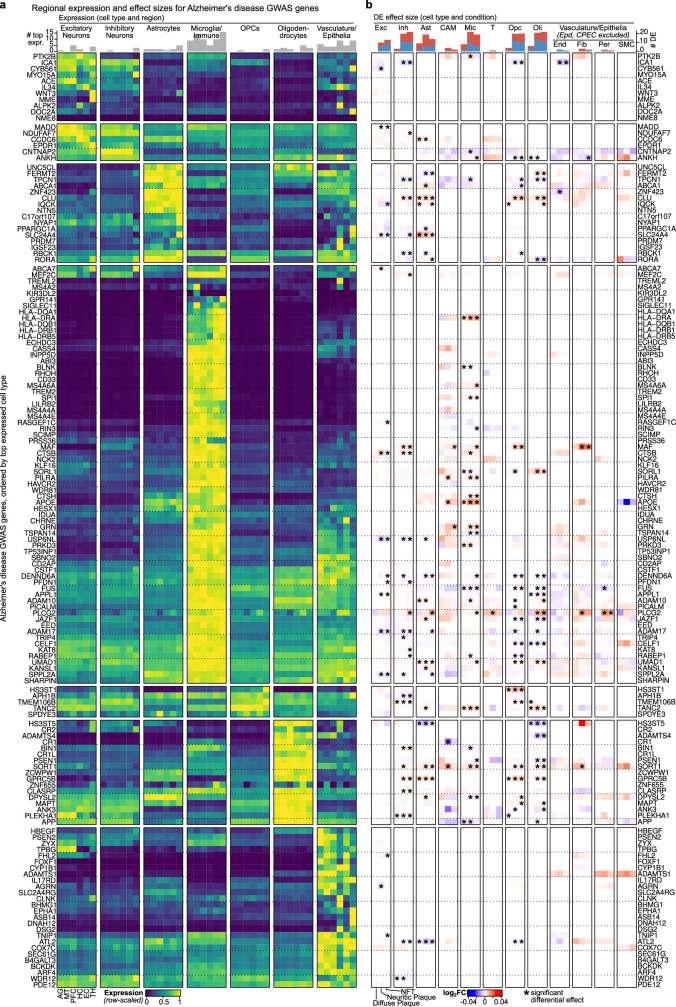
Alzheimer’s disease GWAS-linked genes in the multi-region atlas. **a**, Expression level by region/subtype and effect sizes of 150 Alzheimer’s disease candidate risk genes from Alzheimer’s disease GWAS risk loci. **b**, Differential effect sizes and significance for each candidate risk gene in each minor cell type across regional pathology measurements. Ependymal cells and CPEC cells were excluded as the thalamus does not have regional pathology measurements.

**Extended Data Fig. 11:**
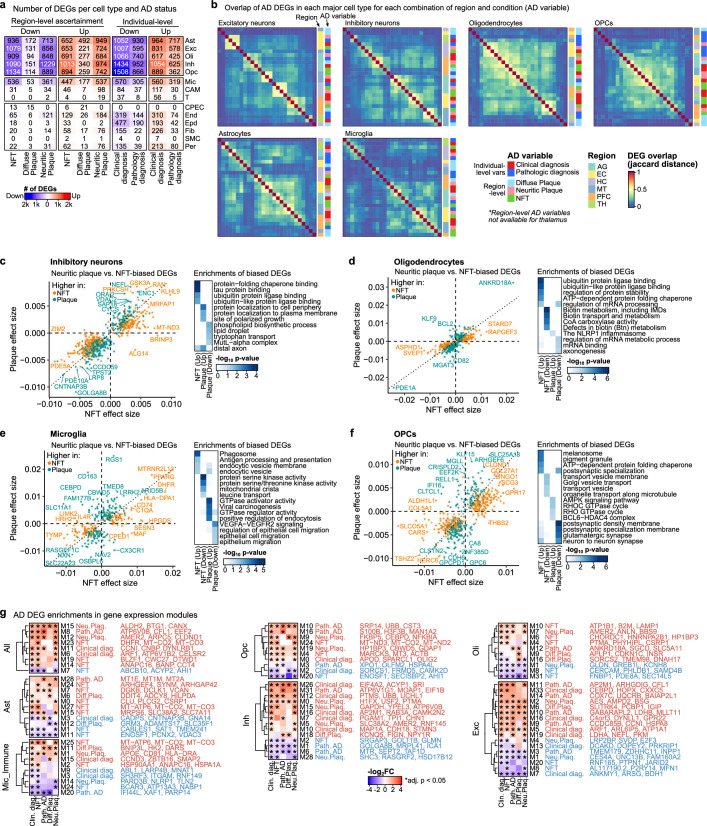
Pathology-biased DEGs for major cell types. **a**, Number of DEGs for each cell type for both region-level pathology measurements and individual-level AD status (DE analysis performed over all regions jointly). **b**, Overlap of AD DEGs in each major cell type for each combination of region and condition (AD variable). DEG overlap computed by Jaccard distance and rows/columns hierarchically ordered by Euclidean distance. **c**-**f**, Scatter plots of average effect sizes for NFT and plaque for DEGs with biased differential effect sizes (left panels) and their respective functional enrichments (right panels), for DEGs specific to inhibitory neurons (**c**), oligodendrocytes (**d**), microglia (**e**), and OPCs (**f**). Genes are coloured by whether they have higher effect size relative to NFT (orange) or plaque levels (teal). **g**, Heatmap of hypergeometric enrichments of up (red) or down (blue) AD DEGs in modules for DEGs in all sets of modules across all regions, by AD condition. Only modules with at least two significant enrichments are shown and rows are clustered hierarchically by Euclidean distance.

To determine whether GWAS-identified genes have regional associations with Alzheimer’s pathology, we intersected DEGs for regional pathology measurements with 149 identified familial AD and GWAS locus genes^56–58^ (Extended Data Figs. 10 and 11a). We found that 74 genes (49%) were differentially expressed for at least one cell type, and multiple genes showed region-specific expression, including the lipid transporter *ABCA7* (enriched in thalamus), the zinc-finger protein *ZNF655* (EC) and the complement receptor CR1 (neocortex)^56^ (Extended Data Fig. 10). GWAS and familial AD genes were maximally expressed (75 genes) and differentially expressed (30 genes) in microglia, and 25 genes were differentially expressed in at least three cell types, including upregulated *CLU*, *PLCG2* and *SORT1*, and downregulated *DENND6A* (Extended Data Fig. 10). Among all of the cell types, astrocytes and microglia showed the largest differential changes for these genes in regions with high neuritic plaque density, for example, for *APOE*, *HLA-DRA*, *PILRA* and *SORT1*, and showed the most response to diffuse plaque. Neurons and oligodendrocyte-lineage cells showed stronger differences for these genes, including for *PLCG2*, *CLU* and *MAF*, in regions with high NFT density (Extended Data Fig. 10).

## Pathology-specific expression changes

**Fig. 4:**
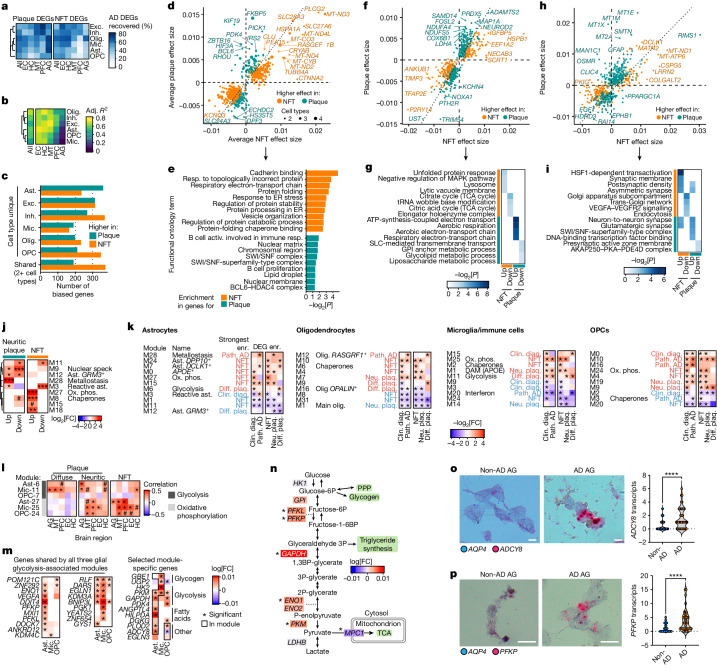
Gene expression modules annotate and separate AD changes across pathology. **a**, The percentage of AD DEGs (pathologic diagnosis) overlapping with DEGs for neuritic plaques (neu. plaq.) and NFTs in each major cell type and region. **b**, Concordance of effect-sizes between neuritic plaque and NFT DEGs. Adjusted *R*^2^ of log-transformed fold changes between neuritic plaque and NFT DEGs in each major cell type and region. **c**, The number of neuritic-plaque- or NFT-biased DEGs (≥3 DEGs for one of plaques or NFTs, and ≤2 for the other) for each major cell type or shared across 2+ cell types. **d**–**i**, The average effect sizes for NFTs and neuritic plaques for DEGs with biased differential effect sizes (**d**,**f**,**h**) and their respective functional enrichments (**e**,**g**,**i**), for DEGs shared across multiple cell types (**d**,**e**), in excitatory neurons (**f**,**g**) or in astrocytes (**h**,**i**). **j**, Enrichments (hypergeometric test) of pathology-biased DEGs in astrocyte modules. **k**, Enrichments (enr.) of AD DEGs in glial gene expression modules (*P_adj_ < 0.05, signed log_2_[fold change], only significant modules are shown). **l**, Pearson correlation of module scores in each region with region-level pathology measures for glycolysis and oxidative phosphorylation modules in astrocytes, microglia and OPCs (^#^*P* < 0.1). **m**, Core and selected diffuse plaque (diff. plaq.) DEGs in glial glycolysis-associated modules. **n**, Schematic of the glycolysis pathway, annotated by astrocyte diffuse plaque DEGs. Significant DEGs for diffuse plaques across all regions are indicated by asterisks. **o**,**p**, RNAscope validation of astrocyte energy metabolism DEGs in the AG of individuals with AD relative to control individuals without AD (pathologic diagnosis of AD). Representative images (left) show *AQP4* transcripts (blue puncta) and *ADCY8* (**o**) or *PFKP* (**p**) transcripts (red puncta). Scale bars, 20 μm (**o** and **p**). Quantification (right) was performed using unpaired two-tailed Student’s *t*-tests (*ADCY8*: *n* = 117 (non-AD) and *n* = 76 (AD) cells; *PFKP*: *n* = 43 (non-AD) and *n* = 40 (AD) cells). The dots represent individual cells, pooled from eight samples (four individuals; each had one PFC and one thalamus sample). Activ., activation; DAM, disease associated microglia; ox. phos., oxidative phosphorylation; resp., response.

To determine whether different pathologies induce distinct transcriptional responses, we computed DEGs for region-specific measurements of NFT and neuritic amyloid-β plaque burden (measured in each region except thalamus) (Fig. 4a, Extended Data Fig. 11a,b and Supplementary Fig. 41). DEGs for AD pathology showed a high overlap with DEGs for pathologic diagnosis (NFT: 45% and plaque: 53% on average) (Fig. 4a). Agreement between NFT and plaque DEGs was highest in the EC and HC for all cell types (average adjusted *R*^2^ of 67% in both) and lowest in the PFC (43%) and AG (21%), consistent with late-AD NFT appearance in the neocortex (Fig. 4b).

We next identified genes with higher differential effects in either NFTs or neuritic plaques (Fig. 4c, Extended Data Fig. 11c–f and Supplementary Table 10). Consistently, NFT-associated genes (374 genes, differentially expressed in 2+ cell types) included *PLCG2*, *CLU* and *CTNNA2* (in oligodendrocytes and OPCs) and mitochondrial subunits, and were enriched for ER protein processing, electron transport and cadherin binding (Fig. 4d,e). Neuritic-plaque-associated genes (190 genes) included the energy-homeostasis-regulating genes *IRS2*, *PDK4* and *HIF3A*, and genes enriched for immune response, chromatin regulation and lipid droplets. Notably, in excitatory neurons, plaque-associated and upregulated DEGs were strongly enriched for aerobic transport chain components (including *NDUFA4* and *COX6B1*) (Fig. 4f,g). On the other hand, NFT-associated and downregulated DEGs were enriched for TCA cycle genes, whereas upregulated DEGs were enriched for unfolded protein response and lysosome-linked genes. Finally, astrocytes contained more plaque-associated DEGs compared with other cell types, and their pathology-associated DEGs were enriched in our expression modules, including in metallostasis (M28) for plaque-associated DEGs and oxidative phosphorylation (M27) and chaperones (M8) for NFT-associated DEGs (Fig. 4c,h–j). Interestingly, a reactive astrocyte module (M3) was enriched in upregulated genes for plaques but in downregulated genes for NFTs (Fig. 4j).

Given the enrichment of NFT-associated or plaque-associated DEGs in expression modules, we next examined whether gene modules were enriched for AD DEGs (for AD pathology or for AD diagnosis) (Fig. 4k, Extended Data Fig. 11g and Supplementary Fig. 42). The same modules enriched for pathology-associated astrocyte DEGs were also enriched for the full sets of DEGs, including metallostasis (M28) with neuritic plaque DEGs and oxidative phosphorylation (M27) with NFT DEGs (Fig. 4k). Modules including ECM, adhesion and neurogenesis-related genes were much lower in AD (M1 and M11), while the modules for specific astrocyte subtypes (M7, *DCLK1*^+^; and M24, *DPP10*^+^) were enriched for upregulated DEGs (Fig. 4k).

We independently identified modules for heat-shock chaperones, glycolysis and oxidative phosphorylation in multiple cell types, which were correlated across cell types and were enriched for upregulated DEGs (Fig. 4k, Extended Data Figs. 6a,b and 11g and Supplementary Fig. 42a,b). The glycolysis modules were enriched among diffuse plaque DEGs in microglia and astrocytes and shared a set of genes that included canonical glycolysis genes (*PDK1/3/4*, *PFKL/P*, *PKM* and *PGK1*), anaerobic glycolysis enzymes (*TPI1* and *LDHA*) and stress-induced genes (*EGLN1*, *DDIT4*, *VEGFA* and *BNIP3L*) (Fig. 4k–m and Extended Data Fig. 6a,b). All glial types upregulated core glycolysis driver *GAPDH* and mitophagy-regulating *BNIP3L* in response to NFT burden and in individuals with cognitive impairment^59^ (Fig. 4m). In regions with high diffuse plaque, astrocytes upregulated glycolysis enzymes converting glucose-6-phosphate to pyruvate, while downregulating *MPC1*, the mitochondrial pyruvate transporter^60^ (Fig. 4n). In parallel, astrocytes uniquely upregulated *DDIT4*, *PFKP* and *ADCY8*, along with genes that suppress fatty acid metabolism (*ANGPTL4*) and promote lipid droplet storage of fatty acids (*HILPDA*), while microglia upregulated multiple glycogen-related genes (*GBE1*, *UGP2* and *PYGL*)^48^ (Fig. 4m).

To validate differential expression of *ADCY8* and *PFKP*, we performed in situ hybridization (RNAscope) in AG tissue samples from patients with AD and control individuals without AD and found a significant increase in transcripts of both genes in AQP4^+^ astrocytes (Fig. 4o,p). Finally, we noticed that the glycolysis pathway genes were maximally expressed at different points in global AD progression for each region (pathology diagnosis by ABC score)^29,30^. The pathway peaked very early in the EC (ABC score of 1, low levels of AD pathology), later in the HC and midtemporal cortex (intermediate levels), and very late in the PFC (high levels) (Supplementary Fig. 42c), suggesting that the glial metabolic response to AD may not be coordinated globally.

## Astrocytes and cognitive resilience

**Fig. 5:**
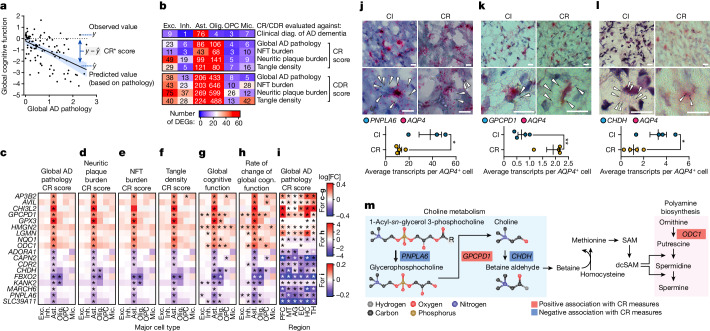
Molecular correlates of CR to AD pathology. **a**, The concept of CR and CDR scores. Pathology measurements are used to predict global cognitive function, for CR scores, or rate of cognitive decline, for CDR scores. **b**, The number of significant DEGs in major cell types across nine measures of CR. **c**–**h**, Association of astrocyte CR genes with measures of CR (global AD pathology CR score (**c**), neuritic plaque burden CR score (**d**), NFT burden CR score (**e**), tangle density CR score (**f**), global cognitive (cogn.) function (**g**) and rate of change of global cognitive function (**h**)) across six major cell types in the PFC (427 individuals, DEGs were computed using muscat). **i**, The association between the expression of CR genes in astrocytes across six brain regions and CR to global AD pathology (48 individuals; DEGs were computed using MAST). **j**–**l**, RNAscope validation of the differentially expressed astrocyte CR genes *PNPLA6* (**j**), *GPCPD1* (**k**) and *CHDH* (**l**) in the PFC of individuals with cognitive impairment (CI) relative to cognitively resilient (CR) individuals. Representative images (top) show *AQP4* transcripts (red puncta) and CR gene transcripts (blue puncta). Scale bars, 20 μm (**j**–**l**). Quantification (bottom) was performed using unpaired two-tailed Student’s *t*-tests; *P* = 0.0249 (**j**), *P* = 0.0052 (**k**), *P* = 0.0375 (**l**). Data are mean ± s.e.m. *PNPLA6*: *n* = 3 (CI) and *n* = 4 (CR) individuals; *GPCPD1* and *CHDH*: *n* = 4 individuals per group. **m**, Schematic of choline metabolism and polyamine biosynthesis; significant astrocyte CR genes are highlighted.

In addition to understanding cellular alterations associated with specific pathological measures in AD, we investigated what transcriptional changes are associated with cognitive resilience (CR) in AD, cases in which individuals with AD brain pathology display much less cognitive impairment than expected^3,25–27^. To identify potential molecular mediators that confer CR to AD pathology, we defined CR either categorically as the absence of cognitive impairment despite a pathologic diagnosis of AD (clinical diagnosis condition), or continuously, as the difference between observed cognition and the cognition expected on the basis of pathology level (Fig. 5a). We computed both scores for CR based on global cognitive function and for cognitive decline resilience (CDR) based on the rate of change of global cognitive function over time, and used four different measures of AD pathology: global AD pathology, neuritic plaque burden, NFT burden and tangle density (Fig. 5a).

We calculated DEGs for both CR and CDR in each major cell type in the PFC (snRNA-seq from 427 ROSMAP study participants)^27^. Astrocytes were the only cell type with a consistently high number of genes associated with CR across all of the measures tested (Fig. 5b). To identify specific molecular pathways within astrocytes that may contribute to CR, we focused on genes that are consistently associated with multiple measures of CR in astrocytes (termed CR-associated genes). Several CR-associated genes, including *GPX3*, *HMGN2*, *NQO1* and *ODC1* (encoding a rate-limiting enzyme of polyamine biosynthesis), possess or promote antioxidant activities^61–66^ (Fig. 5c–f and Supplementary Fig. 43a–d). The expression of *HMGN2*, *NQO1*, *ODC1* and *GPX3* in astrocytes was also positively associated with cognitive function (Fig. 5g and Supplementary Fig. 43e), and these genes exhibited the highest expression in astrocytes isolated from those individuals with the least cognitive decline over time (Fig. 5h and Supplementary Fig. 43f). Analysis of bulk RNA-seq data from the ROSMAP cohorts (*n* = 638) confirmed a significant positive association between the expression level of *HMGN2*, *ODC1* and *GPX3* and multiple measures of cognitive function and CR to AD pathology (Supplementary Fig. 44a–d).

Furthermore, we noticed that several CR-associated genes within astrocytes encode enzymes that catalyse metabolic reactions that are involved in choline formation and breakdown. The expression of *GPCPD1*, which encodes glycerophosphocholine phosphodiesterase 1, an enzyme that is critical for cleaving glycerophosphocholine (GPC) to produce choline, was positively associated with measures of CR in astrocytes (Fig. 5c–f and Supplementary Fig. 43a–d). Conversely, *PNPLA6*, which encodes a phospholipase that catalyses the hydrolysis of intracellular phosphatidylcholine, a major membrane lipid, generating GPC, and *CHDH*, which encodes choline dehydrogenase, an enzyme that catalyses the conversion of choline to betaine aldehyde, were both negatively associated with multiple measures of CR in astrocytes (Fig. 5c–f and Supplementary Fig. 43a–d). Many of the CR-associated genes identified in PFC astrocytes were also associated with CR in astrocytes from other regions of the human brain (Fig. 5i and Supplementary Fig. 45), corroborating a link between astrocytes and CR beyond the PFC.

To validate the choline pathway genes *PNPLA6*, *GPCPD1* and *CHDH*, we selected PFC samples from individuals with high amyloid and tau pathology and compared transcript levels between individuals with intact cognition (that is, cognitively resilient) to those with cognitive impairment, and performed in situ hybridization (RNAscope) analysis of these genes with *AQP4* as a marker for astrocytes. We found a significant decrease in *PNPLA6* and *CHDH* transcripts and a significant increase in *GPCPD1* transcripts in cognitively resilient individuals, in agreement with the differential expression results (Fig. 5j–l). Notably, choline oxidation to betaine generates a labile methyl group that can be used for homocysteine remethylation, resulting in methionine formation, which is subsequently transformed into the universal methyl donor *S*-adenosylmethionine^67^. *S*-adenosylmethionine is involved in the biosynthesis of spermidine, linking choline metabolism and polyamine biosynthesis in astrocytes in CR to AD pathology (Fig. 5m).

## Discussion

Here we present a transcriptomic atlas of the aged human brain—spanning six brain regions from 48 individuals with and without a diagnosis of AD—that we used to annotate regional cellular diversity, identify gene expression programs and differences in AD across cell types, and pinpoint region-specific cell populations that are vulnerable to AD. We provide an interactive website for exploring the atlas and these annotations, markers, functional modules and differences in AD at both the single-cell and pseudo-bulk levels (http://compbio.mit.edu/ad_multiregion).

By annotating neuronal and glial subtypes by brain region, we found significant compositional differences between regions, including a subtype of thalamic GABAergic neurons (*MEIS2*^*+*^*FOXP2*^*+*^) that is molecularly distinct from the canonical subclasses of inhibitory neurons in the neocortex. We used region-specific measurements of AD pathology to identify changes in gene expression associated with neurofibrillary tau tangle or amyloid-β plaque burden, including plaque-associated upregulation of metallostasis in astrocytes and of the electron-transport chain in excitatory neurons. We found that AD-risk genes were highly perturbed in AD—in particular for microglia, consistent with their enrichment for GWAS signal^68^—but few risk genes showed region-specific expression. To further examine the cellular and regional heterogeneity of the human brain, we developed a scalable method, scdemon, which uses sample decorrelation to annotate both ubiquitous and rare gene expression programs in each major cell type, and used annotated modules to identify functional programs associated with specific pathological variables, including a glycolysis- and energy-metabolism-linked program in glia^48,60^ associated with diffuse plaque burden.

We identified five excitatory neuron subtypes that were reduced in patients with AD (vulnerable subtypes) in the early-affected EC and HC^1,17,18^, including EC layer II (L2), *RORB*-positive L5 (*AGBL1*^+^*GPC5*^+^)^19^ and hippocampal CA1 subfield neurons^20–23^. Notably, vulnerable excitatory neurons shared expression of genes involved in Reelin signalling and heparan sulfate proteoglycan biosynthesis, both of which were also predictive of inhibitory neuron vulnerability to AD. Recent case studies have identified variants in *RELN* and *APOE* as potentially mediating CR to autosomal-dominant AD. Notably, the *RELN* variant enhanced its binding to glycosaminoglycans (GAGs) and *NRP1*, and the *APOE* variant decreased binding to GAGs, potentially affecting their ability to compete for receptor binding^69,70^. Thus, our findings suggest a convergence of factors associated with cellular vulnerability in sporadic AD, and resilience to autosomal-dominant AD.

Finally, we analysed the transcriptomic correlates of cognition and pathology in AD, and identified a set of astrocyte genes linked to CR to AD pathology. Notably, these genes converged on the pathways of choline metabolism and polyamine biosynthesis. This finding aligns with studies showing benefits of dietary choline intake and supplementation on cognitive performance in human individuals and in animal models^71–78^. Similarly, dietary supplementation with the polyamine spermidine prolongs life span and health span in several animal models^66^, and spermidine has also been shown to enhance memory performance and counteract age-related cognitive decline^79–81^. Our findings support choline metabolism and polyamine synthesis as attractive targets for promoting CR in AD.

Our study has several limitations: isotropic fractionation and read depth cut-offs may bias cell recovery based on their nuclear content; nuclear RNA may not fully capture microglial states^82^ or localized transcriptomic changes; and pathology burden is based on per-sample averages instead of on the spatial context of each cell. Additional individuals and data modalities will strengthen future analyses of region-specific alterations in AD, and spatial data may help to further separate pathology-associated changes.

## Methods

### Data reporting

No statistical methods were used to predetermine sample size. The experiments were not randomized and the investigators were not blinded to allocation during experiments and outcome assessment.

### snRNA-seq

#### Sample selection from ROSMAP

We selected 48 individuals from ROSMAP, both ongoing longitudinal clinical–pathological cohort studies of ageing and dementia, in which all of the participants are brain donors. The studies include clinical data collected annually, detailed post-mortem pathological evaluations, and extensive genetic, epigenomic, transcriptomic, proteomic and metabolomic bulk-tissue profiling^28^. For the purpose of this study, individuals were selected based on the modified NIA-Reagan diagnosis of AD and the Braak stage score (Braak stages 0, 1 and 2, *n* = 20; Braak stages 3 and 4, *n* = 14; Braak stages 5 and 6, *n* = 14), with 26 individuals having a positive pathologic diagnosis of AD and 22 individuals having a negative pathologic diagnosis of AD^83^. Details of the clinical and pathological data collection methods have been previously reported^2,5,6,28,84^. Individuals were balanced between sexes (male:female ratios 13:13 in AD, 11:11 in NoAD), matched for age (median, 86.6 years (AD) and 86.0 years (no AD)) and post-mortem interval (median, 5.9 h (AD) and 6.3 h (no AD)). Informed consent was obtained from each participant, and the Religious Orders Study and Rush Memory and Aging Project were each approved by an Institutional Review Board (IRB) of Rush University Medical Center. The participants also signed an Anatomic Gift Act, and a repository consent to allow their data to be repurposed.

#### Dissection criteria

All dissections were done on a bed of dry ice using either a fine-toothed razor saw (for cortical regions) or a jewellers saw with diamond wire (for subcortical regions). Region-specific descriptions are as follows. (1) AG: full thickness cortex from the AG (Brodmann area: BA 39/40); take from the first or second slab posterior to the end of the HC. Minimize white matter. (2) MT: full thickness cortex from the middle temporal gyrus (BA 22); take as close to the level of the anterior commissure as possible. Minimize white matter. (3) PFC: full thickness cortex from the frontal pole (BA 10); take from the lateral side of the first or second slab. Minimize white matter. (4) EC: full thickness cortex from the EC (BA 28); take at the level of the amygdala. Avoid amygdala. Minimize white matter. (5) Posterior HC: take from the last slab containing HC. If the last slab has less than 5 mm of HC, take from the next slab anterior. Collect a full cross section. (6) TH: take from the first slab with thalamus. Include the most medial aspect.

#### Isolation of nuclei from frozen post-mortem brain tissue

The protocol for the isolation of nuclei from frozen post-mortem brain tissue was adapted from a previous study^12^. All of the procedures were performed on ice or at 4 °C. In brief, post-mortem brain tissue was homogenized in 700 µl homogenization buffer (320 mM sucrose, 5 mM CaCl_2_, 3 mM Mg(CH_3_COO)_2_, 10 mM Tris HCl pH 7.8, 0.1 mM EDTA pH 8.0, 0.1% IGEPAL CA-630, 1 mM β-mercaptoethanol and 0.4 U µl^−1^ recombinant RNase inhibitor (Clontech)) using a Wheaton Dounce tissue grinder (15 strokes with the loose pestle). The homogenized tissue was then filtered through a 40 µm cell strainer, mixed with an equal volume of working solution (50% OptiPrep density gradient medium (Sigma-Aldrich), 5 mM CaCl_2_, 3 mM Mg(CH_3_COO)_2_, 10 mM Tris HCl pH 7.8, 0.1 mM EDTA pH 8.0 and 1 mM β-mercaptoethanol) and loaded on top of an OptiPrep density gradient (750 µl 30% OptiPrep solution (30% OptiPrep density gradient medium, 134 mM sucrose, 5 mM CaCl_2_, 3 mM Mg(CH_3_COO)_2_, 10 mM Tris HCl pH 7.8, 0.1 mM EDTA pH 8.0, 1 mM β-mercaptoethanol, 0.04% IGEPAL CA-630 and 0.17 U µl^−1^ recombinant RNase inhibitor)) on top of 300 µl 40% OptiPrep solution (40% OptiPrep density gradient medium, 96 mM sucrose, 5 mM CaCl_2_, 3 mM Mg(CH_3_COO)_2_, 10 mM Tris HCl pH 7.8, 0.1 mM EDTA pH 8.0, 1 mM β-mercaptoethanol, 0.03% IGEPAL CA-630 and 0.12 U µl^−1^ recombinant RNase inhibitor). The nuclei were separated by centrifugation (5 min, 10,000*g*, 4 °C). A total of 100 µl of nuclei was collected from the 30%/40% interphase and washed with 1 ml of PBS containing 0.04% BSA. The nuclei were centrifuged at 300*g* for 3 min (4 °C) and washed with 1 ml of PBS containing 0.04% BSA. The nuclei were then centrifuged at 300*g* for 3 min (4 °C) and resuspended in 100 µl PBS containing 0.04% BSA. The nuclei were counted and diluted to a concentration of 1,000 nuclei per μl in PBS containing 0.04% BSA.

#### Droplet-based snRNA-seq

For droplet-based snRNA-seq, libraries were prepared using the Chromium Single Cell 3′ Reagent Kits v3 according to the manufacturer’s protocol (10x Genomics). The generated snRNA-seq libraries were sequenced using NextSeq 500/550 High Output v2 kits (150 cycles) or NovaSeq 6000 S2 reagent kits.

### snRNA-seq processing, QC, and annotation

#### snRNA-seq data preprocessing

Gene counts were obtained by aligning reads to the GRCh38 genome using Cell Ranger software (v.3.0.2) (10x Genomics)^85^. To account for unspliced nuclear transcripts, reads mapping to pre-mRNA were counted. After quantification of pre-mRNA using the Cell Ranger count pipeline, the Cell Ranger aggr pipeline was used to aggregate all libraries (without equalizing the read depth between groups) to generate a gene–count matrix. The Cell Ranger v.3.0 default parameters were used to call cell barcodes. We used SCANPY^86^ to process and cluster the expression profiles and infer cell identities. We retained only protein-coding genes and filtered out cells with over 20% mitochondrial or 5% ribosomal RNA, leaving 1.47 million cells over 48 individuals and 283 samples across all regions. We further filtered the dataset to the top 5,000 most variable genes and used them to calculate the low dimensional embedding of the cells (UMAP) (default parameters, using 50 principal components and 15 nearest neighbours) and clusters using the Leiden clustering algorithm at a high resolution (15), giving 337 preliminary clusters^87^. We separately called doublets using DoubletFinder and flagged and removed clusters with strong doublet profiles and clusters showing strong individual-specific batch effects, leaving a final dataset of 1.35 million cells^88^.

#### Cell type annotations

For the UMAP visualization of individual major cell type classes (excitatory neurons, inhibitory neurons, astrocytes, oligodendrocytes, OPCs, immune cells), the SCTransform-based integration workflow of Seurat was used to align data from individual samples, using the default settings^89,90^. We selected the set of relevant principal components on the basis of Elbow plots. We annotated cell types using previously published marker genes and single-cell RNA-seq data^9,12,33,91–93^. To annotate cell types on the basis of previously published single-cell RNA-sequencing data (Allen Institute’s cell types database; https://portal.brain-map.org/atlases-and-data/rnaseq/human-multiple-cortical-areas-smart-seq), we used three separate approaches. First, Spearman rank correlation coefficients between the average expression profiles of neuronal subpopulations previously defined by the Allen Brain Institute^33^ and the neuronal subtypes identified in this study were computed using the cor function in R. Second, to project annotations of neuronal subpopulations previously defined by the Allen Brain Institute onto the neuronal cells analysed in this study, we followed the integration and label transfer workflow of Seurat^90^. Third, we determined cell type marker genes based on data published by the Allen Brain Institute^33^ using the FindAllMarkers function from Seurat (Wilcoxon rank-sum test with Bonferroni correction for multiple testing; *P*_adj_ < 0.05) and computed module scores for each cell type marker gene set across all neuronal cells analysed in this study using the AddModuleScore function of Seurat. To further annotate cell types, we determined marker genes using the FindAllMarkers function from Seurat (Wilcoxon rank-sum test with Bonferroni correction for multiple testing; *P*_adj_ < 0.05). We tested only genes that were detected in a minimum of 25% of the cells within the cell type (min.pct = 0.25) and that showed, on average, at least a 0.25-fold difference (log-scale) between the cells of the cell type and all remaining cells (logfc.threshold = 0.25). Marker genes of the high-resolution cell types or states were determined separately for each major cell type class. We additionally compared the EC excitatory neuron subtypes to cell type annotations previously reported previously^94^, which were computed using ACTIONet^95^, and compared microglial markers to previously reported subtypes^96,97^.

#### Cell cycle scores and global properties of gene expression

G2/M and S phase cell cycle scores were determined using the function CellCycleScoring in Seurat. Histograms showing the distribution of the G2/M- and the S phase module scores in each major cell class were generated using Prism 9 software. The statistical analyses comparing the number of genes detected per cell and the number of unique transcripts (UMIs) detected per cell between cell types was performed using Prism 9 software.

#### Integration of external data sources

Single-cell transcriptomic data from the human dLGN^98^ were obtained from the Allen Brain Institute (https://portal.brain-map.org/atlases-and-data/rnaseq/comparative-lgn). Single-cell transcriptomic data from multiple cortical areas and the hippocampal formation of the mouse brain^43^ were obtained from the Allen Brain Institute (https://portal.brain-map.org/atlases-and-data/rnaseq/mouse-whole-cortex-and-hippocampus-10x). Single-cell transcriptomic data across nine regions in the adult mouse brain^39^ were obtained from the McCarroll and Macosko Labs (http://dropviz.org/). Single-cell transcriptomic data from the mouse nervous system^40^ were obtained from the Linnarsson laboratory (http://mousebrain.org/adolescent/downloads.html). The external datasets and the human multiregion data presented in this study were integrated using the reciprocal PCA (RPCA) pipeline with the default parameters in Seurat (https://satijalab.org/seurat/articles/integration_rpca.html). The integration of single-cell data was performed separately for astrocytes, excitatory neurons and inhibitory neurons. For the integration of GABAergic neurons, the single-cell transcriptomic data from multiple cortical areas and the hippocampal formation of the mouse brain^43^ were downsampled to 50,000 GABAergic neurons. For the integration of excitatory neurons, the human multiregion dataset was downsampled to 5,000 neurons per high-resolution cell type. The mouse cortical data^43^ were downsampled to 50,000 excitatory neurons. The frontal cortex, posterior cortex, HC and thalamus data of the DropViz dataset were combined and downsampled to 50,000 neurons. Downsampling of the data was performed using the Seurat function subset with the default parameters. The comparison of microarray data from different human brain regions was performed using the Differential Search tool of the Allen Brain Atlas data portal (https://human.brain-map.org/microarray/search). The thalamus was selected as the target structure and compared to the cerebral cortex as the contrast structure. The differential search results including the fold change values and *P* values of the top 2,000 genes were downloaded from the data portal.

### Gene expression and regulon modules

#### Gene expression modules using ZCA (scdemon framework)

We would like to find gene expression modules by calculating gene–gene correlations in single-cell data and using these to detect communities of similarly expressed genes. However, in single-cell data, which often contain an unbalanced composition of cell types, modules computed using this approach will be dominated by the most common cell type markers and pathways. Moreover, correlation values will often be inflated for pairs of sparsely expressed genes. We developed a method which accounts for these pitfalls to call multiresolution gene expression programs from single-cell data using an SVD-based approximation of zero-phase component analysis (ZCA) and gene sparsity-dependent thresholding^99,100^.

#### scdemon (single-cell decorrelated module networks) method

The preprocessing transformations alternately called decorrelation, whitening, or sphering, transform a matrix *X* with a matrix *W* such that the covariance of *XW* is the identity matrix^101^. In particular, ZCA is the transformation which maximizes the similarity of the transformed data to the original, which is achieved by setting *W* = *C*^−1/2^, where *C* is the covariance of *X*.

In single-cell data, given a count matrix *X* with *n* cells (rows) and *g* genes (columns), we would like to perform ZCA decorrelation on the samples as a preprocessing step for calling modules. Computing and storing the *n* by *n* sample-wise covariance *C*_*n*_ = *XX*^*T*^/*g* is prohibitively expensive for modern datasets (with *n* > 1 × 10^6^), even without centring *X*. Instead, we can analytically approximate the covariance with the SVD of *X*^*T*^ = *U*_*n*_*S*_*n*_*V*_*n*_^*T*^ as *C*_*n*_ ≈ (*U*_*n*_*S*_*n*_*V*_*n*_^*T*^)^*T*^(*U*_*n*_*S*_*n*_*V*_*n*_^*T*^)/*g* = *V*_*n*_*S*_*n*_^2^*V*_*n*_^*T*^/*g* and therefore *C*_*n*_^−1/2^ = *g*^1/2^*V*_*n*_*S*_*n*_^−1^*V*_*n*_^*T*^. The ZCA transformation can then be computed as *X*_ZCA_ = *C*_*n*_^−1/2^*X* = *g*^1/2^*V*_*n*_*S*_*n*_^−1^*V*_*n*_^*T*^*X* before calculating the covariance of *X*_ZCA_ for downstream analysis. While this approximation is already tractable for small single-cell datasets, we may not be able to compute the matrix multiplications or centred SVD for larger datasets. Here, we can use the SVD of *X* = *USV*^*T*^, which is commonly calculated in single-cell analyses, to approximate *C*_*n*_^−1/2^ as *g*^1/2^*US*^−1^*U*^*T*^ and *X*_ZCA_ = *g*^1/2^*US*^−1^*U*^*T*^*X*. From this, the non-centred covariance of *X*_ZCA_ is *C*_ZCA_ = *X*_ZCA_^*T*^*X*_ZCA_/*n* = *g* × (*US*^−1^*U*^*T*^*X*)^*T*^(*US*^−1^*U*^*T*^*X*)/*n*. By substituting the SVD in for *X*, this reduces to *C*_ZCA_ = *g* × *VV*^*T*^/*n*, which is a simple and efficient approximation for very large single-cell data. As this approximation commonly drops out the largest identity program in the data due to the decorrelation approach, we allow computing *C*_ZCA_ = *g* × *VS*^*p*^*V*^*T*^/*n*, for any *p*, to tune the relative involvement of the larger eigenvalue components of the SVD.

To control for inflated correlation estimates in highly sparse genes, we bin the estimated correlations (*C*_ZCA_) for each pair of genes according to their sparsities (fraction of cells expressing the gene, binned on a log_10_ scale). We calculate the mean and s.d. for each 2D bin and smooth the estimates by fitting bivariate splines to the binned statistics, weighted by the log number of examples in the bin. We use the smoothed estimates to *z*-score the correlation matrix (*C*_ZCA_), which we then threshold with a single *z*-score cut-off to create an adjacency matrix for a gene–gene graph. Graphs are laid out using the Fruchterman–Reingold algorithm and we remove connected components with fewer than four genes^102^. We then use the leidenalg package and the Leiden algorithm with an RBConfiguration vertex partition to cluster the graph into gene modules^87^. To robustly estimate modules for each set of cells in our analyses, we first performed a grid search for the optimal number of SVD components for cells of that type. We then computed the *z*-scored matrices for each of 10 bootstraps, selecting 90% of batches for each bootstrap and using only genes expressed in over 5% of cells in the full dataset for the cell type. We thresholded the average of the bootstrapped *z*-score estimates with *z* = 4.5 to build a graph. To balance the contributions of modules across the compositional spectrum, we calculated and thresholded separate graphs for eigenvalue powers *p* = 0, 0.25, 0.5, 0.75 and 1 and combined them using multigraph Leiden clustering to call modules with leiden resolution = 3. Although we identify smaller modules, here we only report modules with at least 10 genes. We also ran the modules method on three published datasets, for which we ran the method with the same parameters on each dataset (*k* = 100, *z* = 4.5, resolution = 2.5), used genes with >5% sparsity for the COVID^103^ and brain^16^ datasets and genes with >10% sparsity for the Tabula Sapiens dataset^104^, and report modules with 10 genes or more.

#### Module enrichments, network and contour plots

Module enrichments for cell subtypes and brain regions were performed using the hypergeometric test by calculating whether cells with a module score above 1 s.d. from the mean score were significantly enriched in a specific subtype or region. For plotting scores against other modules, averaged modules scores to either the subtype by sample level (within the same major cell type) or at the sample level alone (across cell types) and calculated Pearson correlations and *P* values using the cor.test function of R. To create the module–module network across microglial and immune modules, we calculated module–module score Pearson correlations using the logged module scores at the aggregated subtype by sample level, using a one-sided test with *P*_adj_ < 0.01 as a cut-off^105^. To generate contour plots of module expression on a UMAP, we first smoothed cell-level expression on a 500 × 500 grid with a 2D Gaussian kernel (size = 25 × 25 and *σ* = 1) and then plot contours for smoothed values (0.1 to 0.8).

#### Gene expression programs using cNMF

Gene expression programs underlying both cell type identity and cellular activities were determined according to the consensus NMF (cNMF) analysis pipeline established previously using the default parameters^34^. The number of components (*K*) to use for cNMF was determined on the basis of a diagnostic plot showing the stability of the solution and the Frobenius reconstruction error as a function of *K*. To reduce runtime and working memory requirements, the data were downsampled using the Seurat function subset with the default parameters. The data were downsampled to 200 cells per major cell type. For the cNMF analysis at the level of high-resolution cell types, the analysis was performed separately on excitatory neurons, inhibitory neurons and astrocytes. For these analyses, the data were downsampled to 2,000 cells per astrocyte subtype and 1,000 cells per excitatory and inhibitory neuron subtype. Statistical significance of the overlap between the top 200 genes of a gene expression program and cell type marker genes was computed using Fisher’s exact tests.

#### SCENIC analysis and computation of regulon module scores

The gene regulatory network analysis was performed using pySCENIC with the default parameters^35^. To reduce runtime and working memory requirements, the data were downsampled to 2,000 cells per major cell type. For the SCENIC analysis at the level of high-resolution cell types, the analysis was performed separately on excitatory neurons, inhibitory neurons and astrocytes and the data were downsampled to 1,000 cells per high-resolution cell type. To identify the top cell-type-specific regulons, we calculated regulon specificity scores as described by previously and ranked the regulons based on their regulon specificity score^106^. Finally, we calculated the activity of each regulon in each cell using the AddModuleScore function of Seurat. The calculation of regulon module scores for major cell types was performed on a random sample of 50% of the cells (676,537 cells). For the analysis at the level of high-resolution cell types, the regulon module scores were determined based on all the cells of a major cell type class. For the statistical analysis of differences in the activity of regulons between cell types, the average regulon module score per individual and major cell type or high-resolution cell type was computed, respectively. The statistical analyses comparing the regulon module score activity was performed using Prism 9 software.

#### Analysis of GABAergic and glutamatergic module scores

GABAergic and glutamatergic module scores across all neuronal cell types were determined on the basis of a set of GABAergic and glutamatergic neuron marker genes, respectively, using the AddModuleScore function of Seurat. The sets of GABAergic and glutamatergic neuron marker genes were determined based on the human multiple cortical areas SMART-seq dataset from the Allen Brain Institute (https://portal.brain-map.org/atlases-and-data/rnaseq/human-multiple-cortical-areas-smart-seq). We identified marker genes using the FindAllMarkers function from Seurat (Wilcoxon rank-sum test with Bonferroni correction for multiple testing; *P*_adj_ < 0.05). We tested only genes that were detected in a minimum of 25% of the cells within the cell type (min.pct = 0.25) and that showed, on average, at least a 0.25-fold difference (log-scale) between the cells of the cell class of interest and all remaining cells (logfc.threshold = 0.25). To quantify the intermediate character of thalamic excitatory and inhibitory neurons, we first computed the average GABAergic and glutamatergic module score values for each neuronal cell type and for each individual. We then used the resulting data to determine the first principal component (PC1) scores (the coordinates of the individual observations on the first principal component axis) using the princomp function in R. The ridgeline plot showing the distribution of PC1 score for each neuronal cell type was generated using the ggplot2 package in R. To determine the association between the average glutamatergic and the GABAergic module score across neuronal cell types, we performed a simple linear regression analysis using Prism 9 software.

#### Analysis of extratelencephalic projection neuron module scores

Marker genes significantly upregulated in extratelencephalic projection neurons (exc. L5 ET) compared with near-projecting excitatory neurons in layers 5 and 6 (exc. L5/6 NP) were determined using the FindAllMarkers function from Seurat (Wilcoxon rank-sum test with Bonferroni correction for multiple testing; *P*_adj_ < 0.05). We tested only genes that were detected in a minimum of 25% of the cells within the cell type (min.pct = 0.25) and that showed, on average, at least a 0.25-fold difference (log-scale) between exc. L5 ET cells and exc. L5/6 NP cells (logfc.threshold = 0.25). The exc. L5 ET module score was computed based on the identified marker genes using the AddModuleScore function of Seurat. To determine the exc. L5 ET module scores across excitatory and inhibitory neurons, cells were downsampled to 2,000 cells per high-resolution cell type.

### Cell–cell communication analysis

Cell–cell communication events were predicted using the LIgand-receptor ANalysis frAmework (LIANA)^107^ in R. Specifically, the ligand–receptor analysis was performed using liana_wrap(). The methods included were CellPhoneDB^108^, NATMI^109^ and SingleCellSignalR^110^. liana_aggregate() with the argument ‘aggregate_how’ set to ‘magnitude’ was run to find consensus ranks of different methods. Only interactions (ligand–receptor pairs) with a robust rank aggregation (RRA) score smaller than 0.05 (aggregate_rank < 0.05) were considered in downstream analyses. The interaction score of ligand–receptor pairs was calculated by applying −log_10_ transformation to the RRA score (aggregate_rank). To determine the number of interactions and the overlap of interactions between regions, liana_wrap() was run on the pool of cells isolated from all individuals, with separate analyses conducted for each brain region. To determine cell–cell communication events that are brain-region specific, liana_wrap() was run separately for each individual. We then used a linear mixed-effects model to evaluate the association between the interaction scores of individual ligand–receptor pairs obtained from LIANA and the respective brain region serving as the predictor variable. To account for potential confounding factors and individual variability, we included age, sex and post-mortem interval as covariates in the linear mixed-effects model. These variables were added as fixed effects to the model. Moreover, we included a random effect for the individual to capture the participant-specific variability in the data. Linear mixed-effects models were implemented using the R software packages lme4^111^ and lmerTest^112^. The lmer() function from the lme4 package was employed to fit the models. To obtain *P* values for the fixed effects in these models, we used the lmerTest package, which incorporated Satterthwaite’s degrees of freedom approximation. To account for multiple hypothesis testing, the obtained *P* values were further adjusted using the Bonferroni method.

### Cell type composition

#### Analysis of cell type composition differences between brain regions

For comparing the relative abundance of major cell types across brain regions, the fraction of cells of a major cell type class was computed relative to all the cells isolated from a region. For the statistical analysis of cell type composition differences between brain regions, we also computed the relative abundance of major cell type classes separately for each study participant. To this end, the fraction of cells of a major cell type class was computed relative to all the cells isolated from a brain region of an individual. At the level of high-resolution cell types or cell states, two distinct measures of relative abundance were computed. First, the relative abundance of each subtype of a major cell class was computed as the proportion of a subtype relative to all cells of the corresponding major cell class isolated from a brain region. Second, for the statistical analysis of differences between brain regions, the fraction of cells of a subtype was computed relative to all the cells isolated from a brain region of an individual. The statistical analyses comparing the relative abundance of major cell types and subtypes between brain regions was performed using Prism 9 software.

#### Analysis of cell type composition alterations in AD

We calculated compositional differences in individuals with AD versus individuals without AD (or AD dementia versus no dementia) by modelling the number of cells of a certain cell type or subtype in a specific sample (individual by region) relative to the total number of cells using a quasi-binomial regression model. We modelled AD status by binary ascertainment variables (cogdx 4–5, NIA-Reagan score 1–2, Braak stage 5–6 versus others, as well as any detected presence of NFTs, neuritic plaque or diffuse plaques in the region) while adjusting for brain region and sex. We used the emmeans package in R to assess the significance of the regression contrasts and used p.adjust with the fdr method to adjust *P* values. We modelled the effects of fraction of cells on cognitive performance in multiple domains with gaussian linear regression of cognitive performance on last visit versus the log_10_-transformed fraction of cells in the subtype or major cell type jointly with covariates for age, sex, APOE-ε4 and post-mortem interval, with false-discovery rate *P*-value correction (p.adjust in R). We compared the fractional abundances of pairs of neuronal subtypes between two regions using Kendall’s *τ* only in individuals with AD (NIA-Reagan score). Significance was assessed using beta regression (R library betareg) controlling for sex, *APOE* genotype, post-mortem interval and age of death, and we adjusted *P* values using p.adjust in R with the fdr method.

### Differential gene expression

#### DEGs in AD

We performed differential expression analyses with three separate methods: MAST, Nebula and Wilcoxon testing^113,114^. For all methods, we subset the tested genes to only genes present in over 20% of cells. For MAST and Nebula, we calculated and included in the regression the top 10 components of unwanted variation calculated using RUV on the pseudo-bulk-level data (individuals by regions). For these methods, we also included as covariates the individual’s sex, age of death and post-mortem interval, each cell’s counts per gene and number of captured genes and, where applicable, the high-resolution cell subtypes and the brain region. For Nebula we used a Poisson mixed-model on the counts data with an offset of the log_10_-transformed total counts per cell. For MAST and Wilcoxon, we normalized each cell to a total library size of 10,000 counts. We ran Wilcoxon tests on both the cell and individual levels. We adjusted *P* values for multiple testing in all cases by using the p.adjust function in R with the fdr method. For our final set of differential genes in each analysis, we took all genes that were significant (*P*_adj_ > 0.05) and concordant in both the MAST and Nebula results. We also provide the results for Wilcoxon tests, but did not use these to determine concordant results as they do not correct for many covariates. We computed differential expression against five AD ascertainment variables: continuous measurements of NFT, plaq_n, and plaq_d measured in each region except the thalamus (excluded from these analyses) and binary cognitive impairment (cogdx no dementia = 1 and 2 versus AD dementia = 4 and 5) and NIA-Reagan score classifications (non-AD = 3 and 4 versus AD = 1 and 2). We provide differential expression results for each of the 14 major cell types (with T cells, CNS macrophages, and each vascular subtype separately) for all regions jointly and for each region separately. We also provide results for each of the excitatory subtypes either in its most prevalent region for EC, HC or TH subclasses, or across the neocortex for neocortical subtypes (Supplementary Table 9). We also computed DEGs for the interaction between pathologic diagnosis of AD and sex in each major cell type, both across all regions and in each region separately. For the glial energy metabolism analyses we recomputed all DEGs in glial glycolysis-associated modules separately (keeping all genes, with no cell percentage cut-off). Glycolysis pathway diagram is from the glycolysis and gluconeogenesis pathway from WikiPathways^115^.

#### Pathology-biased DEGs

Pathology-biased DEGs were based on neuritic plaque or NFT pathology measurements in each region and were computed in each major cell type across all regions and in each region (except for the TH). Genes were ordered by the residual between NFT effect size and predicted NFT effect size from a regression using plaque effect size and region. Genes were retained if they were consistently up (or down) in 3+ regions for either NFT or plaque but in fewer than 2 regions for the other pathology measurement (shared genes are genes found in 2+ cell types).

#### Comparison with published DEGs

We compared our differential expression results to results from seven different previously published studies^11,12,19,47–50^. We compared the published DEGs both to: (1) cross-region DEGs calculated in each major cell type for individual-level AD status (NIA-Reagan score or clinical diagnosis of AD) and for quantitative measurements of AD pathology (neuritic plaques, diffuse plaques and NFTs); and (2) region-specific DEGs calculated in each major cell type and in endothelial cells, computed relative to pathologic diagnosis of AD (NIA-Reagan score, AD, 1–2; non-AD, 3–4). As some studies reported only the significant genes, we compared the log-transformed fold change estimates for our DEGs and reported DEGs by a Pearson correlation test.

#### DEG module enrichments

To assess the enrichment of upregulated, downregulated non-differentially expressed genes in each module, we first assigned each tested DEGs to its closest module by correlation to the module’s average expression profile. We then performed a hypergeometric enrichment test for the number of genes in a category (upregulated, downregulated, not differentially expressed) assigned to the module, against the total number of tested genes assigned to the module, the total number in the category and the total number tested and corrected *P* values using p.adjust (Benjamini–Hochberg). Enrichments of pathology-biased DEGs in modules were performed in the same manner.

#### Neuronal DEG partitions

To partition neuronal DEGs into non-vulnerable and vulnerable-associated subclasses, we calculated each genes’ average expressions and differential expression effect sizes at the subtype level and compared these to the relative depletion of the subtypes. For each gene that was differentially expressed in late-AD (stratified by Braak stage, late AD, 5–6 versus non-AD or early-AD, 1–4) in at least 25% of all neuronal subtypes, we calculated the correlation of its average subtype expression in late-AD with each subtype’s compositional stability (log_2_[OR] in late AD) across excitatory subtypes, separating non-vulnerable-associated genes (correlation > 0.2) from vulnerable-associated genes (correlation < −0.2). We calculated functional enrichments on neuronal DEG partitions using the top 250 genes ordered by effect size in each category. We further separated DEGs with higher effect sizes in vulnerable subtypes from those with similar effect sizes across all neuronal subtypes by calculating the correlation of their differential effect sizes in each subtype with that subtype’s depletion (log_2_[OR] in late AD). To perform enrichments along the continuum of genes associated with vulnerability to non-vulnerability, we kept only genes with biased effect sizes (effect size correlation < −0.2) and binned them along the axis of expression correlation (window size 0.2 for at 0.05 intervals) and performed functional enrichments for all bins jointly.

#### DEG and module pathway enrichments

We performed DEG enrichments for each differential expression run using the gprofiler2 package in R, with multi-query for upregulated and downregulated genes, as unordered queries, a *P*-value cutoff of 0.05, and using GO, REAC, WP, KEGG and CORUM as annotation sources, and retained enriched terms with fewer than 500 genes. Module and module cluster enrichments were performed in the same manner, using the core genes identified for each module and for genes found in more than two modules within a module cluster.

#### Markers of neuronal vulnerability

We identified markers associated with excitatory neuron subtype vulnerability by performing linear regression to predict the log_2_[OR] of each subtype’s depletion in late AD based on its expression at the subtype-aggregate level (log[*X* + 1], averaged normalized expression in each subtype by region by individual batch), controlling for age, sex and post-mortem interval and adjusting *P* values with p.adjust (fdr).

#### Identification of genes associated with cellular vulnerability in inhibitory neurons

Processed snRNA-seq data (DLPFC, experiment 2) were obtained from Synapse (syn51123521) and integrated with our own PFC snRNA-seq dataset comprising 427 individuals. To identify vulnerable inhibitory neuron subtypes, we examined the association between the relative abundance of cell types and the measure of NFT density (variable tangles). We used a quasi-binomial regression model to model the number of cells belonging to a specific cell type in a given sample (individual study participant), relative to the total number of cells in that sample. We fitted the regression model using the glm function in R, including age, sex and post-mortem interval as covariates. *P* values were corrected for multiple testing using the Benjamini–Hochberg procedure as implemented in the R function p.adjust. The results are presented in the form of association scores (signed −log_10_-transformed Benjamini–Hochberg-adjusted *P* value, where the sign was determined by the direction (positive or negative) of the association). Inhibitory neuron subtypes demonstrating a significant negative association with tangle density (Benjamini–Hochberg-adjusted *P* value < 0.05) were classified as vulnerable subtypes, while all other subtypes were categorized as non-vulnerable. Genes exhibiting differential expression between vulnerable and non-vulnerable inhibitory neuron subtypes were identified on the basis of our PFC snRNA-seq dataset. This analysis was restricted to individuals without a pathologic diagnosis of AD. The differential expression analysis comparing vulnerable to non-vulnerable inhibitory neuron subtypes was performed using the R package dreamlet (https://diseaseneurogenomics.github.io/dreamlet/). We used the dreamletCompareClusters function with the argument ‘method’ set to ‘fixed’ for this analysis. Adjusted *P* values for multiple testing were obtained using the topTable function of dreamlet, with the ‘adjust.method’ set to ‘BH’.

#### GWAS analyses

Intersection of regional expression and pathology-specific DEGs (across all regions) was performed for 149 annotated AD GWAS familial and AD risk loci from recent GWAS^54,56–58^. We calculated the disease-relevance score of each cell in the dataset against a recent Alzheimer’s GWAS, using scDRS (based on MAGMA)^54,55,116^. For the scDRS results, we counted the fraction of cells with significant scDRS scores (FDR < 0.05) in each cell type, subtype and region. To test for overlap with microglia/immune modules, we compared the set of immune cells with significant expression of each module (*z* score > 2.5) and with the set of cells with significant scDRS scores (FDR < 0.05) and performed a hypergeometric test for significance of the overlap (*P*_adj_ < 0.01, Benjamini–Hochberg correction). To identify region-specific GWAS genes, we performed an analysis of variance for the effect of region on average gene expression at the pseudobulk level.

#### Identification of genes associated with CR

To quantify CR, we computed a CR score as the difference between the observed cognition and the cognition predicted by a linear regression model, given the level of pathology (Fig. 5a). Using this approach, we computed cognitive resilience (CR) scores based on the measure of global cognitive function and CDR scores based on the measure estimating the rate of change of global cognitive function over time (Fig. 5a). Four distinct CR and CDR scores were derived using four distinct measures of AD pathology, namely global AD pathology and, separately, neuritic plaque burden, NFT burden and tangle density.

We performed differential expression analyses using the R package muscat to identify genes associated with CR in the PFC^117^. Low-expressed genes were excluded and only genes with more than one count in at least ten cells were considered. To take advantage of robust bulk RNA-seq differential expression frameworks, such as edgeR^118^, in a first step, muscat aggregates measurements for each sample (in each cluster) to obtain pseudobulk data. Using this approach, single-cell measurements were aggregated per study participant and cell type using the sum of raw counts option. Differential expression analysis was run using the edgeR method as implemented in muscat. We included as covariates the individual’s age at death and post-mortem interval. We report adjusted *P* values for multiple testing in all cases by using the p.adjust function with the Benjamini–Hochberg method as implemented in muscat. The multiple testing correction was performed locally, that is, on each of the cell types separately with the number of tests equal to the number of genes considered. These differential expression analyses were performed on the entire set of 427 individuals except for the group-based differential expression analysis based on our categorical definition of CR. In this case, we focused on comparing two distinct groups determined by their pathologic and clinical diagnoses of AD. First, we identified individuals with a pathologic diagnosis of AD, using the NIA-Reagan pathology criteria. Subsequently, these individuals were further categorized on the basis of their clinical consensus diagnosis of cognitive status at the time of death. Specifically, we compared individuals with no cognitive impairment (NCI, final consensus cognitive diagnosis (cogdx) value of 1) against individuals with a cognitive diagnosis of AD dementia and no other cause of cognitive impairment (cogdx value of 4) among individuals with a pathologic diagnosis of AD.

To confirm the differential gene expression results based on the CR and CDR scores, we also evaluated the association between gene expression and global cognitive function or the rate of change of global cognitive function adjusting for AD pathology as a covariate. The AD pathology measures considered as a covariate were global AD pathology (gpath), neuritic plaque burden (plaq_n), NFT burden (nft), or tangle density (tangles). Thus, together with the DGE analysis based on CR and CDR scores, we performed a total of 16 different tests assessing the association between gene expression and CR.

We used the model-based analysis of single-cell transcriptomics (MAST) tool to investigate whether the CR genes identified in PFC astrocytes were also associated with CR in astrocytes from other regions of the human brain. To ensure robust analysis, we initially filtered the genes under investigation, selecting only those with more than one count in at least 10 cells. The analytical model incorporated the condition variable of interest, as well as several covariates known to influence gene expression. These covariates included the cellular detection rate (cngeneson), age at death (age_death), post-mortem interval (pmi), and sex (msex). We also accounted for potential participant-specific variation in the data by incorporating a random effect term for the individual (1|individual). To account for multiple comparisons, the *P* values were adjusted using the FDR method as implemented in the p.adjust function.

#### Bulk RNA-seq differential expression analysis

Differential expression analysis of bulk RNA-seq data from the ROSMAP cohorts was performed using DESeq2^119^ (plotted) and edgeR^118^. Age at death and post-mortem interval were converted into *z* scores and included as covariates in the regression equation. Both approaches (DESeq2 and edgeR) provided similar results.

#### Permutation test for evaluating the significance of the overlap of DEGs between our dataset and the SEA-AD dataset

The average expression level of each gene within each major cell type was determined using the ‘AverageExpression’ function from the Seurat R package. The genes considered in the differential expression analysis for each major cell type were categorized into ten subsets based on their average expression level within the corresponding cell type. We next intersected the genes in each of the ten subsets with genes identified as significantly associated with either neuritic plaque burden (in our dataset) or the CPS score (in the MTG SEA-AD dataset). This intersection enabled us to determine the number of significant DEGs in each subset. The process was performed separately for genes positively and negatively associated with AD pathology. Subsequently, we randomly sampled the determined number of significant DEGs from each of the 10 subsets, ensuring that the expression level distribution of the DEGs was preserved in the random samples. This random sampling step was repeated for a total of 1,000 iterations. These steps were performed separately for both our dataset and the SEA-AD dataset. For each of the 1,000 random samples, we determined the overlap of genes between datasets and compared it to the observed overlap between the two datasets. To assess the significance of the observed overlap, we computed *z* scores, which represent the difference between the observed value of overlap and the mean value of overlap based on the permutation results, divided by the s.d. of the permutation results.

#### Comparison with previously published proteomics study of AD

To further validate our differential expression results, we evaluated the correlation between the effect sizes of gene expression changes observed in our study and those identified through quantitative proteomics^51^. We specifically examined the correlation between the effect sizes of genes associated with neuritic plaque burden in our study and the effect sizes of overlapping differentially expressed proteins in the quantitative proteomics analysis of bulk tissue. The correlation was computed using the cor.test function in R with the argument ‘alternative’ set to ‘two.sided’ and the argument ‘method’ set to ‘pearson’. *P* values were adjusted for multiple testing using the Benjamini–Hochberg method as implemented in the R function p.adjust.

#### Inter-regional comparison of AD pathology-associated gene sets in glial cells along the spectrum of global AD pathology burden

We determined genes significantly associated with global AD pathology for each glial cell type, using single-nucleus RNA sequencing data from the PFC of 427 participants in the ROSMAP study. We then calculated module scores for these gene sets in astrocytes, microglia, oligodendrocytes, and OPCs using the Seurat ‘AddModuleScore’ function. The module scores were determined separately for genes positively and negatively associated with global AD pathology. To assess the progression of these scores across the spectrum of global AD pathology burden, we averaged the module scores of all cells of a specified cell type isolated from the brain region of interest of an individual. For visualizing the relationship between the global AD pathology burden and mean module scores, we employed Locally Estimated Scatterplot Smoothing (LOESS) using the ggplot2 package in R, with the ‘geom_smooth’ function and the ‘method’ parameter set to ‘loess’. The correlation of mean module scores between regions was determined using the cor.mtest function of the R package corrplot. *P* values were adjusted for multiple hypotheses testing using the Benjamini–Hochberg method as implemented in the R function p.adjust.

### In situ hybridization (RNAscope)

Frozen human post-mortem brain samples were embedded in Tissue-Tek OCT compound (VWR; 25608-930), sectioned on a Leica cryostat at a thickness of 20 μm, and mounted onto Fisherbrand Superfrost Plus microscope slides (Thermo Fisher Scientific; 12-550-15). Slides were fixed in 4% paraformaldehyde at 4 °C for 30 min, and dehydrated in ethanol. The RNAscope 2.5 HD Chromogenic Duplex Detection Kit and RNAscope Multiplex fluorescent V2 Kit (ACDBio) were then used according to the manufacturer’s instructions. Tissue samples were hybridized using the following chromogenic RNAscope probes: GAD2, FOXP2, MEIS2, AQP4, GRM3, ADCY8, PFKP, PNPLA6, GPCPD1 and CHDH (ACDBio). For in situ hybridization of Reelin, tissue samples were hybridized using the following fluorescent RNAscope probes: vGlut and Reelin. Cell nuclei were stained with 50% haematoxylin (for chromogenic experiments) or with Hoechst (for fluorescent experiments). For fluorescence RNAscope analysis, sections were incubated in TrueBlack (Biotium; 23007) for 10 s before Hoechst staining to quench auto-fluorescence. Images were acquired using the Zeiss LSM 900 confocal microscope, with a 63× oil objective. Two images were acquired per tissue sample. For both chromogenic and fluorescence RNAscope experiments, puncta were manually counted by researchers blinded to the experimental group of each image.

### Immunohistochemistry

All experiments were performed according to the Guide for the Care and Use of Laboratory Animals and were approved by the National Institute of Health and the Committee on Animal Care at Massachusetts Institute of Technology. Sample sizes were determined on the basis of previous work from our laboratory, without power analysis calculation or randomization. In the experiment comparing *App*-KI (C57BL/6-App<tm3(NL-G-F)Tcs>, RBRC06344) and WT mice, the *App*-KI group consisted of 7 mice (5 male and 2 female) and the WT group included 6 female mice. In the experiment comparing Tau P301S (The Jackson Laboratory, 008169) to WT mice, both groups consisted entirely of male mice, with 6 mice in the Tau(P301S) group and 5 mice in the WT group. Mice were transcardially perfused with ice-cold phosphate-buffered saline, followed by 4% paraformaldehyde for fixation. Brains were dissected out and post-fixed in 4% paraformaldehyde overnight at 4 °C. Brains were sectioned horizontally on the Leica vibratome at a thickness of 40 μm. Slices containing the EC were selected under a dissecting microscope to ensure consistent anatomical structure across all cohorts. Brain sections were incubated in antigen retrieval solution (pH 6, 100 mM sodium citrate buffer, prewarmed to 80 °C) for 20 min, and then cooled to room temperature. The sections were then washed twice with phosphate-buffered saline, and blocked in buffer (0.3% Triton X-100, 2% bovine serum albumin, 10% normal donkey serum in phosphate-buffered saline) for 1 h at room temperature.

The sections were incubated in primary antibodies (anti-Reelin, 1:200; anti-NeuN, 1:200, anti-phospho tau, 1:200; and anti-amyloid-β, 1:500) overnight at 4 °C. After primary antibody incubation, the sections were washed three times with PBS, twice with blocking buffer and incubated in secondary antibody (1:1,000) for 2 h at room temperature. The sections were then washed three times with PBS, incubated with Hoechst (1:1,000) for 10 min and washed once more with PBS.

Confocal tile scans of the EC were acquired on the Zeiss LSM 900 using a 20× objective, with consistent laser setting across all cohorts. Layer II–III EC was identified based on previous criteria^120^. Orthogonal projections of the confocal tile scans were exported to Fiji for signal quantification. In Fiji, layer II–III of the EC was set as a region of interest, and a macro was used to count Reelin-positive cells in the region of interest and quantify the mean fluorescence intensity for each cell. The signal intensity of the Reelin channel was subsequently normalized to the NeuN signal. Researchers were blinded to animal genotype.

### External data sources

Processed snRNA-seq data generated by the Seattle Alzheimer’s Disease Brain Cell Atlas (SEA-AD) consortium (SEAAD_MTG_RNAseq_final-nuclei.2023-05-05.h5ad) were obtained from the Seattle Alzheimer’s Disease Brain Cell Atlas (SEA-AD) (https://sea-ad-single-cell-profiling.s3.amazonaws.com/index.html#MTG/RNAseq/). The SEA-AD DLPFC data (SEAAD_DLPFC_RNAseq_final-nuclei.2023-07-19.h5ad) were downloaded from https://sea-ad-single-cell-profiling.s3.amazonaws.com/index.html#DLPFC/RNAseq/. Additional processed snRNA-seq datasets (specifically the h5ad files Neurons.h5ad and Nonneurons.h5ad) were obtained from the Linnarsson laboratory (https://console.cloud.google.com/storage/browser/linnarsson-lab-human;tab=objects?authuser=0&prefix=&forceOnObjectsSortingFiltering=false).

### Reporting summary

Further information on research design is available in the Nature Portfolio Reporting Summary linked to this article.

## Online content

Any methods, additional references, Nature Portfolio reporting summaries, source data, extended data, supplementary information, acknowledgements, peer review information; details of author contributions and competing interests; and statements of data and code availability are available at 10.1038/s41586-024-07606-7.

### Supplementary information

Supplementary Information Supplementary Figs 1–45.

Reporting Summary

Supplementary Tables Supplementary Tables 1–10 and a Supplementary Table guide.

### Source data

Source Data Fig. 3

## Data Availability

snRNA-seq profiling data are available from Synapse in coordination with the ROSMAP project. Data are accessible under accession codes syn52293442 (as part of the MIT ROSMAP Single-Nucleus Multiomics Study; Synapse: syn52293417). The data are available under controlled use conditions set by human privacy regulations. To access the data, a data use agreement is needed. This registration is in place solely to ensure anonymity of the ROSMAP study participants. A data use agreement can be agreed with either Rush University Medical Center (RUMC) or with SAGE, who maintains Synapse, and can be downloaded from their websites (https://www.radc.rush.edu/; https://adknowledgeportal.synapse.org/). Additional processed data as well as integrative visualization and exploration of the atlas are available online (http://compbio.mit.edu/ad_multiregion/ and https://ad-multi-region.cells.ucsc.edu/)^121^. We also downloaded the following public single-cell gene expression datasets: human multiple cortical areas SMART-seq (https://portal.brain-map.org/atlases-and-data/rnaseq/human-multiple-cortical-areas-smart-seq), human DLPFC (Synapse: syn51123521), SEA-AD MTG (https://sea-ad-single-cell-profiling.s3.amazonaws.com/index.html#MTG/RNAseq/), SEA-AD DLPFC (https://sea-ad-single-cell-profiling.s3.amazonaws.com/index.html#DLPFC/RNAseq/), human dLGN (https://portal.brain-map.org/atlases-and-data/rnaseq/comparative-lgn), multiple human brain regions (https://console.cloud.google.com/storage/browser/linnarsson-lab-human;tab=objects?authuser=0&prefix=&forceOnObjectsSortingFiltering=false), multiple cortical areas and the hippocampal formation of the mouse brain (https://portal.brain-map.org/atlases-and-data/rnaseq/mouse-whole-cortex-and-hippocampus-10x), nine regions in the adult mouse brain (http://dropviz.org/) and Mouse Brain Atlas (http://mousebrain.org/). Source data are provided with this paper.

## Code availability

Code for analysis is available at GitHub (https://github.com/cboix/admultiregion_analysis) and Zenodo (10.5281/zenodo.11051020). The code for the scdemon method for module detection from single-cell RNA-seq is available at GitHub (https://github.com/KellisLab/scdemon).
