# Supplementary material for: Single-cell multiregion dissection of Alzheimer’s disease
Source: Nature. 2024 Jul 24;632(8026):858–68. doi: 10.1038/s41586-024-07606-7 (PMC11338834; doi:10.1038/s41586-024-07606-7)
Supplement: Supplementary file 1 — Supplementary Figs 1–45. [file 41586_2024_7606_MOESM1_ESM.pdf]

nature portfolio

https://doi.org/10.1038/s41586-024-07606-7

Supplementary information

Single-cell multiregion dissection of Alzheimer's disease

In the format provided by the authors and unedited

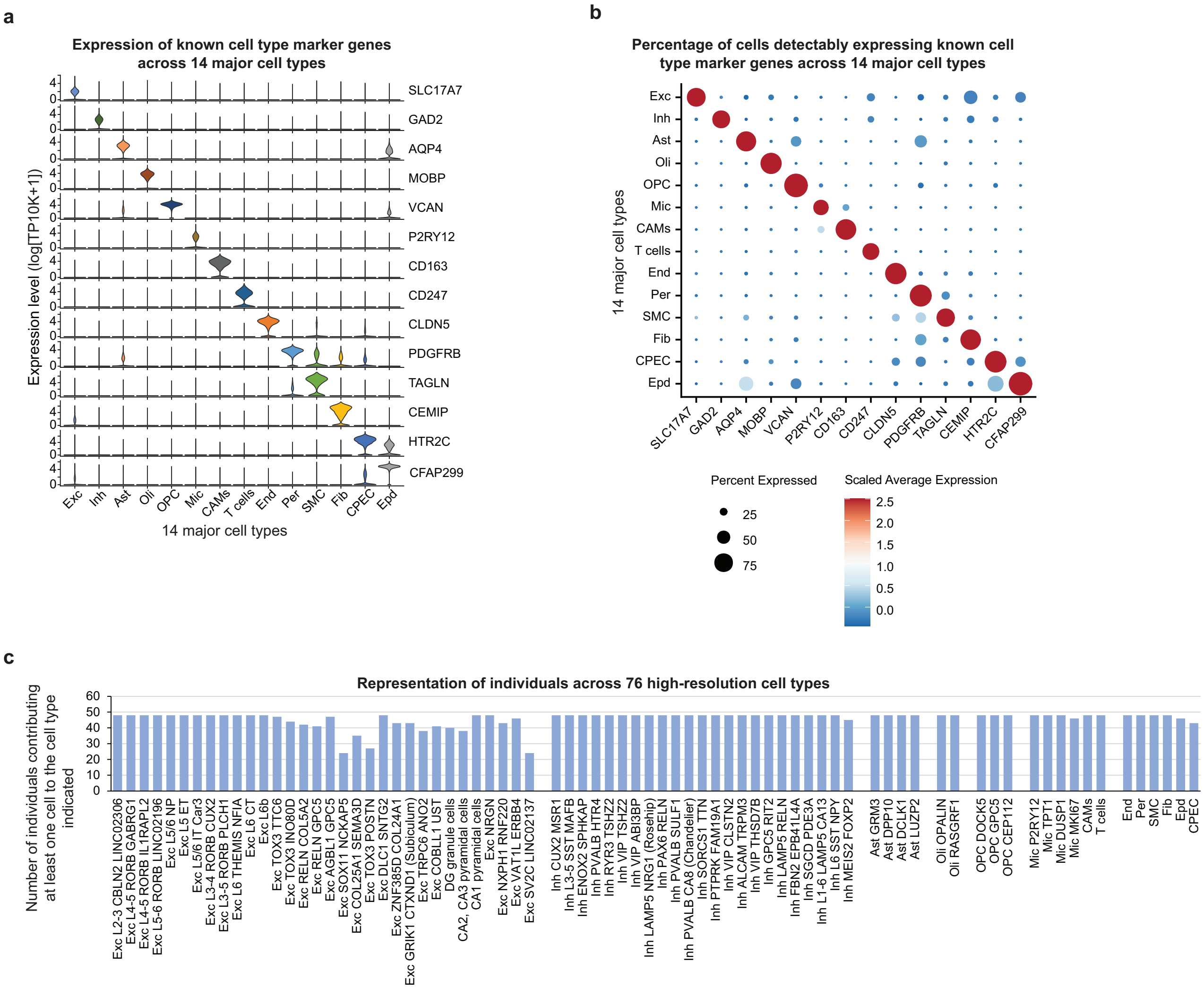

Supplementary Figure 1

Supplementary Figure 1. Overview of the study sample and major cell type annotations. a, Violin plot showing the expression of known cell type marker genes in each major cell type. **b,** Dot plot showing the scaled average expression of known cell type marker genes in each major cell type. **c,** Representation of individuals across cell types. The bar plot shows the number of study participants contributing at least one cell to the cell type indicated. Most cell type clusters contain cells from the majority of study participants.

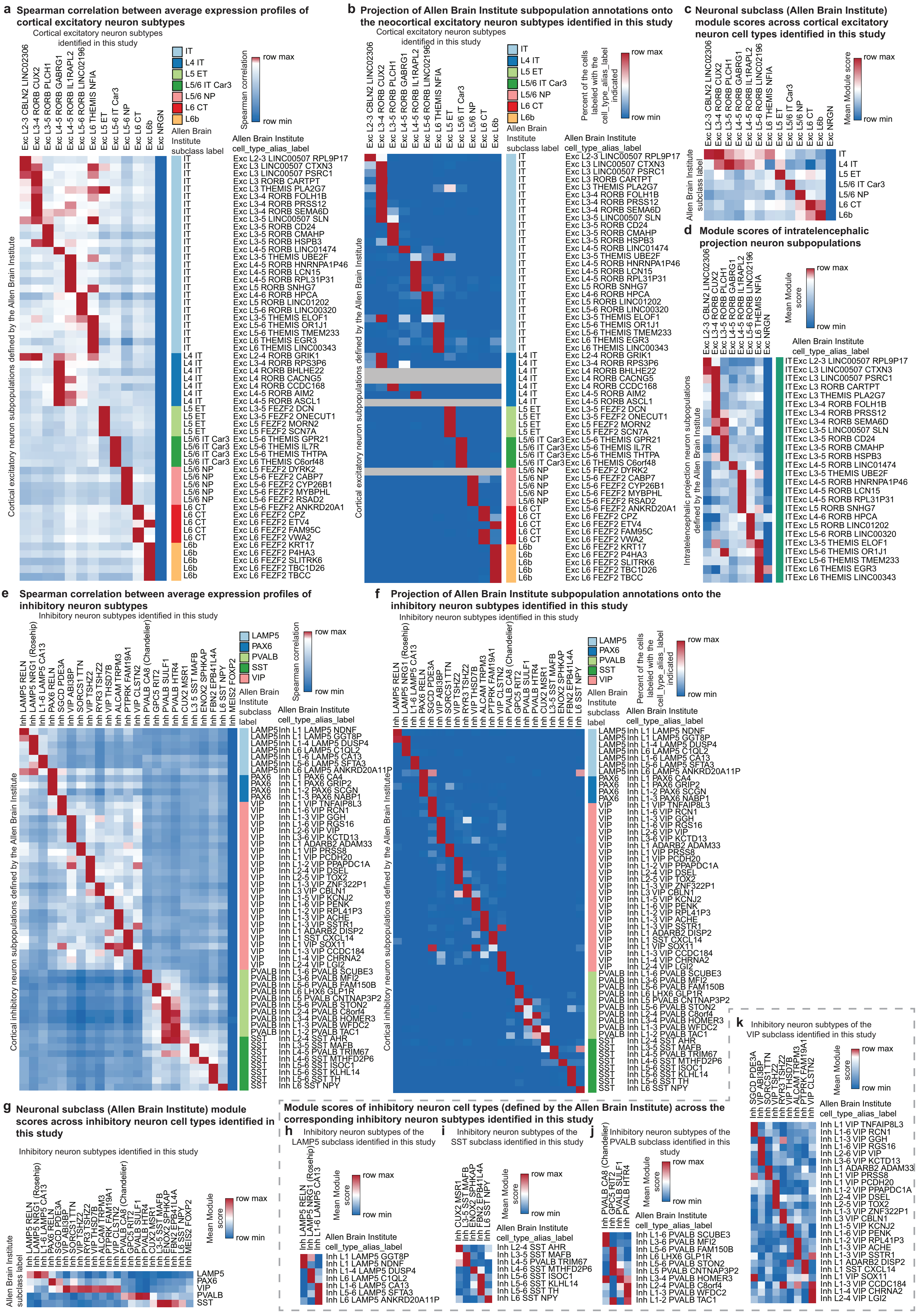

Supplementary Figure 2

Supplementary Figure 2. Cortical excitatory and inhibitory neuron subtypes align with previously identified neuronal subpopulations. a, Spearman correlation between the average expression profiles of cortical excitatory neuron subpopulations previously defined by the Allen Brain Institute (rows) and the neocortical excitatory neuron subtypes identified in this study (columns). **b**, Projection of cortical excitatory neuron subpopulation annotations previously defined by the Allen Brain Institute (rows) onto the neocortical excitatory neuron subtypes identified in this study (columns). The heat map shows the proportion of cells (rows) that were labeled with the cell type alias label (columns) indicated. **c,** Excitatory neuron subclass (Allen Brain Institute) module scores across cortical excitatory neuron cell types identified in this study (columns). The module scores were computed on the basis of the expression of excitatory neuron subclass marker gene sets. **d,** Module scores of intratelencephalic projection neuron subpopulations previously defined by the Allen Brain Institute (rows) across the corresponding cortical excitatory neuron cell types identified in this study (columns). **e,** Spearman correlation between the average expression profiles of inhibitory neuron subpopulations previously defined by the Allen Brain Institute (rows) and the inhibitory neuron subtypes identified in this study (columns). **f,** Projection of inhibitory neuron subpopulation annotations previously defined by the Allen Brain Institute (rows) onto the inhibitory neuron subtypes identified in this study (columns). The heat map shows the proportion of cells (rows) that were labeled with the cell type alias label (columns) indicated. **g,** Inhibitory neuron subclass (Allen Brain Institute) module scores across inhibitory neuron cell types identified in this study (columns). The module scores were computed on the basis of the expression of inhibitory neuron subclass marker gene sets. **h-k,** Module scores of inhibitory neuron cell types previously defined by the Allen Brain Institute (rows) across the corresponding inhibitory neuron cell types identified in this study (columns) ((**h**) LAMP5 subclass of inhibitory neurons; (**i**) SST subclass of inhibitory neurons; (**j**) PVALB subclass of inhibitory neurons; (**k**) VIP subclass of inhibitory neurons).

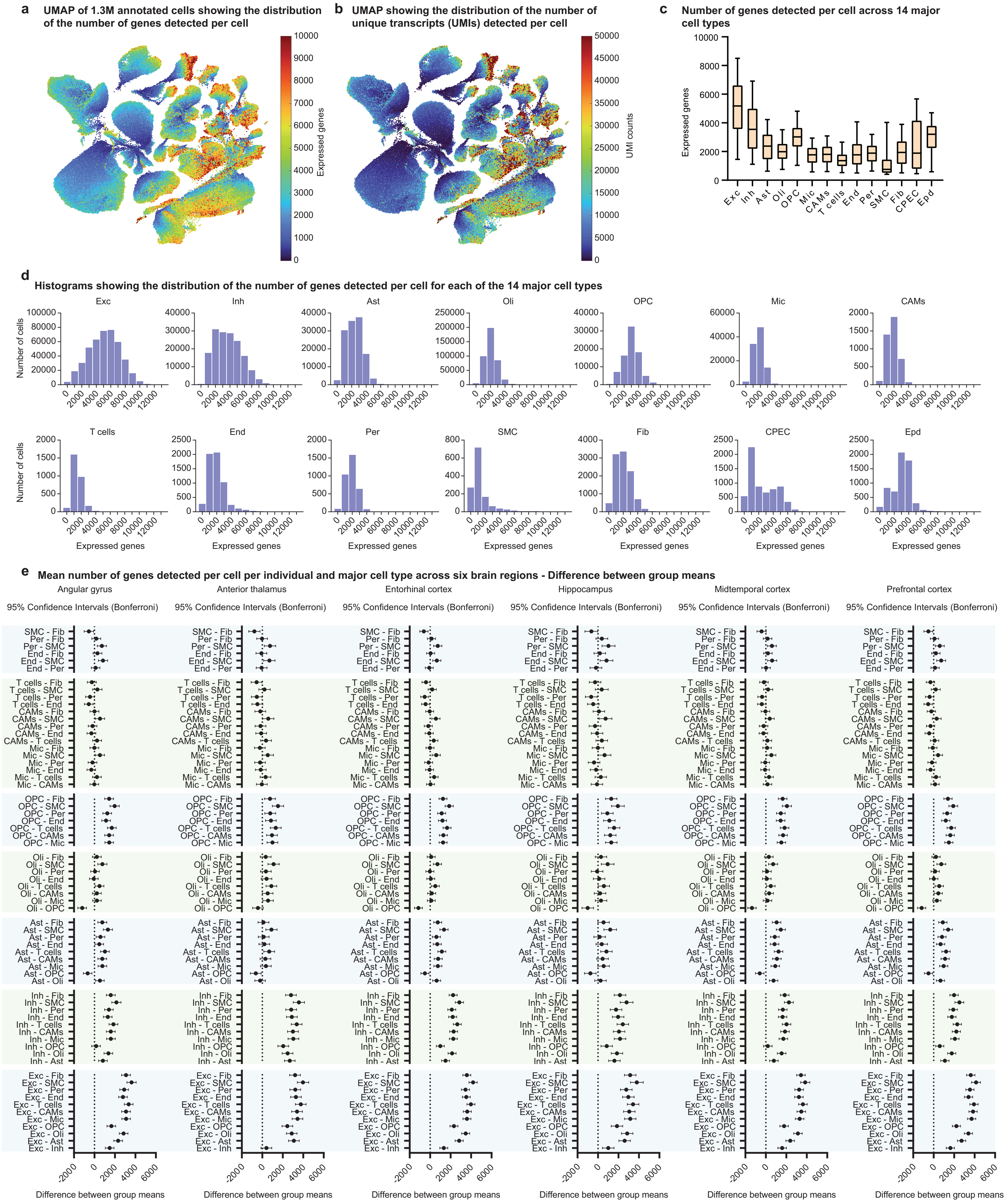

Supplementary Figure 3

Supplementary Figure 3. Global properties of gene expression: number of expressed genes and transcripts per cell. a,b, UMAP plots of all annotated cells showing the distribution of (**a**) the number of genes detected per cell and (**b**) the number of unique transcripts (UMIs) detected per cell. **c,** Mean number of genes detected per cell per individual and major cell type across the six brain regions analyzed. Within each box, horizontal lines denote median values; boxes extend from the 25th to the 75th percentile of each group's distribution of values; whiskers extend from the 5th to the 95th percentile (ordinary one-way ANOVA corrected for multiple comparisons using Bonferroni’s multiple comparisons test). **d,** Histograms showing the distribution of the number of genes detected per cell for each major cell type. **e,** Difference between group means with 95% confidence intervals (ordinary one-way ANOVA corrected for multiple comparisons using Bonferroni’s multiple comparisons test).

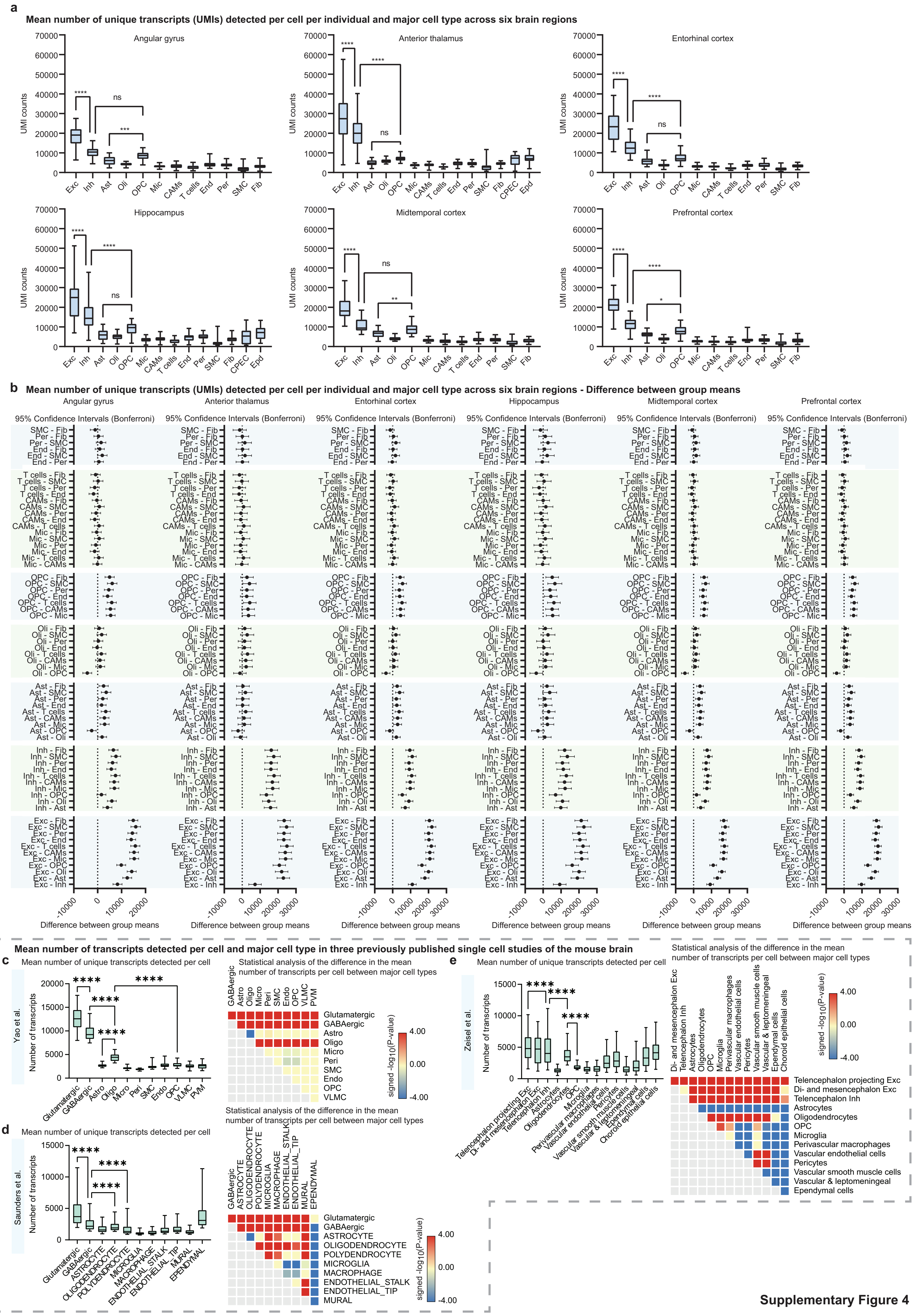

Supplementary Figure 4

Supplementary Figure 4. Global properties of gene expression: number of transcripts per cell. a,b, Mean number of unique transcripts (UMIs) detected per cell per individual and major cell type across the six brain regions analyzed. (**a**) Box plots of the mean number of unique transcripts detected per cell per individual (AG, HC, MT, PFC: n = 48; TH: n = 45; EC: n = 46, where 'n' denotes the number of individuals). Within each box, horizontal lines denote median values; boxes extend from the 25th to the 75th percentile of each group's distribution of values; whiskers extend from the 5th to the 95th percentile. ****P < 0.0001, ***P < 0.001, **P < 0.01, *P < 0.05; ns, P > 0.05; AG Ast vs. OPC: P = 0.0005; MT Ast vs. OPC: P = 0.0039; PFC Ast vs. OPC: P = 0.0283 (ordinary one-way ANOVA corrected for multiple comparisons using Bonferroni's multiple comparisons test). (**b**) Difference between group means with 95% confidence intervals (ordinary one-way ANOVA corrected for multiple comparisons using Bonferroni's multiple comparisons test). **c-e,** Mean number of unique transcripts (UMIs) detected per cell and major cell type in three previously published single cell studies of the mouse brain. Box plots show the mean number of unique transcripts detected per cell. Within each box, horizontal lines denote median values; boxes extend from the 25th to the 75th percentile of each group's distribution of values; whiskers extend from the 5th to the 95th percentile. ****P < 0.0001 (ordinary one-way ANOVA corrected for multiple comparisons using Bonferroni's multiple comparisons test). The heat maps show the statistical significance (signed $-\log_{10}$(Bonferroni-corrected P values)) of the difference in the mean number of transcripts per cell between major cell types. The signed P value score is the $-\log_{10}$(Bonferroni-corrected P value) with the sign of the difference (in the mean number of transcripts per cell) between the cell type indicated in the row minus the cell type indicated in the column. (**c**) Glutamatergic (n=49), GABAergic (n=49), Astro (n=49), Oligo (n=49), Micro (n=44), Peri (n=27), SMC (n=30), Endo (n=47), OPC (n=48), VLMC (n=29), PVM (n=25), where 'n' denotes the number of donor animals. (**d**) ASTROCYTE (n=16,713), ENDOTHELIAL_STALK (n=11,550), ENDOTHELIAL_TIP (n=1,240), EPENDYMAL (n=337), GABAergic (n=10,224), Glutamatergic (n=124,236), MACROPHAGE (n=414), MICROGLIA (n=1,449), MURAL (n=3,171), OLIGODENDROCYTE (n=22,121), POLYDENDROCYTE (n=3,304), where 'n' denotes the number of cells. (**e**) Telencephalon projecting excitatory neurons (n=18,799), Di- and mesencephalon inhibitory neurons (n=4,026), Telencephalon inhibitory interneurons (n=8,637), Astrocytes (n=19,377), Oligodendrocytes (n=30,253), Oligodendrocyte precursor cells (n=820), Microglia (n=5,425), Perivascular macrophages (n=2,029), Vascular endothelial cells (n=3,805), Pericytes (n=5,213), Vascular smooth muscle cells (n=1,628), Vascular and leptomeningeal cells (n=1,501), Ependymal cells (n=1,257), Choroid epithelial cells (n=458), where 'n' denotes the number of cells.

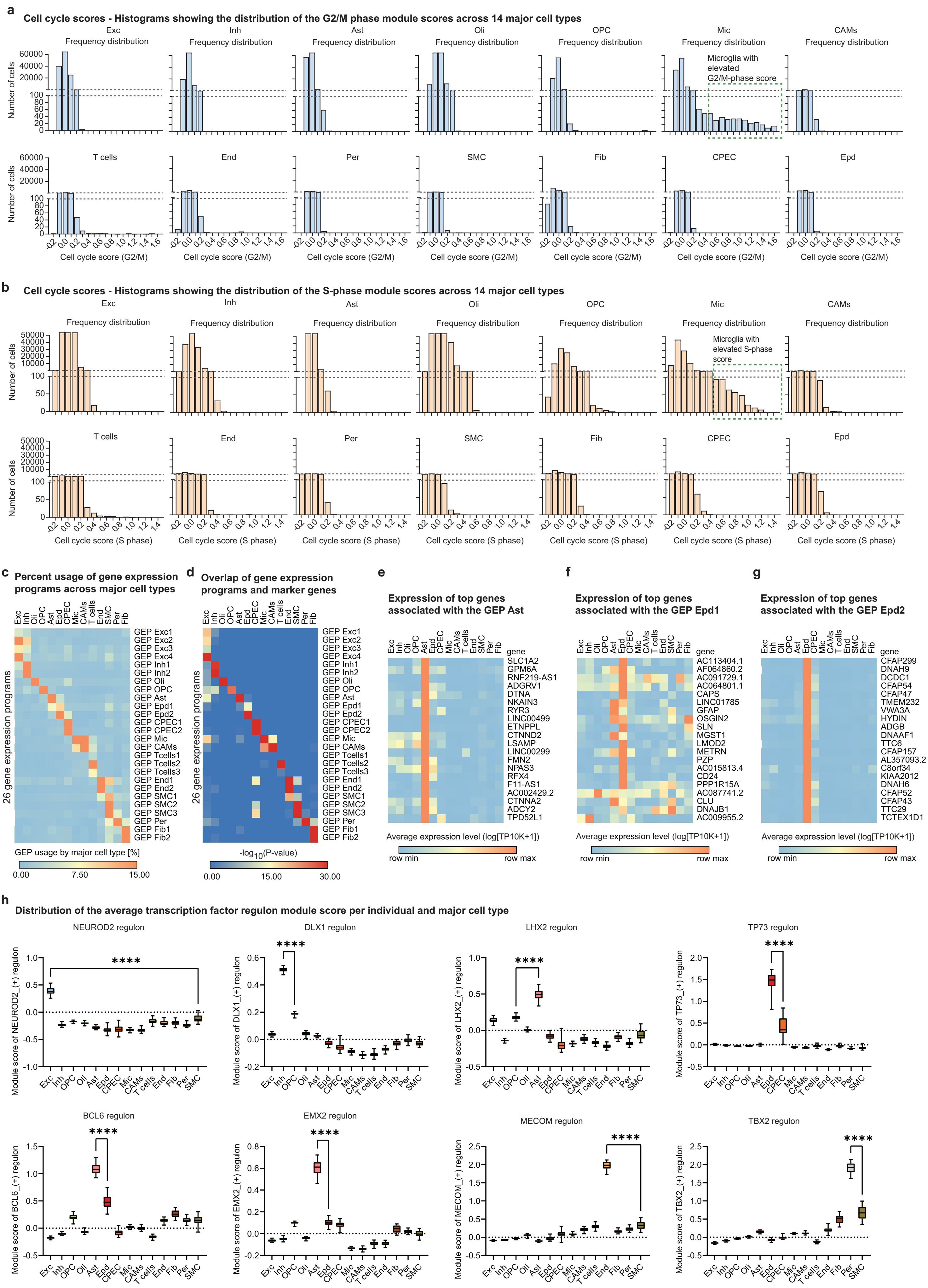

Supplementary Figure 5

Supplementary Figure 5. Cell cycle scores, gene expression programs, and key major cell type regulators. a,b, Histograms showing the distribution of (**a**) the G2/M- and (**b**) the S phase module scores. The module scores were computed based on the expression of gene sets associated with the S phase and the G2/M phase of the cell cycle. **c,** Heat map showing percent usage of all gene expression programs (GEPs) (rows) in all major cell types (columns). **d,** Overlap (one-sided Fisher's exact test) between the top 200 genes associated with the gene expression programs indicated (rows) and the major cell type marker genes (columns). The P values have been adjusted for multiple hypothesis testing; −log10(Bonferroni-corrected P values) are shown. **e-g,** Relative expression level of the top 20 genes associated with the gene expression programs (**e**) GEP Ast (preferentially used by astrocytes), (**f**) GEP Epd 1 (used by astrocytes and ependymal cells), and (**g**) GEP Epd 2 (preferentially used by ependymal cells) across all major cell types. **h,** Activity of selected transcription factor regulons across the major cell types. The box plots show the distribution of the average transcription factor regulon module score per individual and major cell type (n=48 individuals). Within each box, horizontal lines denote median values; boxes extend from the 25th to the 75th percentile of each group's distribution of values; whiskers extend from the 5th to the 95th percentile. ****P < 0.0001 (ordinary one-way ANOVA corrected for multiple comparisons using Šídák's multiple comparisons test).

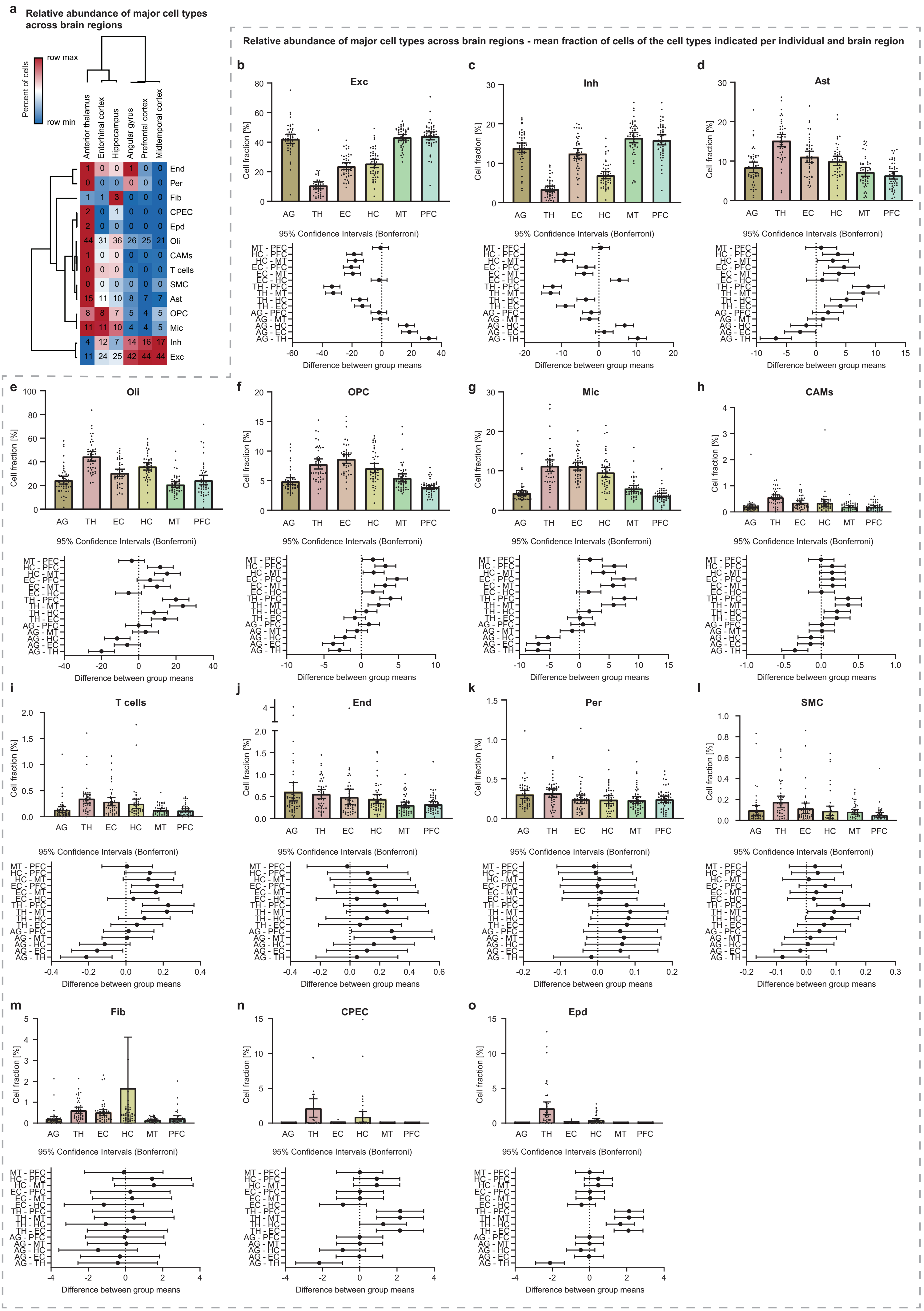

Supplementary Figure 6

Supplementary Figure 6. Relative abundance of major cell types across regions. a, Relative abundance of major cell types across regions. The heat map shows the proportion of each major cell type relative to all the cells isolated from a brain region. **b-o,** Relative abundance of major cell types across brain regions (AG, HC, MT, PFC: n = 48; TH: n = 45; EC: n = 46). Difference between brain region group means with 95% confidence intervals (ordinary one-way ANOVA corrected for multiple comparisons using Bonferroni's multiple comparisons test).

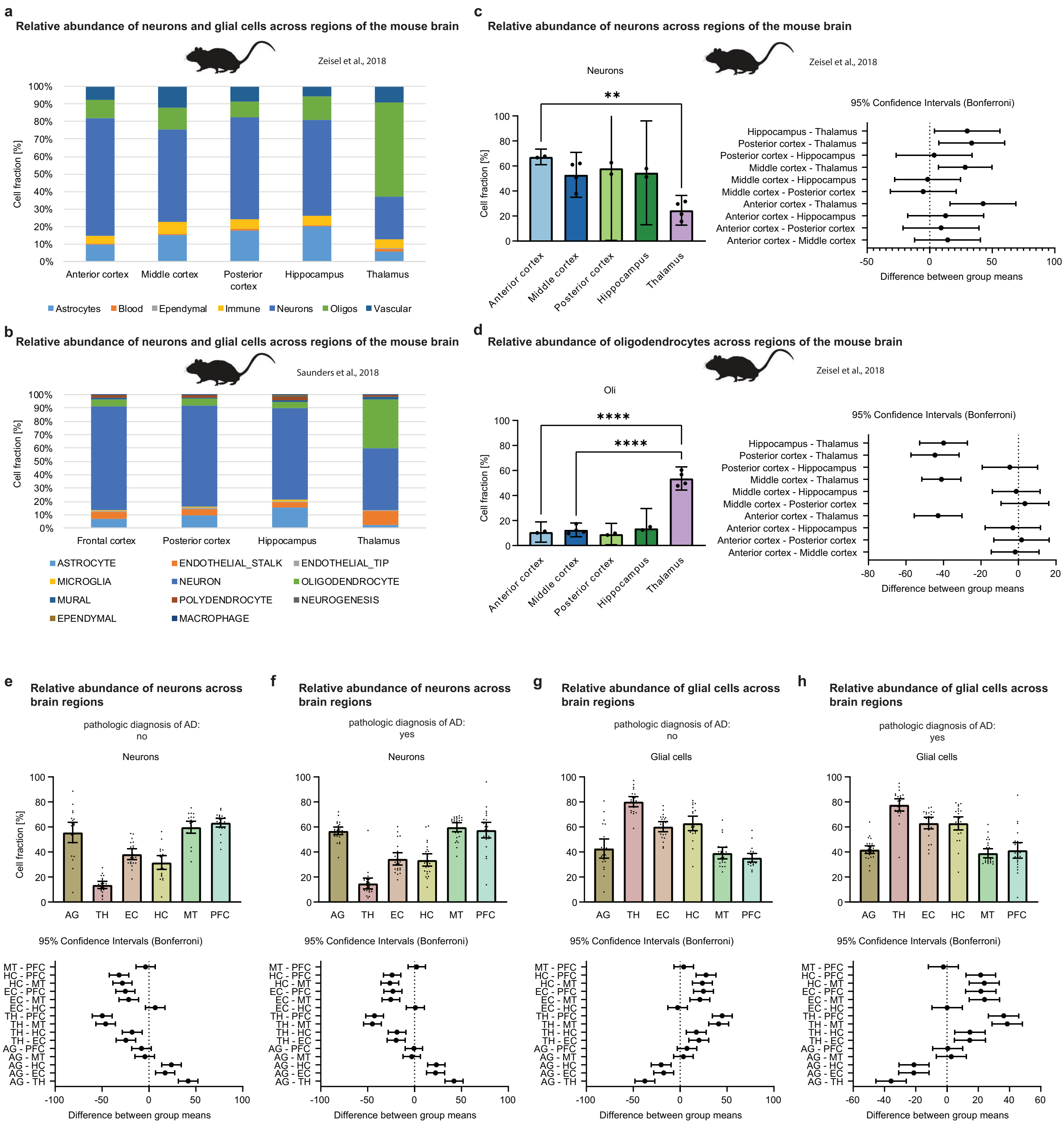

Supplementary Figure 7

Supplementary Figure 7. Relative abundance of neurons and glial cells across brain regions. a-d, Relative abundance of neurons and glial cells across regions of the mouse brain. The bar plots show the proportion of the cell types indicated relative to all the cells isolated from a brain region (based on the studies by (**a**) Zeisel et al. 2018 and (**b**) Saunders et al. 2018). (**c,d**) Relative abundance of (**c**) neurons and (**d**) oligodendrocytes across regions of the mouse brain. The bar plots show the mean fraction of cells of the cell types indicated (Anterior cortex, Posterior cortex, Hippocampus: n = 2; Middle cortex and thalamus: n = 4). Data are expressed as mean with 95% confidence intervals and individual data points are shown. ****$P < 0.0001$, **$P < 0.01$; Anterior cortex vs. Thalamus (Neurons): $P = 0.0021$ (ordinary one-way ANOVA corrected for multiple comparisons using Bonferroni's multiple comparisons test). Right panels: difference between group means with 95% confidence intervals (ordinary one-way ANOVA corrected for multiple comparisons using Bonferroni's multiple comparisons test). **e-h,** Relative abundance of neurons and glial cells across brain regions in individuals with (**f,h**) and without (**e,g**) a pathologic diagnosis of Alzheimer's disease. The bar plots show the mean fraction of neurons (**e,f**) and glial cells (**g,h**) per individual and brain region ((**e,g**) AG, EC, HC, MT, PFC: n = 22; TH: n = 21; (**f,h**) AG, HC, MT, PFC: n = 26; TH: n = 24; EC: n = 24). The fraction of neurons and glial cells was computed relative to all the cells isolated from a brain region of an individual. Data are expressed as mean with 95% confidence intervals and individual data points are shown. Lower panels: difference between group means with 95% confidence intervals (ordinary one-way ANOVA corrected for multiple comparisons using Bonferroni's multiple comparisons test).

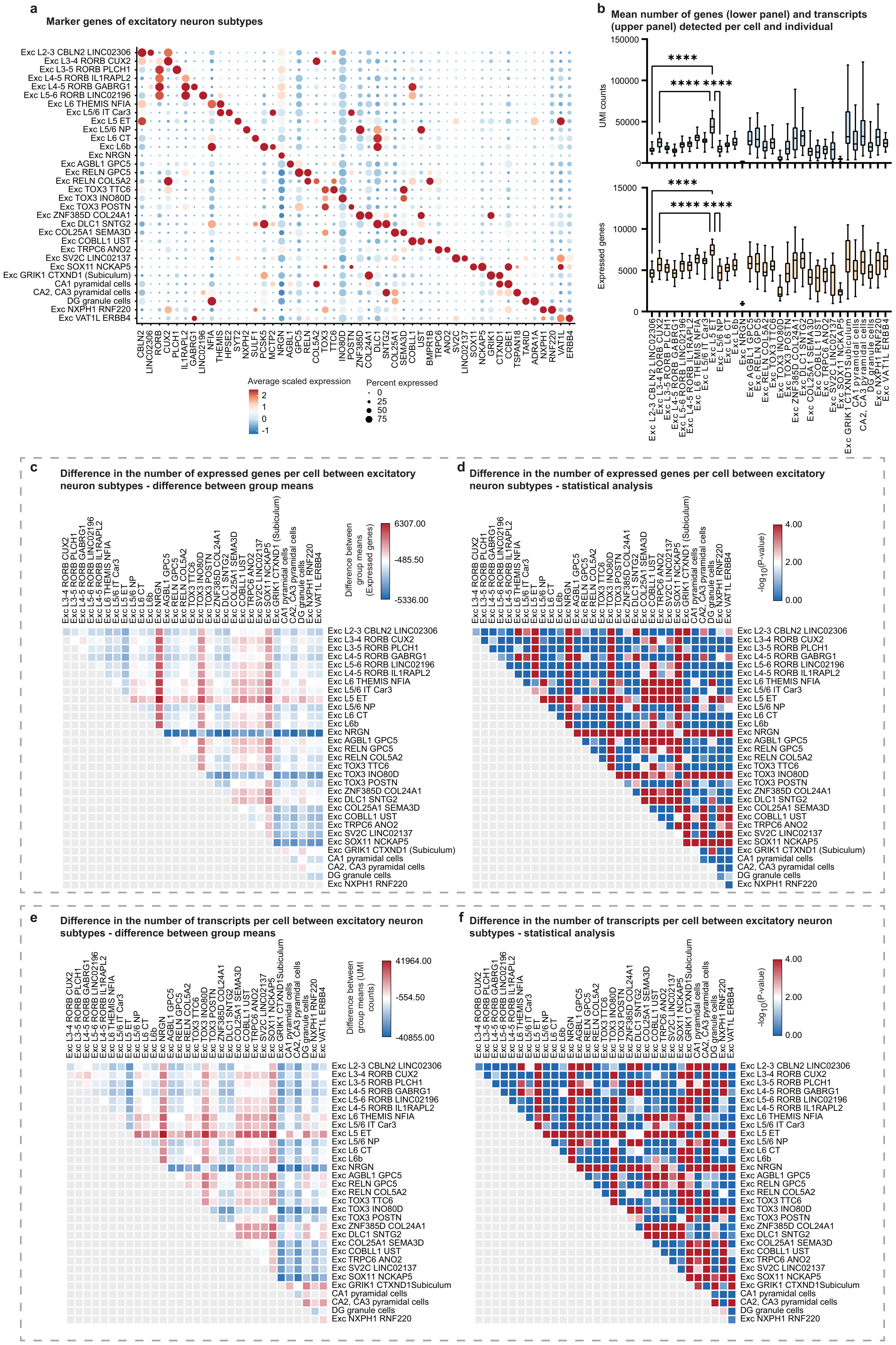

Supplementary Figure 8

Supplementary Figure 8. Marker genes, number of expressed genes and transcripts per cell across excitatory neuron subtypes. a, Marker genes of excitatory neuron subtypes. The dot plot shows the average scaled expression of the marker genes indicated across all excitatory neuron subtypes. **b,** Mean number of genes (lower panel) and transcripts (upper panel) detected per cell and individual. Box plots show the distribution of the mean number of genes or transcripts detected per cell and individual (n=48 individuals). Within each box, horizontal lines denote median values; boxes extend from the 25th to the 75th percentile of each group's distribution of values; whiskers extend from the 5th to the 95th percentile. ****$P < 0.0001$ (two-sided, ordinary one-way ANOVA corrected for multiple comparisons using Bonferroni's multiple comparisons test). **c,d,** Difference in the number of expressed genes per cell between excitatory neuron subtypes. The heat map in panel (**c**) shows the difference between group means shown in the lower panel of (b) and the heat map in panel (**d**) shows the $-\log_{10}$(Bonferroni-corrected P values) (two-sided, ordinary one-way ANOVA corrected for multiple comparisons using Bonferroni's multiple comparisons test). **e,f,** Difference in the number of transcripts per cell between excitatory neuron subtypes. The heat map in panel (**e**) shows the difference between group means shown in the upper panel of (**b**) and the heat map in panel (**f**) shows the $-\log_{10}$(Bonferroni-corrected P values) (two-sided, ordinary one-way ANOVA corrected for multiple comparisons using Bonferroni's multiple comparisons test).

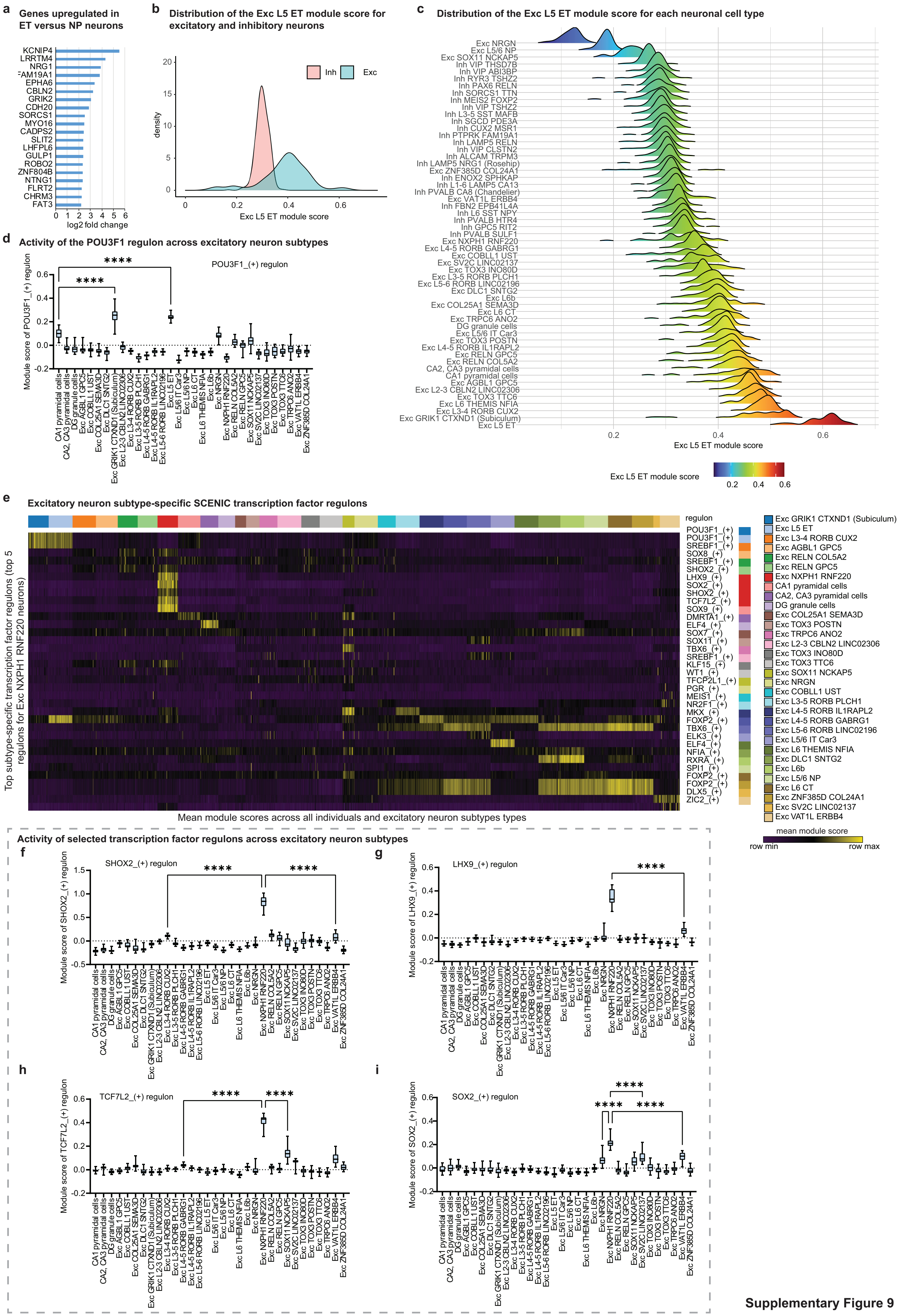

Supplementary Figure 9

Supplementary Figure 9. Gene expression programs and key regulators underlying excitatory neuron diversity. a, Top 20 genes up-regulated in extratelencephalic projection neurons (Exc L5 ET) compared to near-projecting excitatory neurons in layers 5 and 6 (Exc L5/6 NP). **b,** Density plot showing the distribution of the Exc L5 ET module score for excitatory and inhibitory neurons. The Exc L5 ET module score was computed on the basis of the expression of genes significantly up-regulated in extratelencephalic projection neurons (Exc L5 ET) compared to near-projecting excitatory neurons in layers 5 and 6 (Exc L5/6 NP). **c,** Ridgeline plot showing the distribution of the Exc L5 ET module score for each neuronal cell type. **d,** Activity of the POU3F1 regulon across the excitatory neuron subtypes. The box plots show the distribution of the average regulon module score per individual and cell type (n=48 individuals). Within each box, horizontal lines denote median values; boxes extend from the 25th to the 75th percentile of each group's distribution of values; whiskers extend from the 5th to the 95th percentile. ****P < 0.0001 (ordinary one-way ANOVA corrected for multiple comparisons using Šídák's multiple comparisons test). **e,** Identification of excitatory neuron subtype-specific SCENIC transcription factor regulons. The heat map shows the mean module score of the top transcription factor regulons (top 5 regulons for Exc NXPH1 RNF220 neurons) (rows) across all individuals and excitatory neuron subtypes (columns). **f-i,** Activity of selected transcription factor regulons across the excitatory neuron subtypes. The box plots show the distribution of the average transcription factor regulon module score per individual and excitatory neuron subtype (n=48 individuals). Within each box, horizontal lines denote median values; boxes extend from the 25th to the 75th percentile of each group's distribution of values; whiskers extend from the 5th to the 95th percentile. ****P < 0.0001 (ordinary one-way ANOVA corrected for multiple comparisons using Šídák's multiple comparisons test).

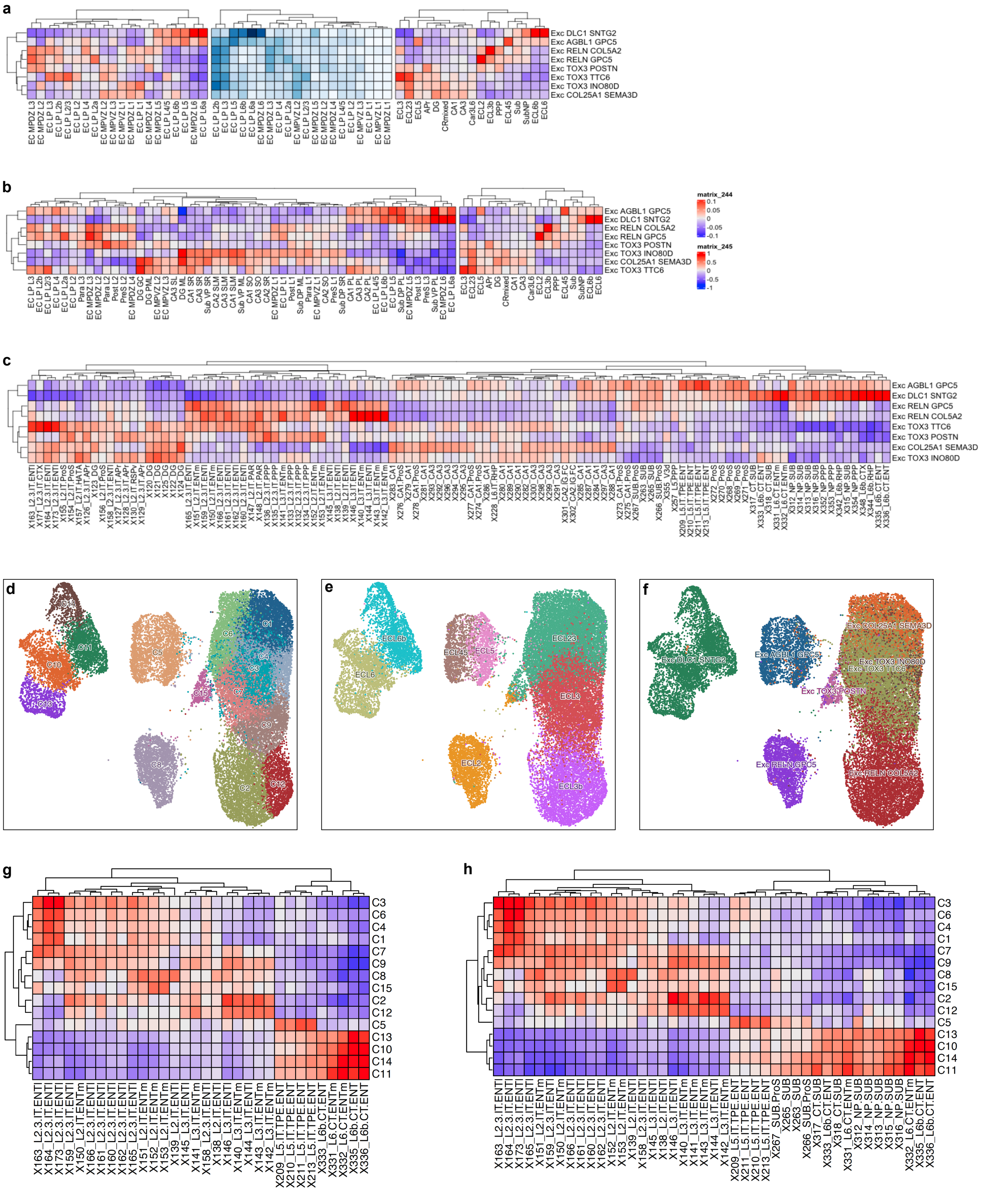

Supplementary Figure 10

Supplementary Figure 10. Entorhinal cortex neuron annotation. a, Similarity between human excitatory neuron signatures (rows) and mouse entorhinal cortex substructure signatures computed from spatial transcriptomics data from (Ortiz et al. 2020) (columns) (left, center)[42], and human signatures for cell type annotations previously reported in (Davila-Velderrain et al. 2021) (columns) (**a**, right)[94]. **b,** Similar to (**a**) but including all mouse hippocampal and retrohippocampal substructure signatures (left). **c,** Similarity with all mouse hippocampal and retrohippocampal reference transcriptional excitatory neuron types reported by the Allen Institute in (Yao et al. 2021)[43]. **d-f,** 2D projection of human excitatory neurons from the entorhinal cortex labeled with clusters (**d**), best-matching substructures from spatial transcriptomics and reference transcriptional cell types, (**c**) consensus annotations reported herein. Analysis performed with ACTIONet (Mohammadi et al. 2020)[95]. **g-h,** Similarity between human cluster signatures from (**d**) (rows) and mouse reference transcriptional cell types (columns).

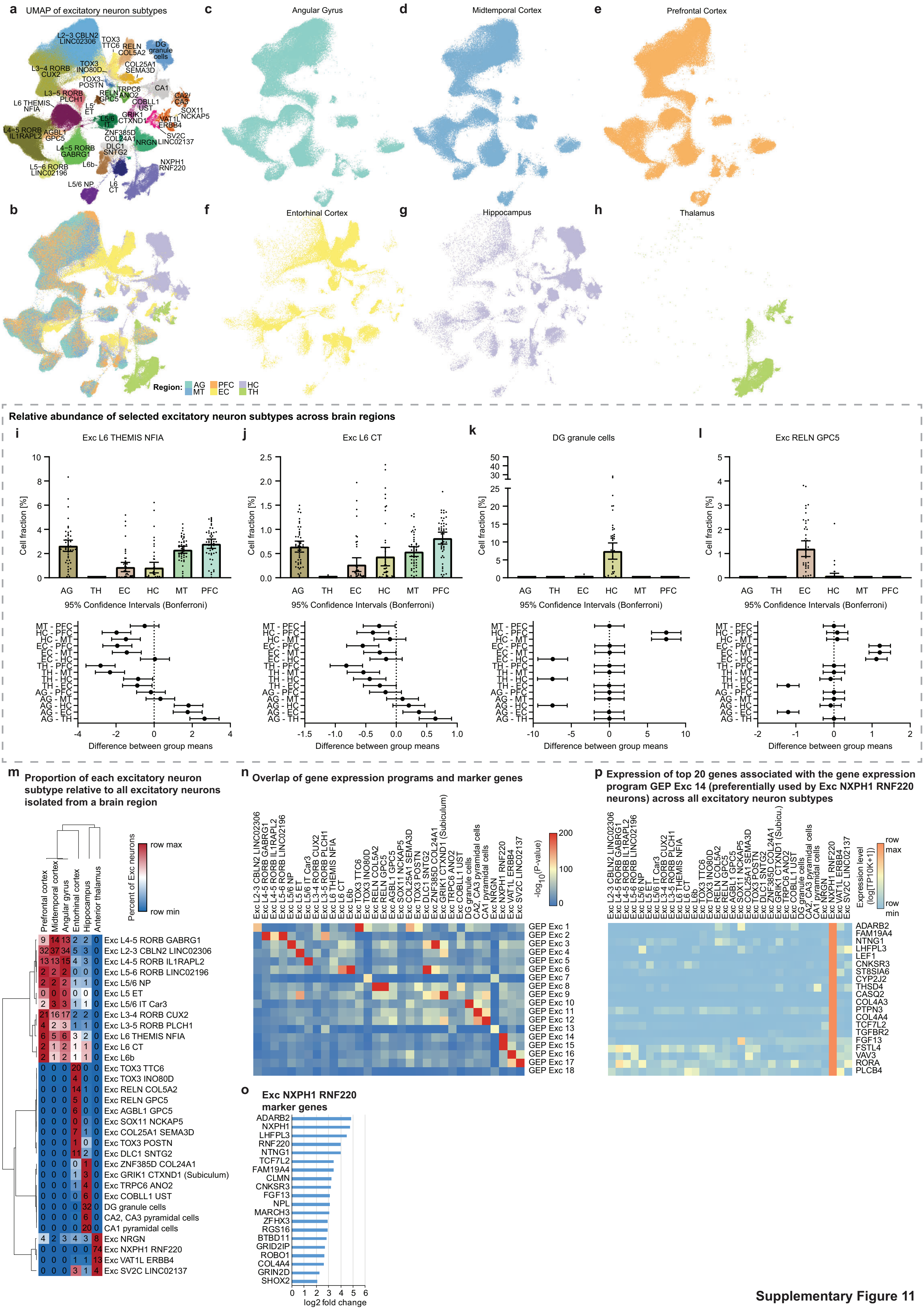

Supplementary Figure 11

Supplementary Figure 11. Excitatory neuron composition across regions and gene expression programs. a, UMAP visualization of excitatory neurons coloured by cell type. Cells isolated from all six regions are shown. **b,** UMAP visualization of excitatory neurons coloured by the brain region the cells were isolated from. **c-h,** UMAP visualization of excitatory neurons separated by the brain regions indicated. **i-l,** Relative abundance of selected excitatory neuron subtypes across brain regions. The bar plots show the mean fraction of cells of the subtype indicated per individual and brain region (AG, HC, MT, PFC: n = 48; TH: n = 45; EC: n = 46). The fraction of cells of a subtype was computed relative to all the cells isolated from a brain region of an individual. Data are expressed as mean with 95% confidence intervals and individual data points are shown. Lower panels: difference between group means with 95% confidence intervals (ordinary one-way ANOVA corrected for multiple comparisons using Bonferroni's multiple comparisons test). **m,** Relative abundance of excitatory neuron subtypes across brain regions. The heat map shows the proportion of each excitatory neuron subtype relative to all excitatory neurons isolated from a brain region. **n,** Overlap (one-sided Fisher's exact test) between the top 200 genes associated with the gene expression programs indicated (rows) and the excitatory neuron marker genes (columns). The P values have been adjusted for multiple hypothesis testing; $-\log_{10}$(Bonferroni-corrected P values) are shown. **o,** Top 20 marker genes of Exc NXPH1 RNF220 neurons. The bar plot shows the $\log_2$ fold change of the expression level of the genes indicated in Exc NXPH1 RNF220 neurons compared to all other excitatory neuron subtypes. **p,** Relative expression level of the top 20 genes associated with the gene expression program GEP Exc 14 across all excitatory neuron subtypes.

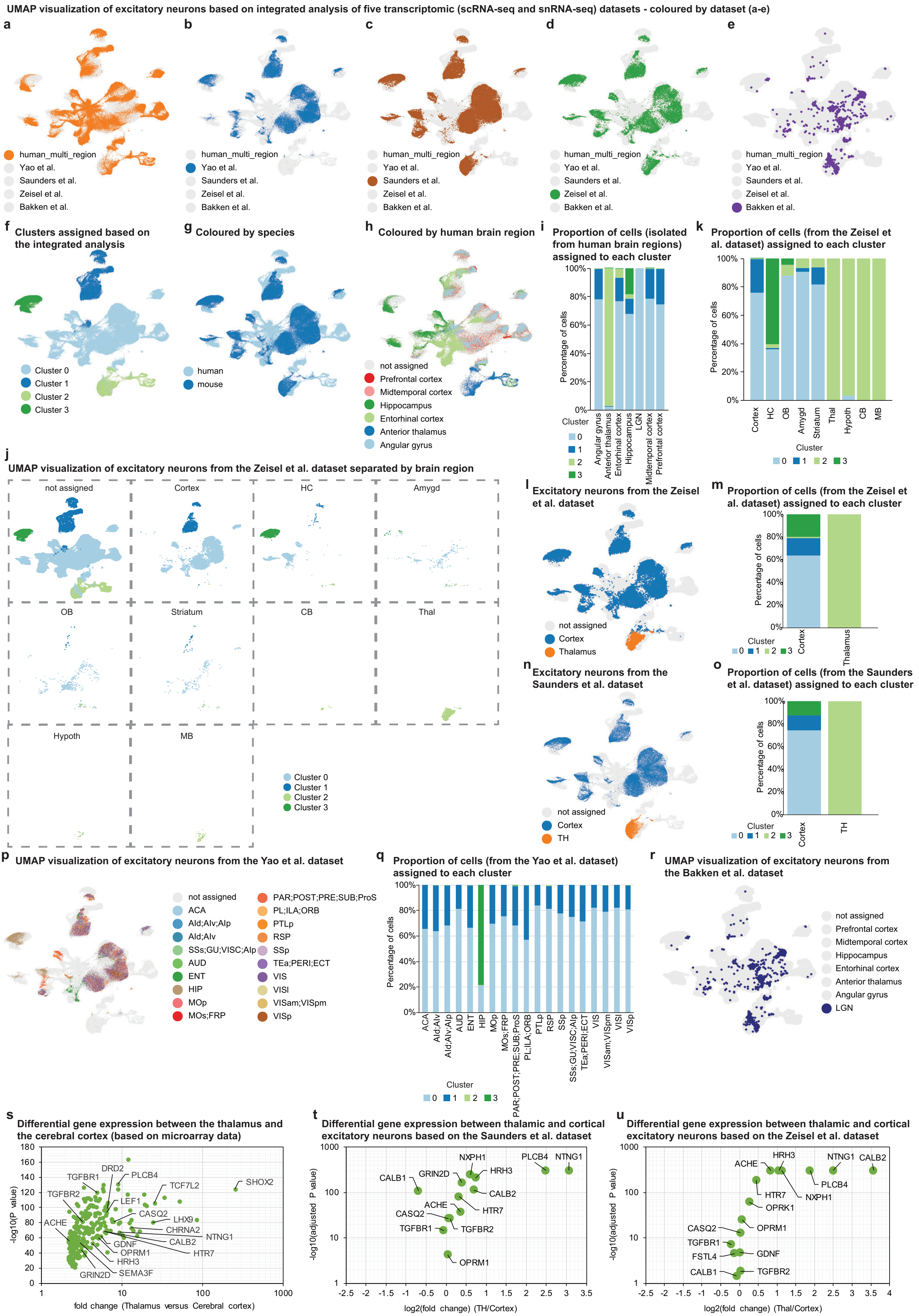

Supplementary Figure 12

Supplementary Figure 12. Aligning human and mouse excitatory neurons. a-h, UMAP visualization of excitatory neurons based on integrated analysis of five transcriptomic (scRNA-seq and snRNA-seq) datasets. Cells and nuclei are coloured by dataset (**a-e**), the clusters assigned based on the integrated analysis (**f**), species (**g**), and human brain region (**h**). **i,** Cluster composition. Bar plot showing the proportion of cells (y axis) from each human brain region (x axis) assigned to each of the four clusters identified based on the integrated analysis. LGN (dorsal lateral geniculate nucleus). **j,** UMAP visualization of excitatory neurons from the Zeisel et al. dataset separated by the brain regions indicated. Cells are coloured by the clusters assigned based on the integrated analysis. HC (Hippocampus), Amygd (Amygdala), OB (Olfactory bulb), CB (Cerebellum), Thal (Thalamus), Hypoth (Hypothalamus), MB (Midbrain), SC (Spinal Cord). **k,** Cluster composition of the excitatory neurons from the Zeisel et al. dataset. Bar plot showing the proportion of cells (y axis) from each brain region (x axis) assigned to each of the four clusters identified based on the integrated analysis. **l-q,** Cluster composition (**m,o,q**) and UMAP visualization of excitatory neurons (**l,n,p**) from the Zeisel et al. dataset (**l,m**), the Saunders et al. dataset (**n,o**), and the Yao et al. dataset (**p,q**). Cells are coloured by the brain regions indicated. Bar plots show the proportion of cells (y axis) from each brain region (x axis) assigned to each of the four clusters identified based on the integrated analysis. TH (thalamus) (**n,o**). Cortex (CTX) areas included in the Yao et al. dataset are: visual (VIS), frontal pole (FRP), primary motor (MOp), secondary motor (MOs), primary somatosensory (SSp), supple-mental somatosensory (SSs), gustatory (GU), visceral (VISC), auditory (AUD), primary visual (VISp), anteromedial visual (VISam), lateral visual (VISl), posteromedial (VISpm), anterior cingulate (ACA), prelimbic (PL), infra-limbic (ILA), orbital (ORB), agranular insular (AI) (divided into dorsal (AId), ventral (AIv), and posterior (AIp) parts), retrosplenial (RSP), posterior parietal association (PTLp), temporal association (TEa), perirhinal (PERI), and ectorhinal (ECT) areas. Covered regions in HPF (hippocampal formation) are divided into two main parts: the hippocampal region (HIP), including fields CA1, CA2, CA3, and dentate gyrus (DG), and the retrohippocampal region (RHP), including lateral entorhinal area (ENTl), medial entorhinal area (ENTm), parasubiculum (PAR), postsubiculum (POST), presubiculum (PRE), subiculum (SUB), and prosubiculum (ProS). **r,** UMAP visualization of excitatory neurons from the Bakken et al. dataset. Nuclei are coloured by the brain region indicated. **s,** Volcano plot showing the fold change in the expression of selected genes between the thalamus and the cerebral cortex based on the analysis of microarray data from different human brain regions (Hawrylycz et al., 2012) using the Differential Search tool of the Allen Brain Atlas data portal. P values were determined using one-sided two-sample t-tests, followed by adjustment for multiple comparisons with the Benjamini-Hochberg false discovery rate correction, as implemented in the Differential Search tool of the Allen Brain Atlas data portal. **t,u,** Volcano plots showing the fold change in the expression of selected genes in excitatory neurons between the thalamus and the cortex based on the Saunders et al. dataset (**t**) and the Zeisel et al. dataset (**u**). P values were derived using the Wilcoxon rank-sum test, with adjustment for multiple comparisons via the Bonferroni method, as implemented in Seurat's FindMarkers function.

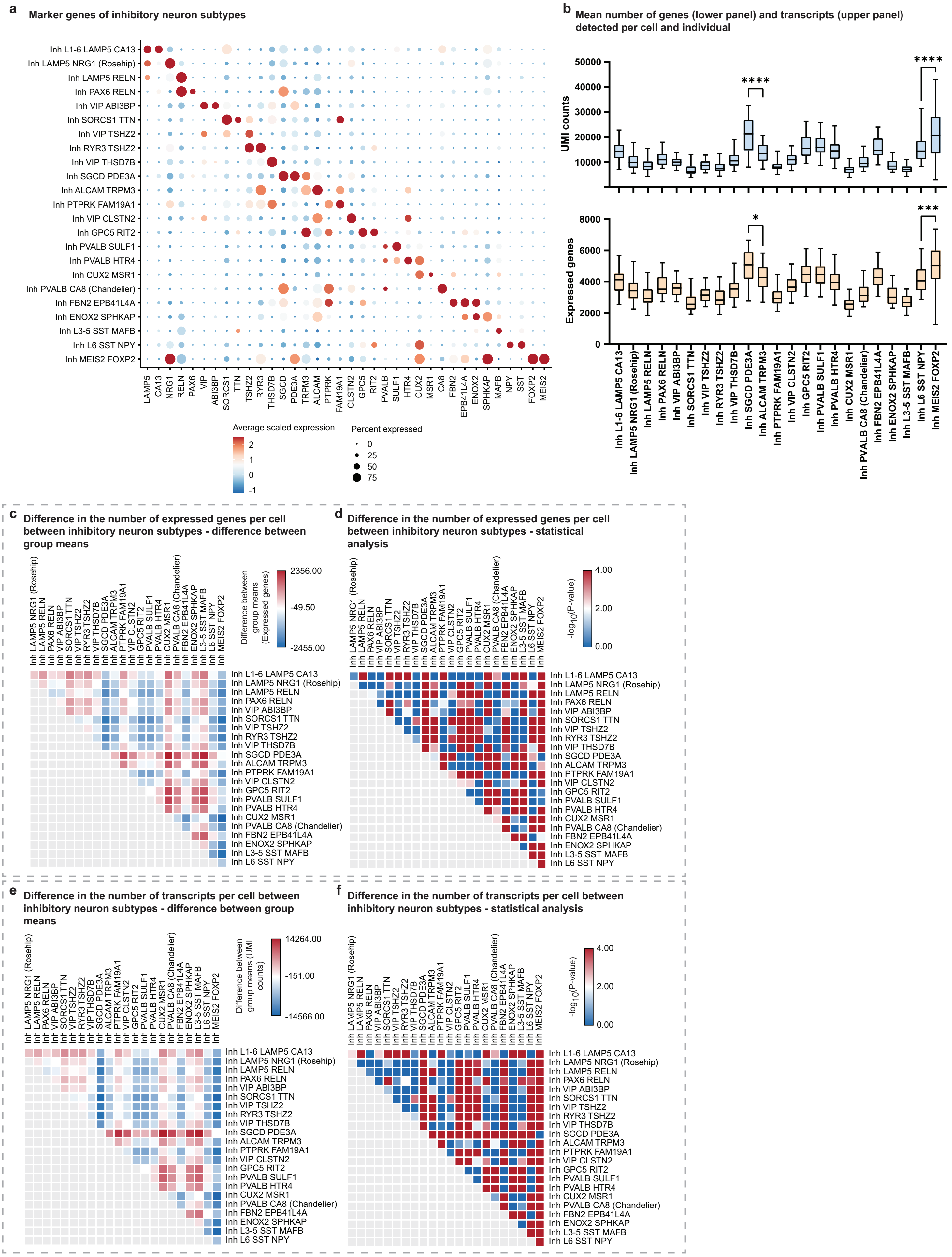

Supplementary Figure 13

Supplementary Figure 13. Marker genes, number of expressed genes and transcripts per cell across inhibitory neuron subtypes. a, Marker genes of inhibitory neuron subtypes. The dot plot shows the average scaled expression of the marker genes indicated across all inhibitory neuron subtypes. **b,** Mean number of genes (lower panel) and transcripts (upper panel) detected per cell and individual (n=48 individuals). Box plots show the distribution of the mean number of genes or transcripts detected per cell and individual. Within each box, horizontal lines denote median values; boxes extend from the 25th to the 75th percentile of each group's distribution of values; whiskers extend from the 5th to the 95th percentile. ****P < 0.0001, ***P < 0.001, *P < 0.05; Inh SGCD PDE3A vs. Inh ALCAM TRPM3 (expressed genes): P = 0.0223; Inh L6 SST NPY vs. Inh MEIS2 FOXP2 (expressed genes): P = 0.0001 (ordinary one-way ANOVA corrected for multiple comparisons using Bonferroni's multiple comparisons test). **c,d,** Difference in the number of expressed genes per cell between inhibitory neuron subtypes. The heat map in panel (**c**) shows the difference between group means shown in the lower panel of (**b**) and the heat map in panel (**d**) shows the $-\log_{10}$(Bonferroni-corrected P values) (ordinary one-way ANOVA corrected for multiple comparisons using Bonferroni's multiple comparisons test). **e,f,** Difference in the number of transcripts per cell between inhibitory neuron subtypes. The heat map in panel (**e**) shows the difference between group means shown in the upper panel of (**b**) and the heat map in panel (**f**) shows the $-\log_{10}$(Bonferroni-corrected P values) (ordinary one-way ANOVA corrected for multiple comparisons using Bonferroni's multiple comparisons test).

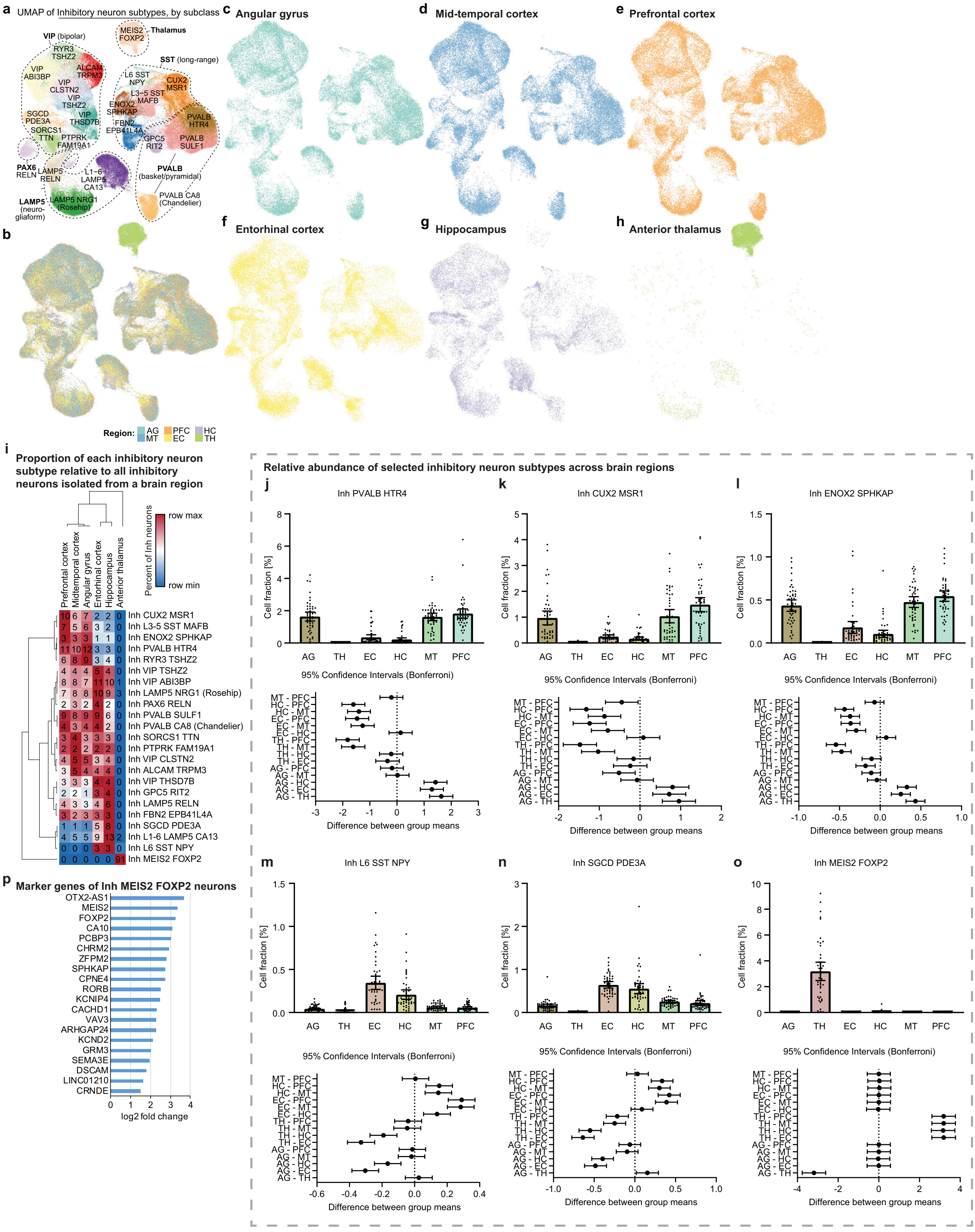

Supplementary Figure 14

Supplementary Figure 14. Inhibitory neuron diversity across different regions of the human brain. a, UMAP visualization of inhibitory neurons coloured by cell type. Cells isolated from all six regions are shown. **b,** UMAP visualization of inhibitory neurons coloured by the brain region the cells were isolated from. **c-h,** UMAP visualization of inhibitory neurons separated by the brain regions indicated. **i,** Relative abundance of inhibitory neuron subtypes across brain regions. The heat map shows the proportion of each inhibitory neuron subtype relative to all inhibitory neurons isolated from a brain region. **j-o,** Relative abundance of selected inhibitory neuron subtypes across brain regions. The bar plots show the mean fraction of cells of the cell types indicated per individual and brain region (AG, HC, MT, PFC: n = 48; TH: n = 45; EC: n = 46). The fraction of cells of a cell type was computed relative to all the cells isolated from a brain region of an individual. Data are expressed as mean with 95% confidence intervals and individual data points are shown. Lower panels: difference between group means with 95% confidence intervals (ordinary one-way ANOVA corrected for multiple comparisons using Bonferroni's multiple comparisons test). **p,** Top 20 marker genes of Inh MEIS2 FOXP2 neurons. The bar plot shows the $\log_2$ fold change of the expression level of the genes indicated in Inh MEIS2 FOXP2 neurons compared to all other inhibitory neuron subtypes.

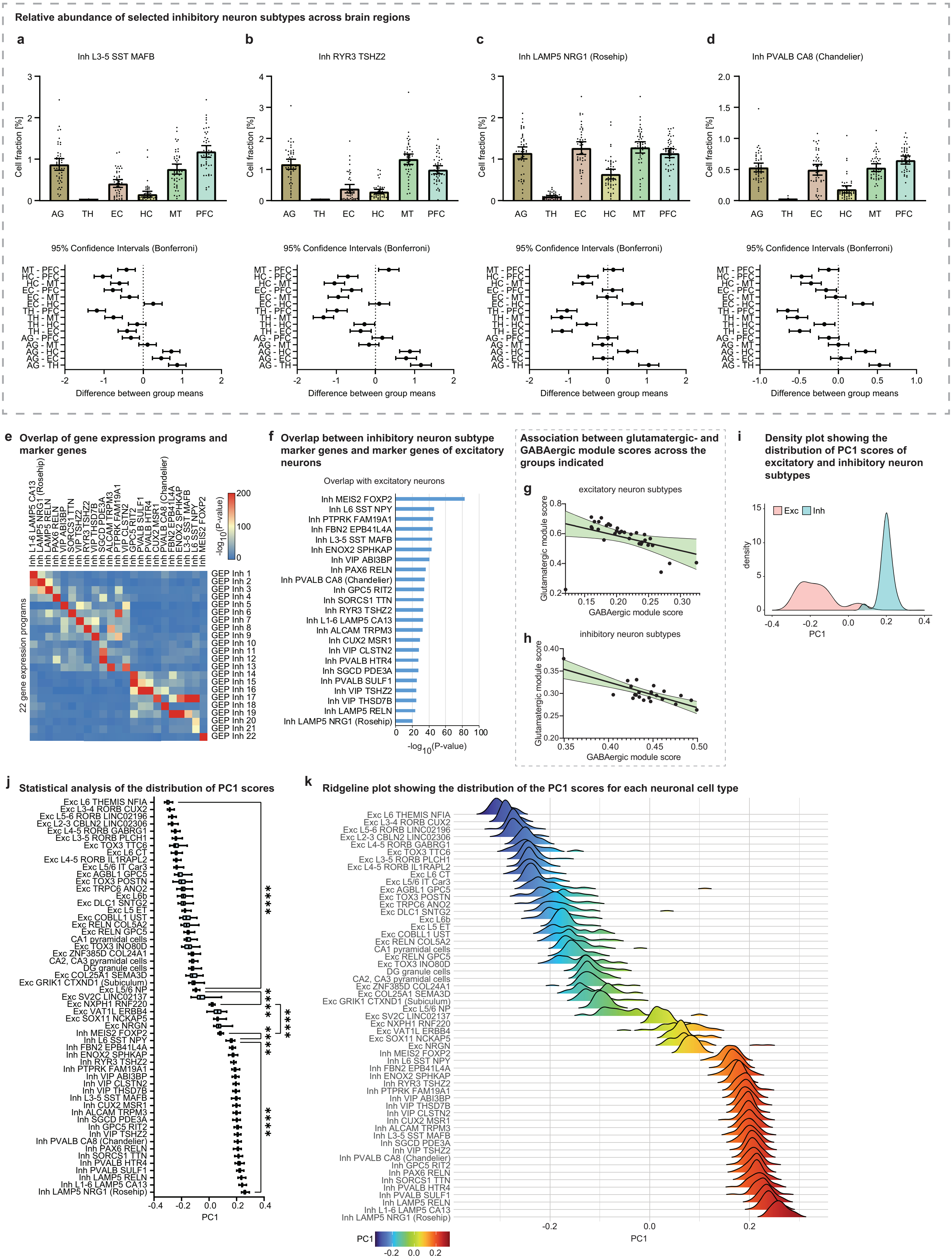

Supplementary Figure 15

Supplementary Figure 15. Gene expression programs and key regulators underlying inhibitory neuron diversity. a-d, Relative abundance of selected inhibitory neuron subtypes across brain regions. The bar plots show the mean fraction of cells of the subtype indicated per individual and brain region (AG, HC, MT, PFC: n = 48; TH: n = 45; EC: n = 46). The fraction of cells of a subtype was computed relative to all the cells isolated from a brain region of an individual. Data are expressed as mean with 95% confidence intervals and individual data points are shown. Lower panels: difference between group means with 95% confidence intervals (ordinary one-way ANOVA corrected for multiple comparisons using Bonferroni's multiple comparisons test). **e,** Overlap (one-sided Fisher's exact test) between the top 200 genes associated with the gene expression programs indicated (rows) and the inhibitory neuron marker genes (columns). The P values have been adjusted for multiple hypothesis testing; −log10(Bonferroni-corrected P values) are shown. **f,** Overlap (one-sided Fisher's exact test) between inhibitory neuron subtype marker genes and marker genes of excitatory neurons. The P values have been adjusted for multiple hypothesis testing; −log10(Bonferroni-corrected P values) are shown. **g-h,** Linear regression analysis showing a negative association between the glutamatergic- and the GABAergic module score across (**g**) excitatory neuron subtypes and (**h**) inhibitory neuron subtypes. The two confidence bands surrounding the best-fit line, with the area between them shaded green, define the 95% confidence interval of the best-fit line. **i,** The first principal component that was determined based on the average glutamatergic- and GABAergic module scores of all neuronal cell types separates excitatory and inhibitory neuron subtypes. The density plot shows the distribution of the PC1 scores (the coordinates of the individual observations on the first principal component axis) of excitatory and inhibitory neuron subtypes. **j,** Statistical analysis of the distribution of PC1 scores described in (**i**). The box plot shows the distribution of the mean PC1 score per cell type and study participant (n=48 individuals). Within each box, horizontal lines denote median values; boxes extend from the 25th to the 75th percentile of each group's distribution of values; whiskers extend from the 5th to the 95th percentile. ****P < 0.0001 (ordinary one-way ANOVA corrected for multiple comparisons using Šídák's multiple comparisons test). **k,** Ridgeline plot showing the distribution of the PC1 scores described in (**i**) for each neuronal cell type.

UMAP visualization of inhibitory neurons based on integrated analysis of five transcriptomic (scRNA-seq and snRNA-seq) datasets - cells and nuclei are coloured by dataset (a-e)

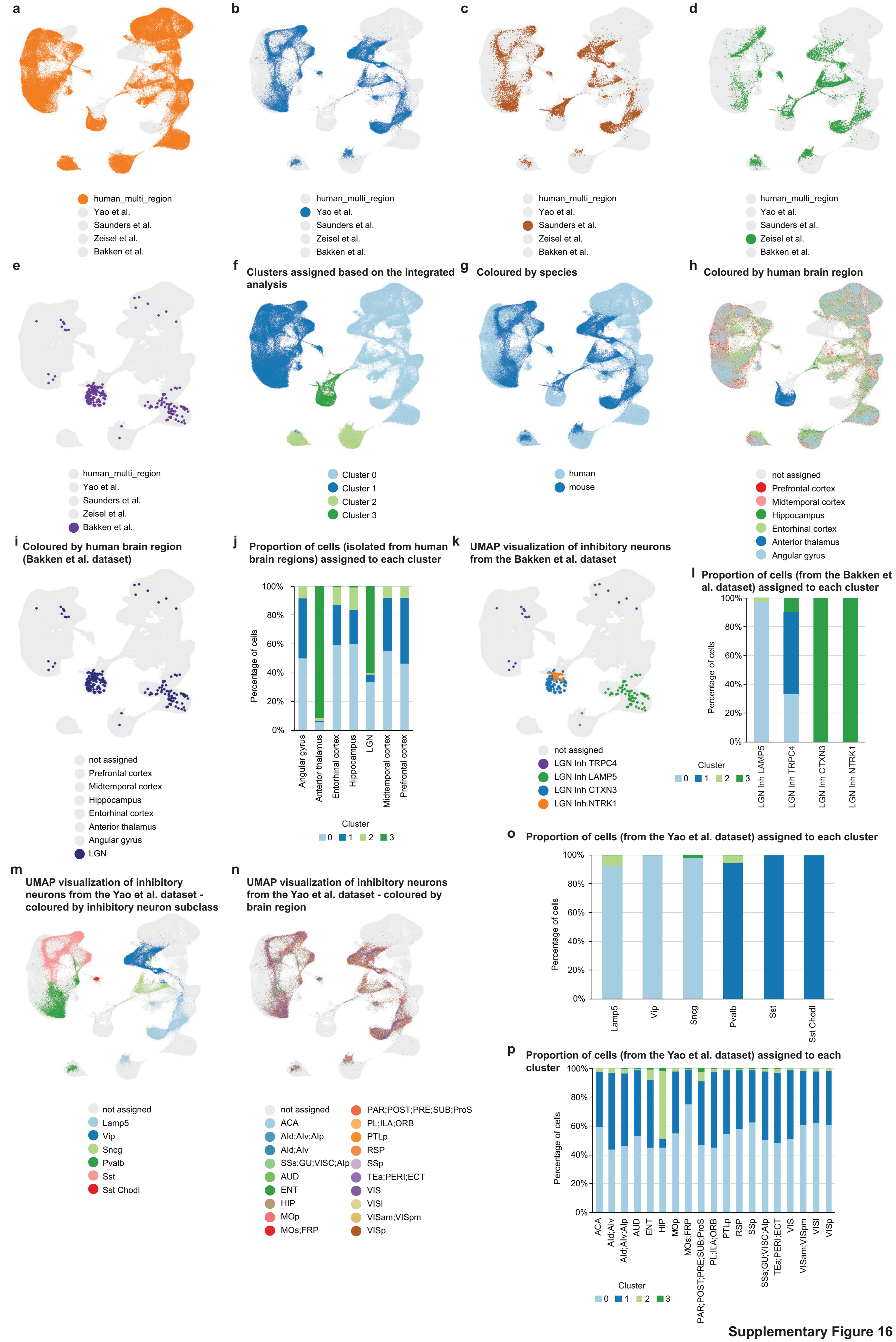

Supplementary Figure 16

Supplementary Figure 16. Aligning human and mouse inhibitory neurons. a-i, UMAP visualization of inhibitory neurons based on integrated analysis of five transcriptomic (scRNA-seq and snRNA-seq) datasets. Cells and nuclei are coloured by dataset (**a-e**), the clusters assigned based on the integrated analysis (**f**), species (**g**), and human brain region (**h,i**). LGN (dorsal lateral geniculate nucleus). **j,** Cluster composition. Bar plot showing the proportion of cells (y axis) from each human brain region (x axis) assigned to each of the four clusters identified based on the integrated analysis. **k,** UMAP visualization of inhibitory neurons from the Bakken et al. dataset coloured by the inhibitory neuron subtypes defined by Bakken et al. **l,** Cluster composition. Bar plot showing the proportion of cells (y axis) of each inhibitory neuron subtype defined by Bakken et al. assigned to each of the four clusters identified based on the integrated analysis. **m-p,** Cluster composition (**o,p**) and UMAP visualization of inhibitory neurons (**m,n**) from the Yao et al. dataset (**m-p**). Cells are coloured by the brain regions or inhibitory neuron subclass indicated. Bar plots show the proportion of cells (y axis) from each brain region or inhibitory neuron subclass (x axis) assigned to each of the four clusters identified based on the integrated analysis. Cortex (CTX) areas included in the Yao et al. dataset are: visual (VIS), frontal pole (FRP), primary motor (MOp), secondary motor (MOs), primary somatosensory (SSp), supple-mental somatosensory (SSs), gustatory (GU), visceral (VISC), auditory (AUD), primary visual (VISp), anteromedial visual (VISam), lateral visual (VISl), posteromedial (VISpm), anterior cingulate (ACA), prelimbic (PL), infra-limbic (ILA), orbital (ORB), agranular insular (AI) (divided into dorsal (AId), ventral (AIv), and posterior (AIp) parts), retrosplenial (RSP), posterior parietal association (PTLp), temporal association (TEa), perirhinal (PERI), and ectorhinal (ECT) areas. Covered regions in HPF (hippocampal formation) are divided into two main parts: the hippocampal region (HIP), including fields CA1, CA2, CA3, and dentate gyrus (DG), and the retrohippocampal region (RHP), including lateral entorhinal area (ENTl), medial entorhinal area (ENTm), parasubiculum (PAR), postsubiculum (POST), presubiculum (PRE), subiculum (SUB), and prosubiculum (ProS).

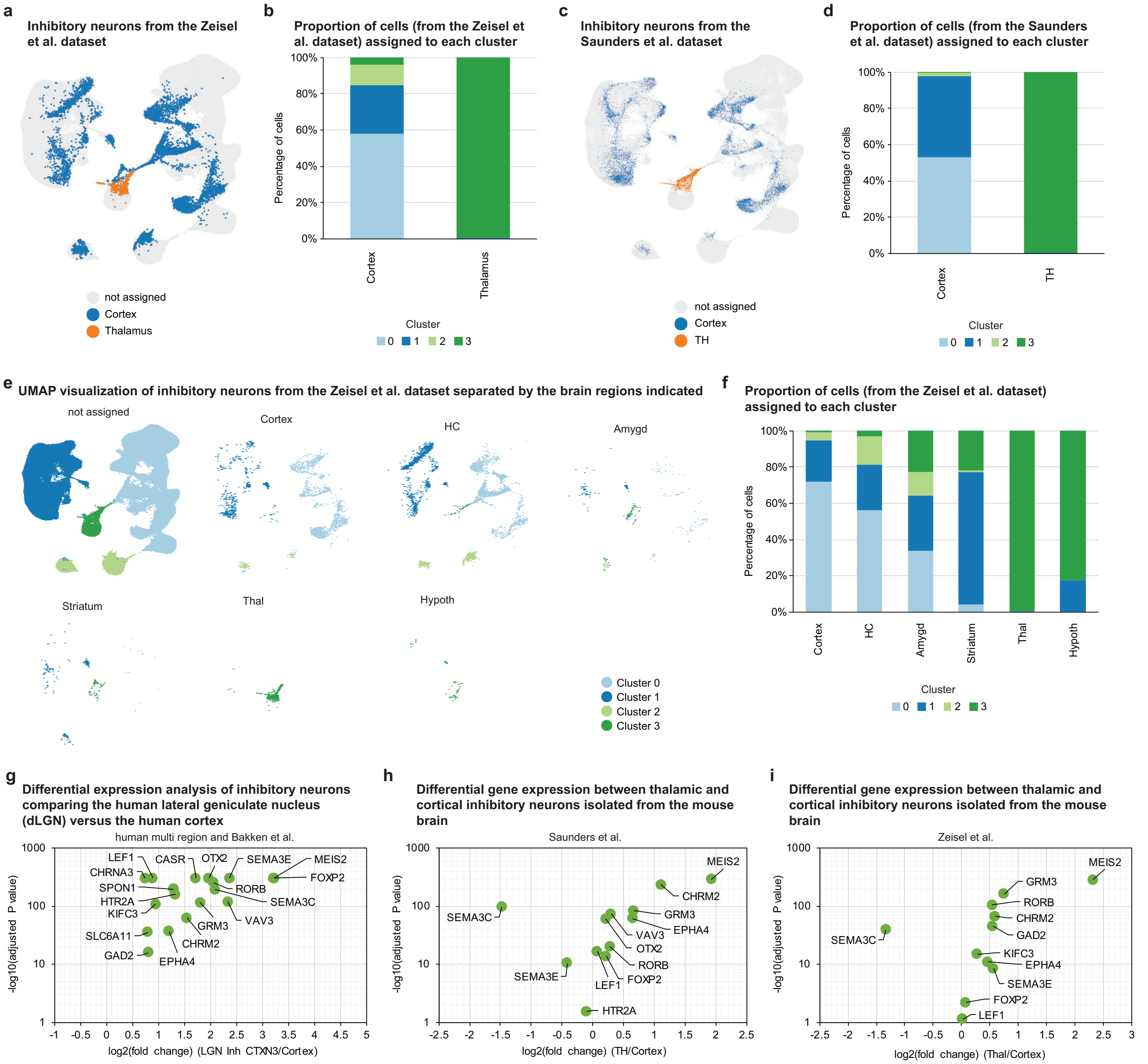

Supplementary Figure 17

Supplementary Figure 17. Aligning human and mouse inhibitory neurons (continued). a-d, Cluster composition (**b,d**) and UMAP visualization of inhibitory neurons (**a,c**) from the Zeisel et al. dataset (**a,b**), and the Saunders et al. dataset (**c,d**). Cells are coloured by the brain regions or inhibitory neuron subclass indicated. Bar plots show the proportion of cells (y axis) from each brain region or inhibitory neuron subclass (x axis) assigned to each of the four clusters identified based on the integrated analysis. TH (thalamus) (**c,d**). **e,** UMAP visualization of inhibitory neurons from the Zeisel et al. dataset separated by the brain regions indicated. Cells are coloured by the clusters assigned based on the integrated analysis. HC (Hippocampus), Amygd (Amygdala), OB (Olfactory bulb), CB (Cerebellum), Thal (Thalamus), Hypoth (Hypothalamus), MB (Midbrain), SC (Spinal Cord). **f,** Cluster composition of the inhibitory neurons from the Zeisel et al. dataset. Bar plot showing the proportion of cells (y axis) from each brain region (x axis) assigned to each of the four clusters identified based on the integrated analysis. **g-i,** Volcano plots of differential expression analysis of inhibitory neurons comparing the human lateral geniculate nucleus (dLGN) versus the human cortex (**g**), and comparing the mouse thalamus versus the mouse cortex based on the Saunders et al. dataset (**h**) and the Zeisel et al. dataset (**i**). P values were derived using the Wilcoxon rank-sum test, with adjustment for multiple comparisons via the Bonferroni method, as implemented in Seurat's FindMarkers function.

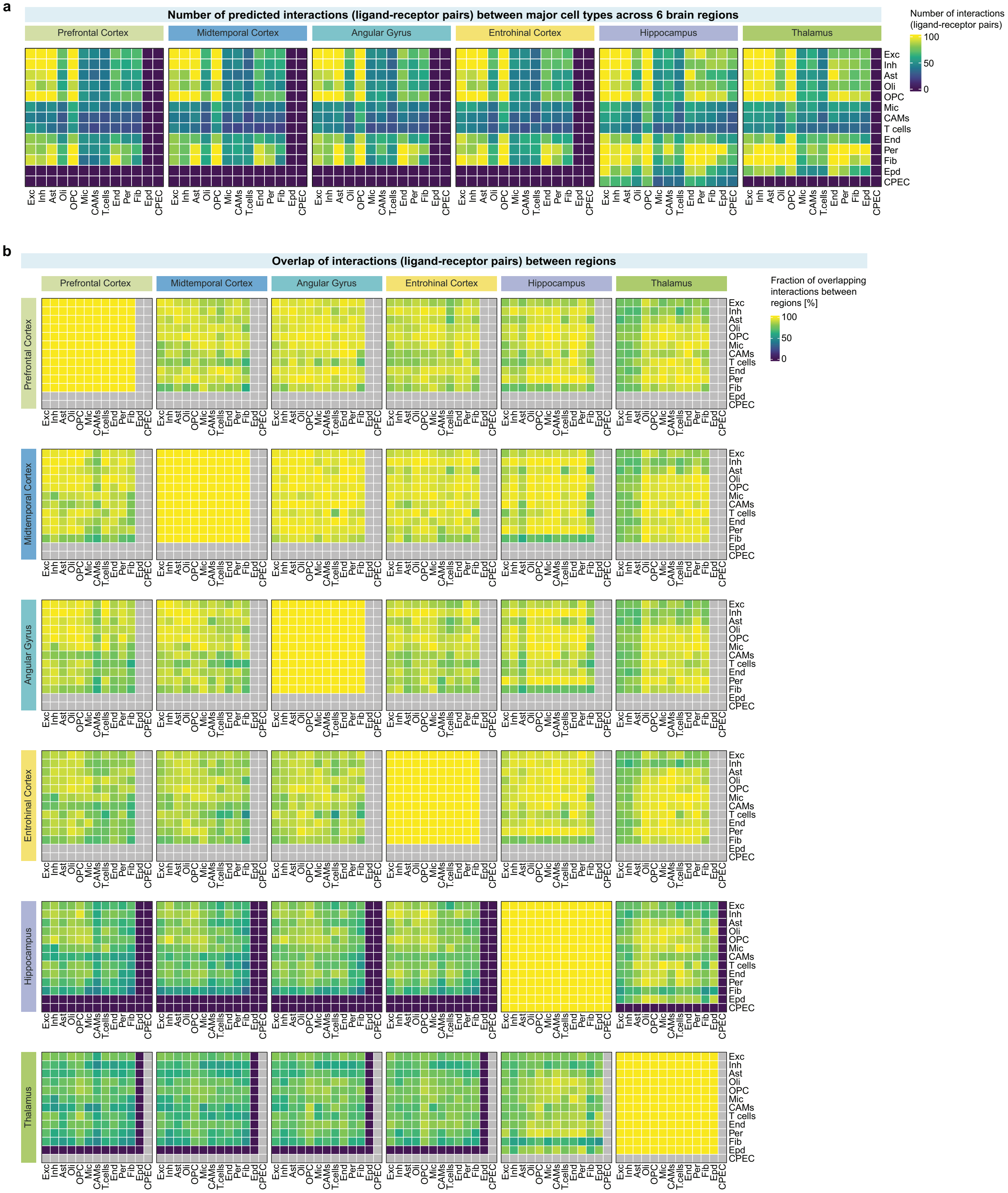

Supplementary Figure 18

Supplementary Figure 18. Predicted cell-cell communication interactions and overlap. a, Number of predicted interactions (ligand-receptor pairs) between major cell types across the 6 brain regions indicated. The cell types expressing the ligands are indicated in the rows and the cell types expressing the receptors are indicated in the columns. **b,** Overlap of interactions (ligand-receptor pairs) between regions. Heatmaps show the percentage of interactions in the brain region shown in the rows that overlap with the interactions in the brain region shown in the columns. In each individual heatmap, the cell types expressing the ligands are indicated in the rows and the cell types expressing the receptors are indicated in the columns.

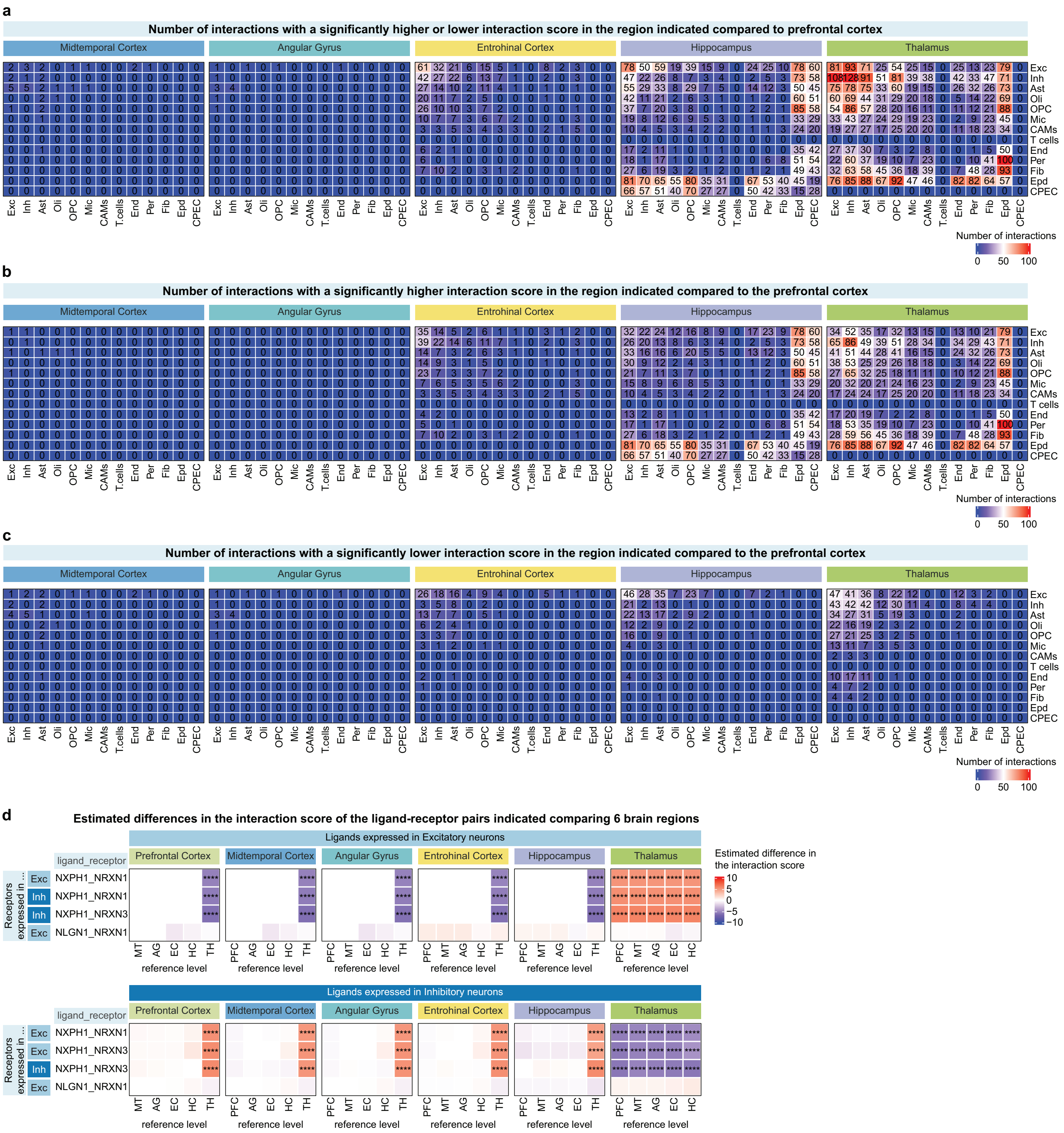

Supplementary Figure 19

Supplementary Figure 19. Region-specific cell-cell communication. a-c, Number of interactions (ligand-receptor pairs) with a significantly higher (**b**) or lower (**c**) interaction score in the region indicated compared to the prefrontal cortex. Panel **a** depicts the total count of interactions exhibiting a significantly higher or lower interaction score. The cell types expressing the ligands are indicated in the rows and the cell types expressing the receptors are indicated in the columns. **d,** Heatmap depicting the results of a linear mixed effects model analysis assessing differences in the interaction score of the indicated ligand-receptor pairs, comparing 6 different brain regions. The heatmap represents the estimated differences in the interaction score comparing the brain regions indicated (the brain region of interest [indicated on the top of each heatmap] was compared to the brain region indicated as the reference level). Top: Ligands expressed in excitatory neurons; bottom: ligands expressed in inhibitory neurons. The cell types expressing the receptors are indicated in the rows. ****P < 0.0001, ***P < 0.001, **P < 0.01, *P < 0.05 (P values for linear mixed models were derived using the "lmerTest" R package and adjusted for multiple testing using the Bonferroni method).

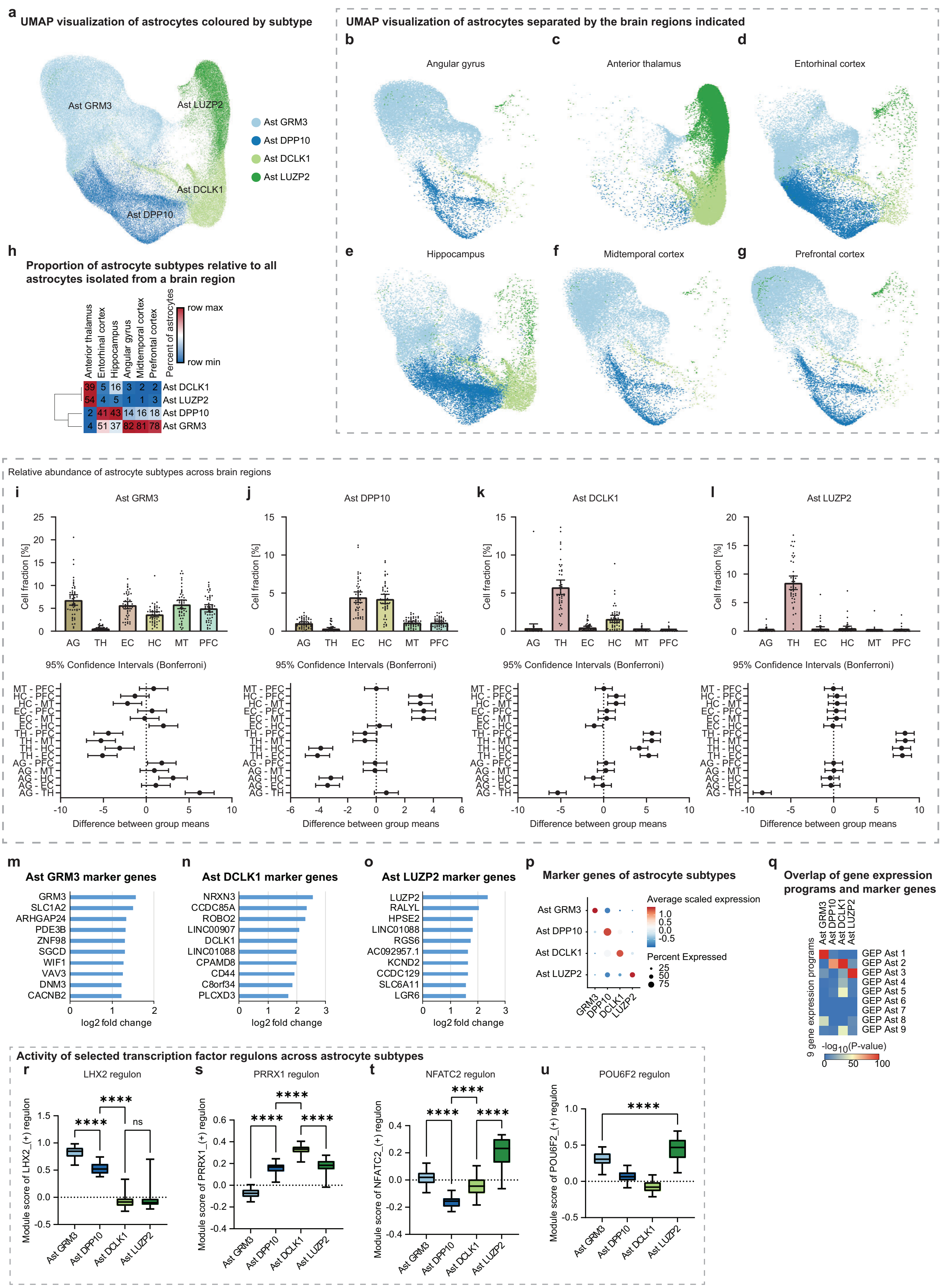

Supplementary Figure 20

Supplementary Figure 20. Astrocyte diversity across different regions of the human brain. a, UMAP visualization of astrocytes coloured by subtype. Cells isolated from all six regions are shown. **b-g,** UMAP visualization of astrocytes separated by the brain regions indicated. **h,** Relative abundance of astrocyte subtypes across brain regions. The heat map shows the proportion of each astrocyte subtype relative to all astrocytes isolated from a brain region. **i-l,** Relative abundance of astrocyte subtypes across brain regions. The bar plots show the mean fraction of cells of the subtype indicated per individual and brain region (AG, HC, MT, PFC: n = 48; TH: n = 45; EC: n = 46). The fraction of cells of an astrocyte subtype was computed relative to all the cells isolated from a brain region of an individual. Data are expressed as mean with 95% confidence intervals and individual data points are shown. Lower panels: difference between group means with 95% confidence intervals (ordinary one-way ANOVA corrected for multiple comparisons using Bonferroni's multiple comparisons test). **m-o,** Top 10 marker genes of the astrocyte subtypes Ast GRM3 (**m**), Ast DCLK1 (**n**), and Ast LUZP2 (**o**). The bar plots show the $\log_2$ fold change in expression between the astrocyte subtype indicated and all other astrocyte subtypes. **p,** Marker genes of astrocyte subtypes. The dot plot shows the average scaled expression of the marker genes indicated across all astrocyte subtypes. **q,** Overlap (one-sided Fisher's exact test) between the top 200 genes associated with the gene expression programs indicated (rows) and the astrocyte marker genes (columns). The P values have been adjusted for multiple hypothesis testing; −log10(Bonferroni-corrected P values) are shown. **r-u,** Activity of selected transcription factor regulons across the astrocyte subtypes. The box plots show the distribution of the average transcription factor regulon module score per individual and astrocyte subtype (n=48 individuals). Within each box, horizontal lines denote median values; boxes extend from the 25th to the 75th percentile of each group's distribution of values; whiskers extend from the 5th to the 95th percentile. ****P < 0.0001; ns, P > 0.05 (ordinary one-way ANOVA corrected for multiple comparisons using Šídák's multiple comparisons test).

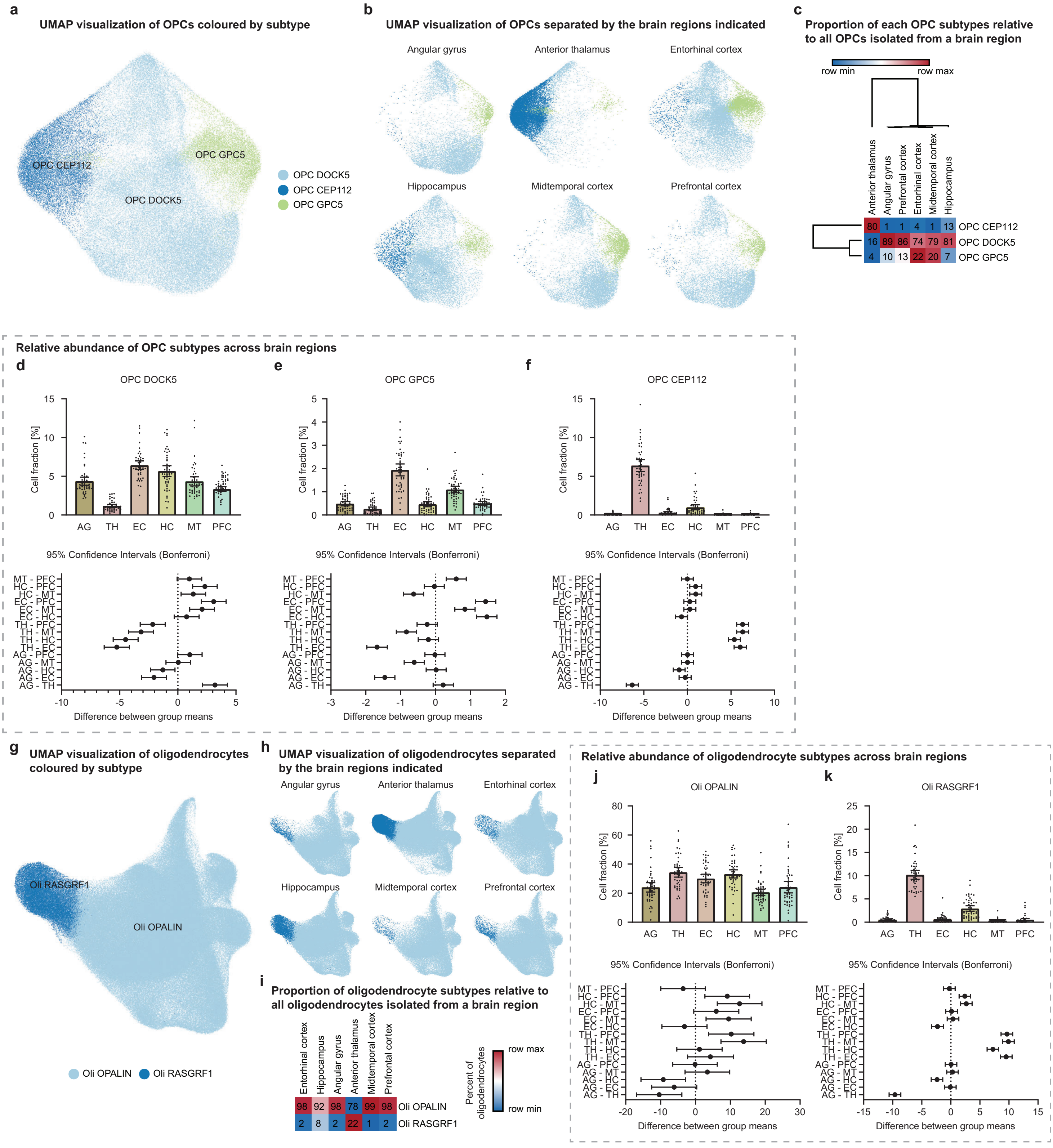

Supplementary Figure 21

Supplementary Figure 21. Oligodendrocyte lineage diversity across different regions of the human brain. a, UMAP visualization of OPCs coloured by subtype. Cells isolated from all six regions are shown. **b,** UMAP visualization of OPCs separated by the brain regions indicated. **c,** Relative abundance of OPC subtypes across brain regions. The heat map shows the proportion of each OPC subtype relative to all OPCs isolated from a brain region. **d-f,** Relative abundance of OPC subtypes across brain regions. The bar plots show the mean fraction of cells of the cell types indicated per individual and brain region (AG, HC, MT, PFC: n = 48; TH: n = 45; EC: n = 46). The fraction of cells of a cell type was computed relative to all the cells isolated from a brain region of an individual. Data are expressed as mean with 95% confidence intervals and individual data points are shown. Lower panels: difference between group means with 95% confidence intervals (ordinary one-way ANOVA corrected for multiple comparisons using Bonferroni's multiple comparisons test). **g,** UMAP visualization of oligodendrocytes coloured by subtype. Cells isolated from all six regions are shown. **h,** UMAP visualization of oligodendrocytes separated by the brain regions indicated. **i,** Relative abundance of oligodendrocyte subtypes across brain regions. The heat map shows the proportion of each oligodendrocyte subtype relative to all oligodendrocytes isolated from a brain region. **j,k,** Relative abundance of oligodendrocyte subtypes across brain regions. The bar plots show the mean fraction of cells of the cell types indicated per individual and brain region (AG, HC, MT, PFC: n = 48; TH: n = 45; EC: n = 46). The fraction of cells of a cell type was computed relative to all the cells isolated from a brain region of an individual. Data are expressed as mean with 95% confidence intervals and individual data points are shown. Lower panels: difference between group means with 95% confidence intervals (ordinary one-way ANOVA corrected for multiple comparisons using Bonferroni's multiple comparisons test).

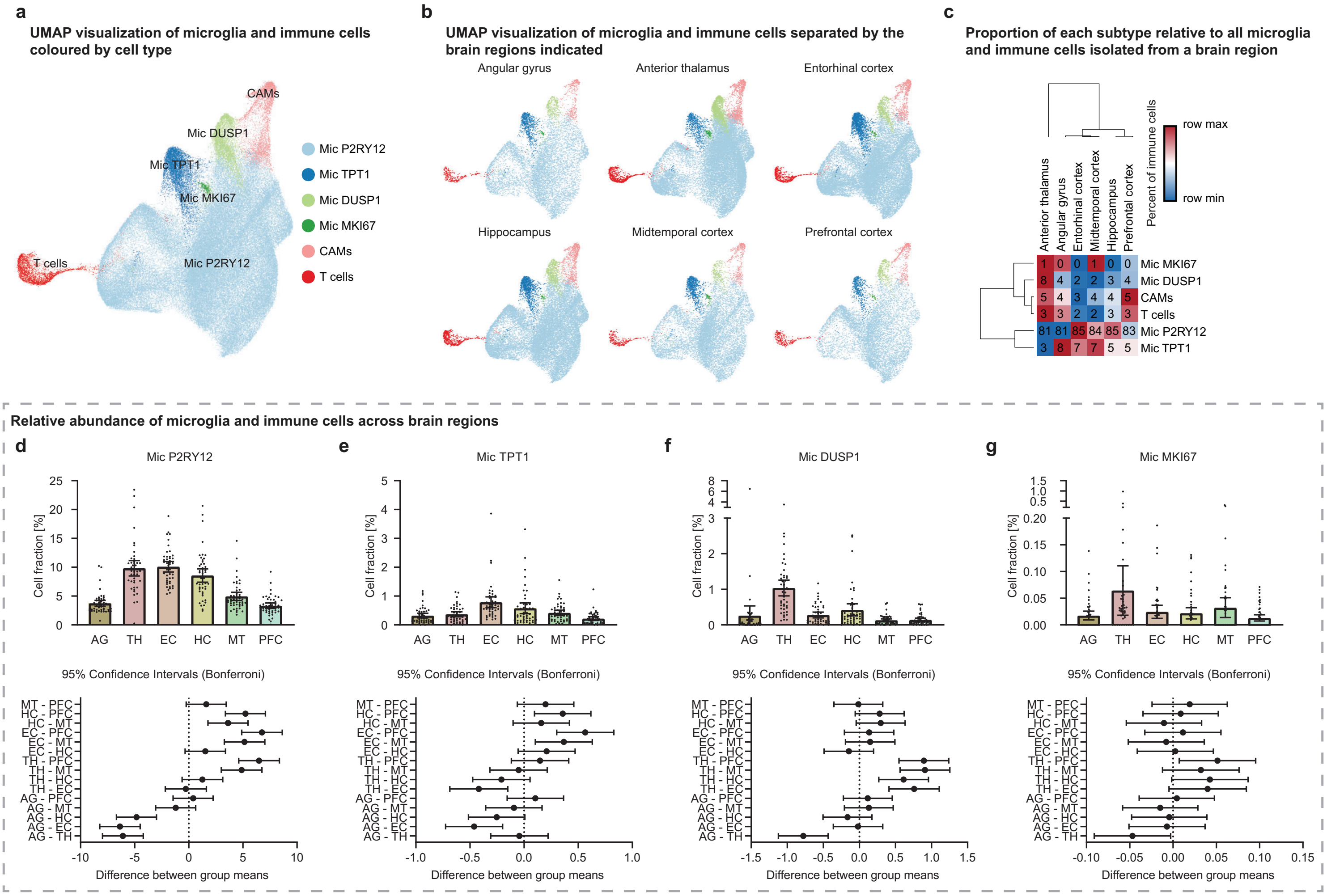

Supplementary Figure 22

Supplementary Figure 22: Microglia and immune cell diversity across brain regions. a, UMAP visualization of microglia and immune cells coloured by cell type. Cells isolated from all six regions are shown. **b,** UMAP visualization of microglia and immune cells separated by the brain regions indicated. **c,** Relative abundance of microglia and immune cells across brain regions. The heat map shows the proportion of each subtype relative to all microglia and immune cells isolated from a brain region. **d-g,** Relative abundance of microglia and immune cells across brain regions. The bar plots show the mean fraction of cells of the subtypes indicated per individual and brain region (AG, HC, MT, PFC: n = 48; TH: n = 45; EC: n = 46). The fraction of cells of a subtype was computed relative to all the cells isolated from a brain region of an individual. Data are expressed as mean with 95% confidence intervals and individual data points are shown. Lower panels: difference between group means with 95% confidence intervals (ordinary one-way ANOVA corrected for multiple comparisons using Bonferroni's multiple comparisons test).

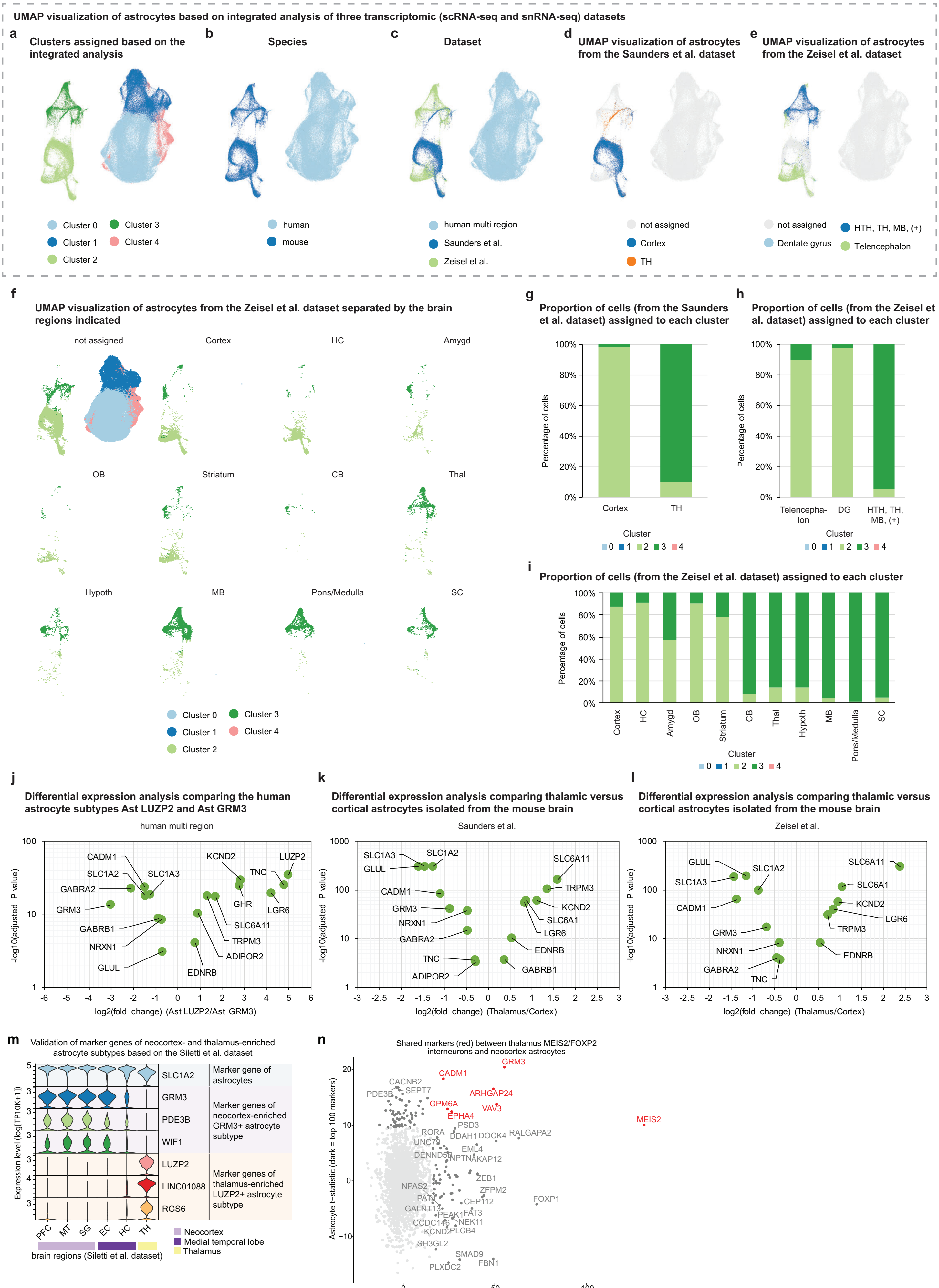

Supplementary Figure 23

Supplementary Figure 23. Aligning human and mouse astrocytes. a-f, UMAP visualization of astrocytes based on integrated analysis of three transcriptomic (scRNA-seq and snRNA-seq) datasets. Cells and nuclei are coloured by the clusters assigned based on the integrated analysis (**a**), species (**b**), and dataset (**c**). **d,e,** UMAP visualization of astrocytes from the Saunders et al. dataset (**d**) and the Zeisel et al. dataset (**e**). Cells are coloured by the brain region indicated. TH (thalamus), HTH, TH, MB, (+) (Hypothalamus, Thalamus, Midbrain dorsal, Midbrain ventral, Pons, Medulla, Spinal cord). **f,** UMAP visualization of astrocytes from the Zeisel et al. dataset separated by the brain regions indicated. Cells are coloured by the clusters assigned based on the integrated analysis. HC (Hippocampus), Amygd (Amygdala), OB (Olfactory bulb), CB (Cerebellum), Thal (Thalamus), Hypoth (Hypothalamus), MB (Midbrain), SC (Spinal Cord). **g-i,** Cluster composition. Bar plot showing the proportion of astrocytes (y axis) from each brain region (x axis) assigned to each of the five clusters identified based on the integrated analysis. The panels show the results for the Saunders et al. dataset (**g**) and the Zeisel et al. dataset (**h,i**). **j-l,** Volcano plots of differential expression analyses comparing the human astrocyte subtypes Ast LUZP2 and Ast GRM3 (**j**), and comparing thalamic versus cortical astrocytes isolated from the mouse brain based on the Saunders et al. dataset (**k**) and the Zeisel et al. dataset (**l**). P values were derived using the Wilcoxon rank-sum test, with adjustment for multiple comparisons via the Bonferroni method, as implemented in Seurat's FindMarkers function. **m,** Validation of marker genes of neocortex- and thalamus-enriched astrocyte subtypes using the Siletti et al. dataset. The violin plots depict the expression levels of astrocyte subtype marker genes identified in our study within astrocytes from the brain regions indicated in the Siletti et al. dataset[14]. **n,** Shared markers between neocortex astrocytes and thalamus interneurons (Inh MEIS2 FOXP2). Scatter plot of t-test for each subtype against other cells of the same class (vs. astrocytes and vs. inhibitory neurons), with top 100 genes colored in gray and overlap colored in red.

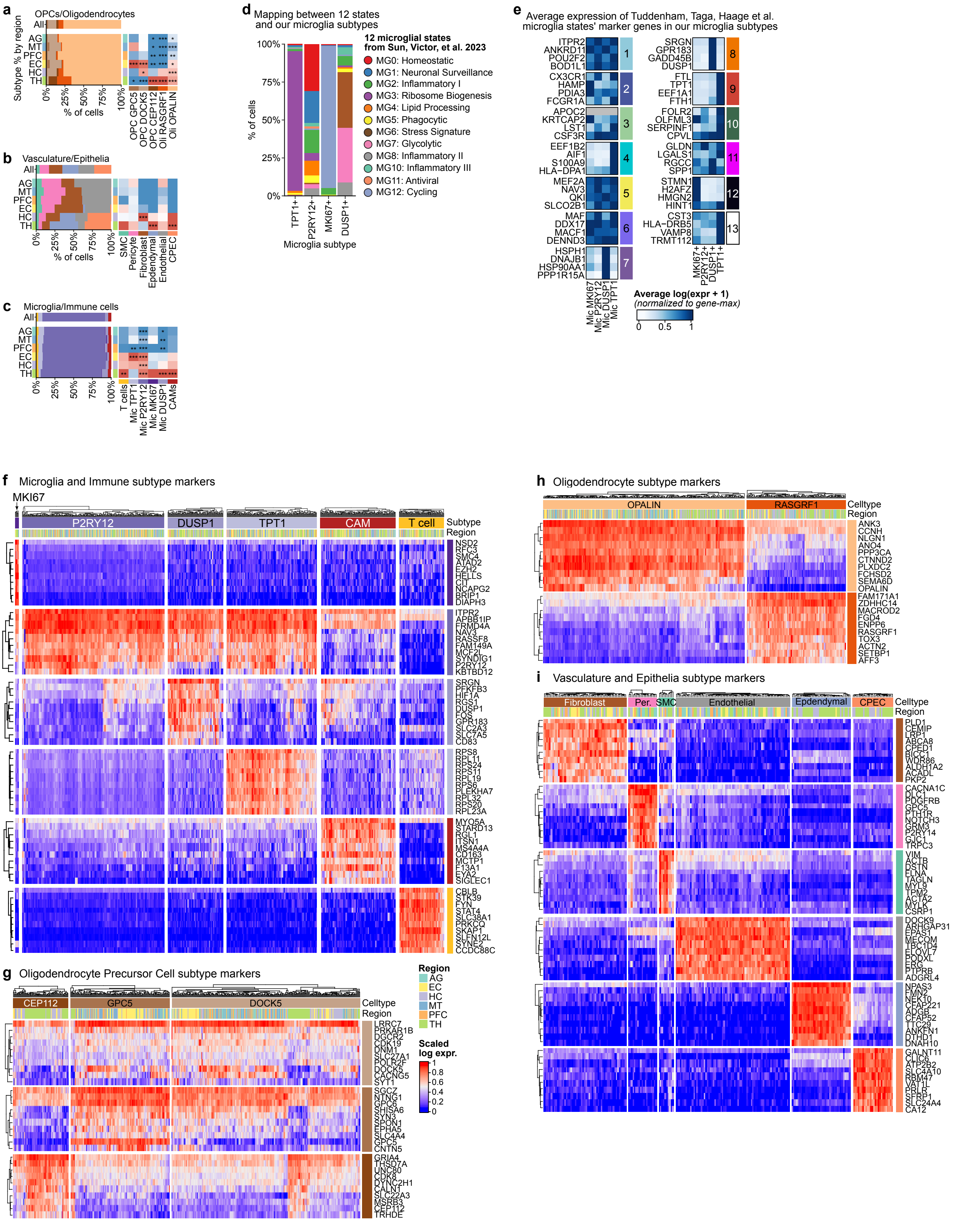

Supplementary Figure 24

Supplementary Figure 24. Non-neuronal subtype marker gene heatmaps. a-c, Regional and overall composition of oligodendroglial (**a**), vascular (**b**), and immune (**c**) cell types. **d,** Percentage of each of our four microglia subtypes mapping to each of the twelve subtypes from Sun, Victor, et al. 2023[120]. **e,** Average expression of the microglia subtype marker genes from Tuddenham, Taga, Haage et al. 2022 in our microglia subtypes[121]. **f-i,** Marker gene heatmaps for microglia and immune cell subtypes (**f**), oligodendrocyte precursor cell subtypes (**g**), oligodendrocyte subtypes (**h**), and vasculature and epithelia subtypes (**i**). Heatmap shows marker gene (rows) average expression in pseudo-bulk samples at the subtype level (columns). Expression is row-normalized to the maximum expression in each gene. Top 10 marker genes for each subtype are shown as ranked by the p-value of the subtype's coefficient in a linear regression prediction of gene expression at the pseudo-bulk sample-level.

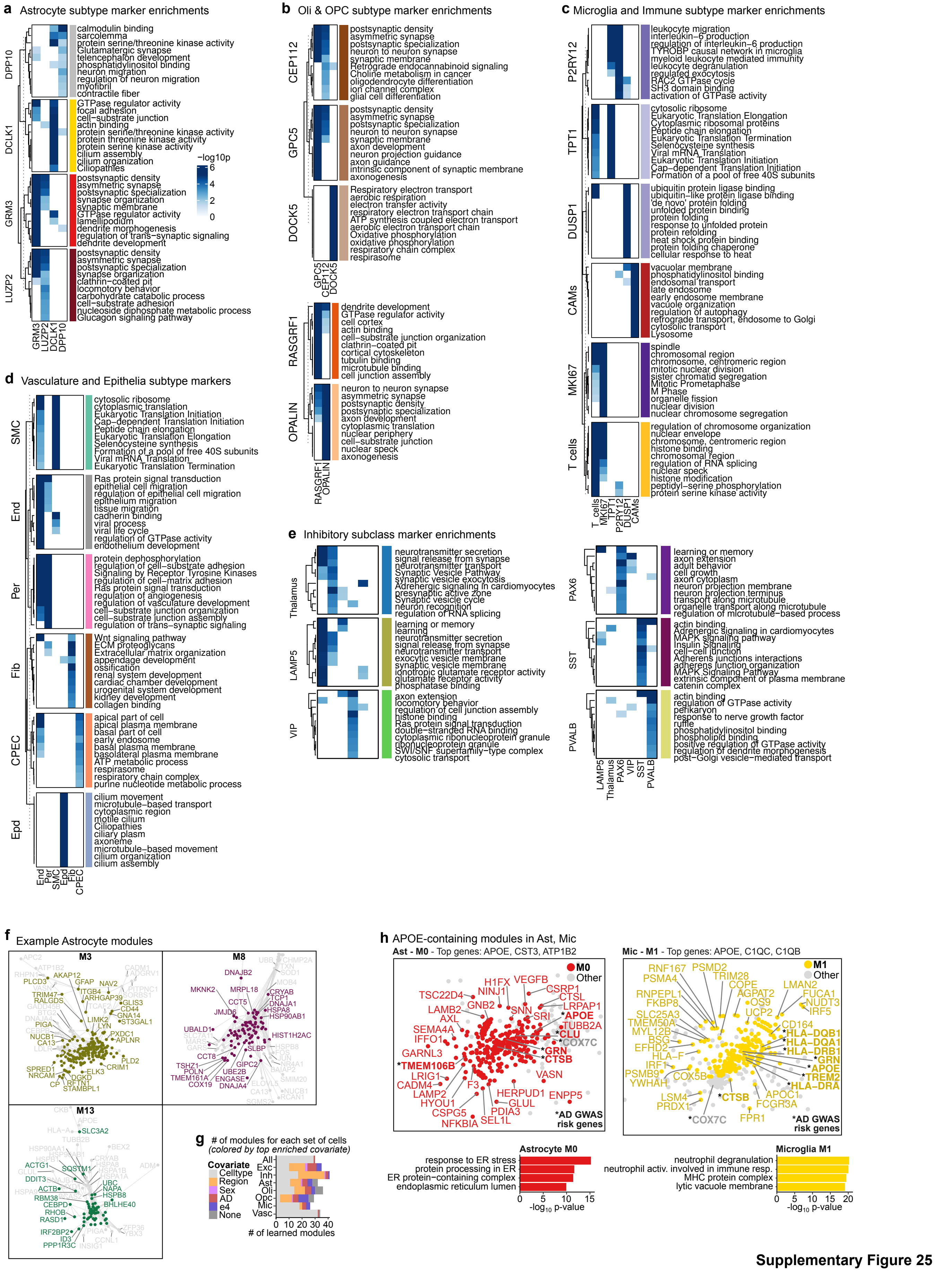

Supplementary Figure 25

Supplementary Figure 25. Marker gene enrichments and modules. a-e, Unique enrichments for marker genes for astrocytes (**a**), oligodendrocytes and OPCs (**b**), microglia and immune cells (**c**), vasculature and epithelia (**d**), and inhibitory subclasses (**e**). Top 10 terms that are unique to two subclasses or fewer and that have fewer than 500 genes in the term are shown. Adjusted p-values are shown as -$\log_{10}$p-values capped at 5. **f,** Example insets for three astrocyte modules. **g,** Number of modules computed for each cell type, colored by their top-associated covariate. **h,** Example insets for two modules containing APOE (Ast M0 and Mic M1) with their top functional enrichments (barplots, below). Starred genes are AD GWAS risk genes.

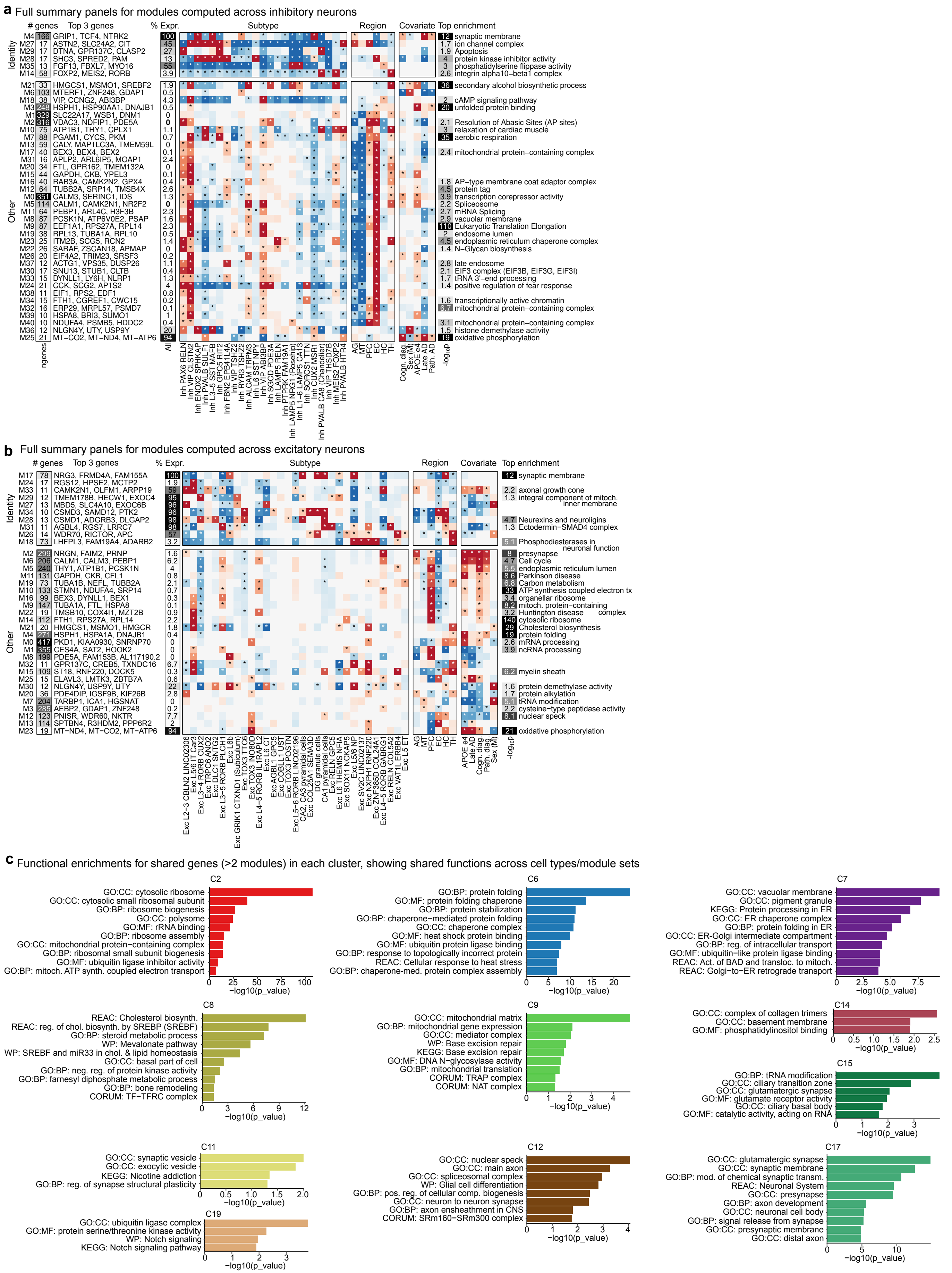

Supplementary Figure 26

Supplementary Figure 26. Module summary panels across modules. a-b, Overview of gene expression modules with at least 10 genes each across inhibitory (**a**) and excitatory neurons (**b**), showing the module name, number of genes, percent expression, top module genes, enrichments by subtype and region, and the top functional enrichment for each module. Percent expression is the percent of cells whose average expression (log1p, normalized) of the module is above 1. Covariate enrichments are performed by hypergeometric test, comparing the intersection of cells with z-scored module expression of at least 1 vs. with $z < 1$ against a particular level of a covariate of interest (e.g. cells from the entorhinal cortex region or cells of a specific subtype). Modules are split into identity vs. other, where identity modules are highly enriched in a single subtype and have an average expression greater than 1 (log1p, normalized) for over 50% of the subtype's cells. **c,** Functional enrichments for clusters of modules for the shared genes (>2 modules) in each cluster (only clusters with significant enrichments shown). Up to 10 enrichments shown, ordered by p-value, labeled by their source and only keeping terms with fewer than 500 genes.

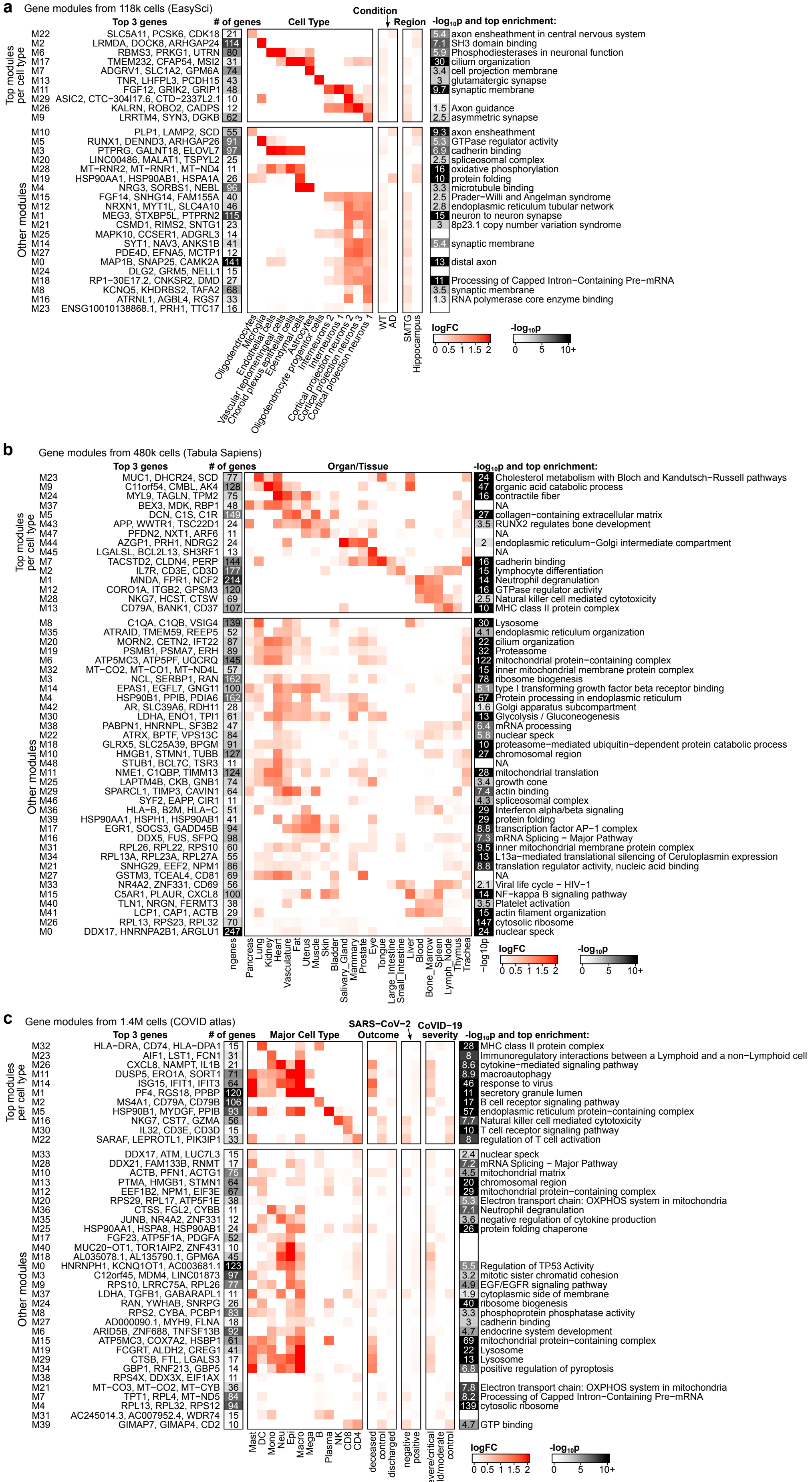

Supplementary Figure 27

Supplementary Figure 27. Module summary panels for external datasets. a-c, Overview of gene expression modules with at least 10 genes across all cells in three datasets: AD brain using EasySci (**a**), 480k Tabula Sapiens (**b**), and an atlas of immune cell response to COVID (**c**), showing number of genes, top module genes, log fold change enrichment against cell-level covariates, and the top functional enrichment for each module.

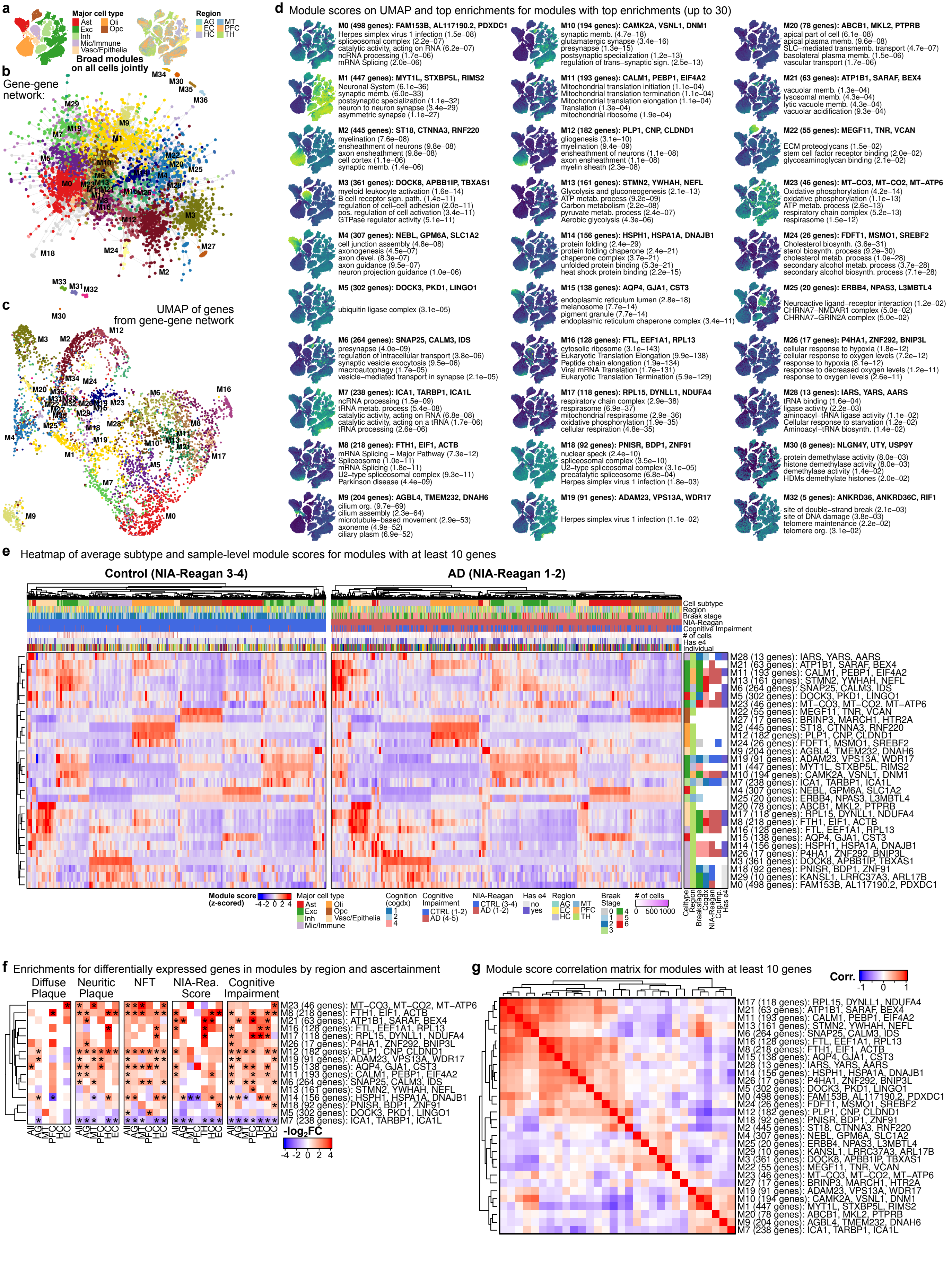

Supplementary Figure 28

Supplementary Figure 28. Broad modules computed using all cells in the atlas. a, UMAP plots of cells used to detect the broad modules across all cells, colored by major cell type and by region of origin. **b,** Gene-gene network from modules method, where each point is a gene, edges connect pairs of genes with above-threshold correlation, and genes are colored by their leiden cluster (module) in the graph. **c,** UMAP of genes from estimated gene-gene correlation from sample-decorrelated matrix. Each point is a gene, colored by the leiden clusters (modules) as in (**b**). **d**, Modules scores on the cell UMAP from (**a**) and the names and p-values of the top gene set enrichments (term size < 500) for the 30 modules with the top enrichments (or top 30 in order of size). Module scores calculated as the average (normalized, log1p) expression of all of the genes in the module. Modules are sorted by number of genes and labeled with the top three genes that are most correlated to the overall module score. **e,** Heatmap of average subtype and sample-level module scores for modules with at least 10 genes. Module scores are z-scored for the heatmap. Top panel annotates columns, giving the properties of each subtype + sample column. Side panel shows hypergeometric enrichments for top z-scoring samples ($z > 1$) in each metadata column. **f,** Heatmap of hypergeometric enrichments of up (red) or down (blue) AD DEGs in modules for DEGs computed under different conditions and brain regions. Only modules with significant enrichments are shown and rows are clustered hierarchically by Euclidean distance. **g,** Correlation matrix heatmap for module scores for modules with at least 10 genes, with rows and columns hierarchically clustered by Euclidean distance.

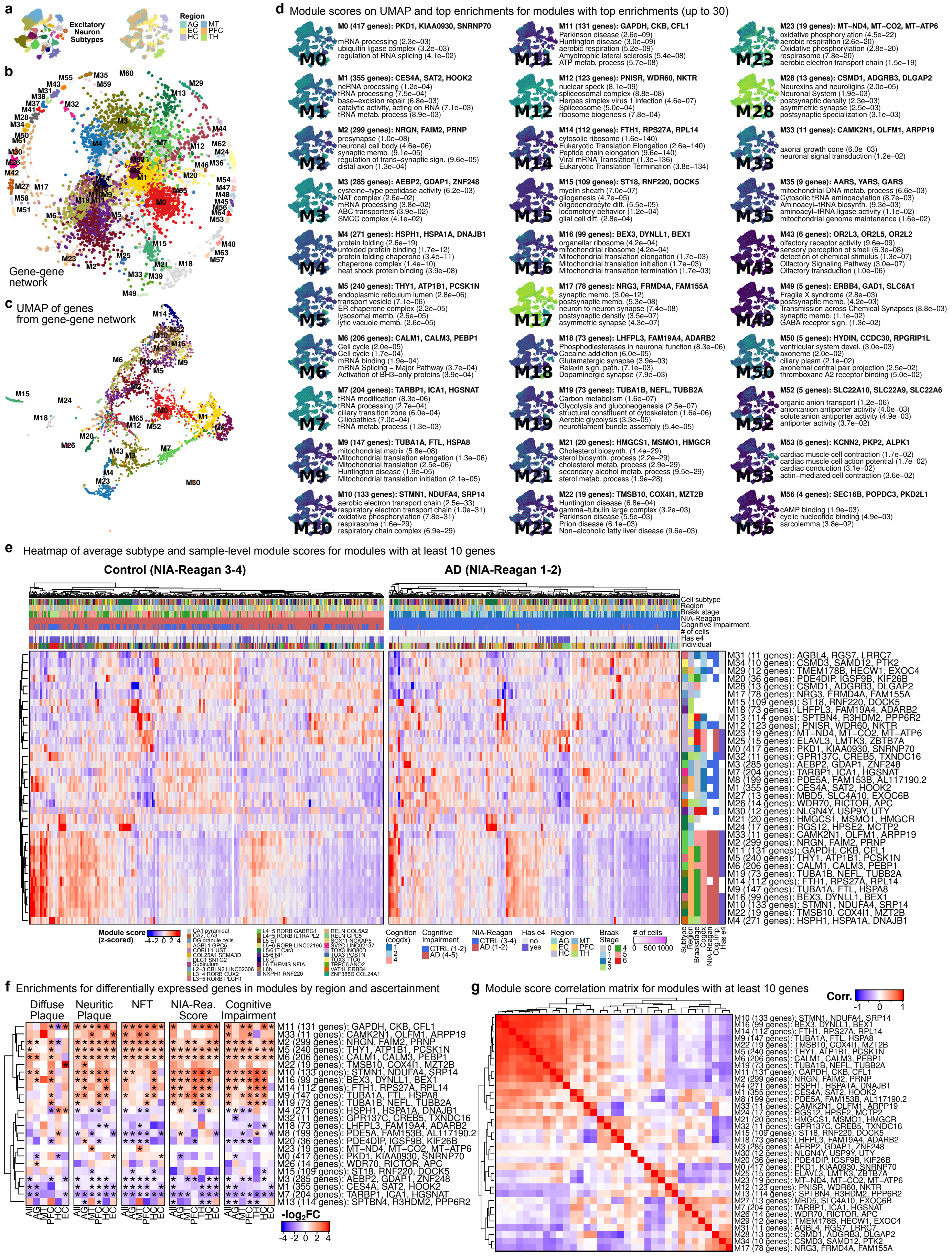

Supplementary Figure 29

Supplementary Figure 29. Excitatory modules. a, UMAP plots of cells used to detect modules in excitatory neurons alone, colored by cell subtype and by region of origin. **b,** Gene-gene network from modules method, where each point is a gene, edges connect pairs of genes with above-threshold correlation, and genes are colored by their leiden cluster (module) in the graph. **c,** UMAP of genes from estimated gene-gene correlation from sample-decorrelated matrix. Each point is a gene, colored by the leiden clusters (modules) as in (**b**). **d**, Modules scores on the cell UMAP from (**a**) and the names and p-values of the top gene set enrichments (term size < 500) for the 30 modules with the top enrichments (or top 30 in order of size). Module scores calculated as the average (normalized, log1p) expression of all of the genes in the module. Modules are sorted by number of genes and labeled with the top three genes that are most correlated to the overall module score. **e,** Heatmap of average subtype and sample-level module scores for modules with at least 10 genes. Module scores are z-scored for the heatmap. Top panel annotates columns, giving the properties of each subtype + sample column. Side panel shows hypergeometric enrichments for top z-scoring samples (z > 1) in each metadata column. **f,** Heatmap of hypergeometric enrichments of up (red) or down (blue) AD DEGs in modules for DEGs computed under different conditions and brain regions. Only modules with significant enrichments are shown and rows are clustered hierarchically by Euclidean distance. **g,** Correlation matrix heatmap for module scores for modules with at least 10 genes, with rows and columns hierarchically clustered by Euclidean distance.

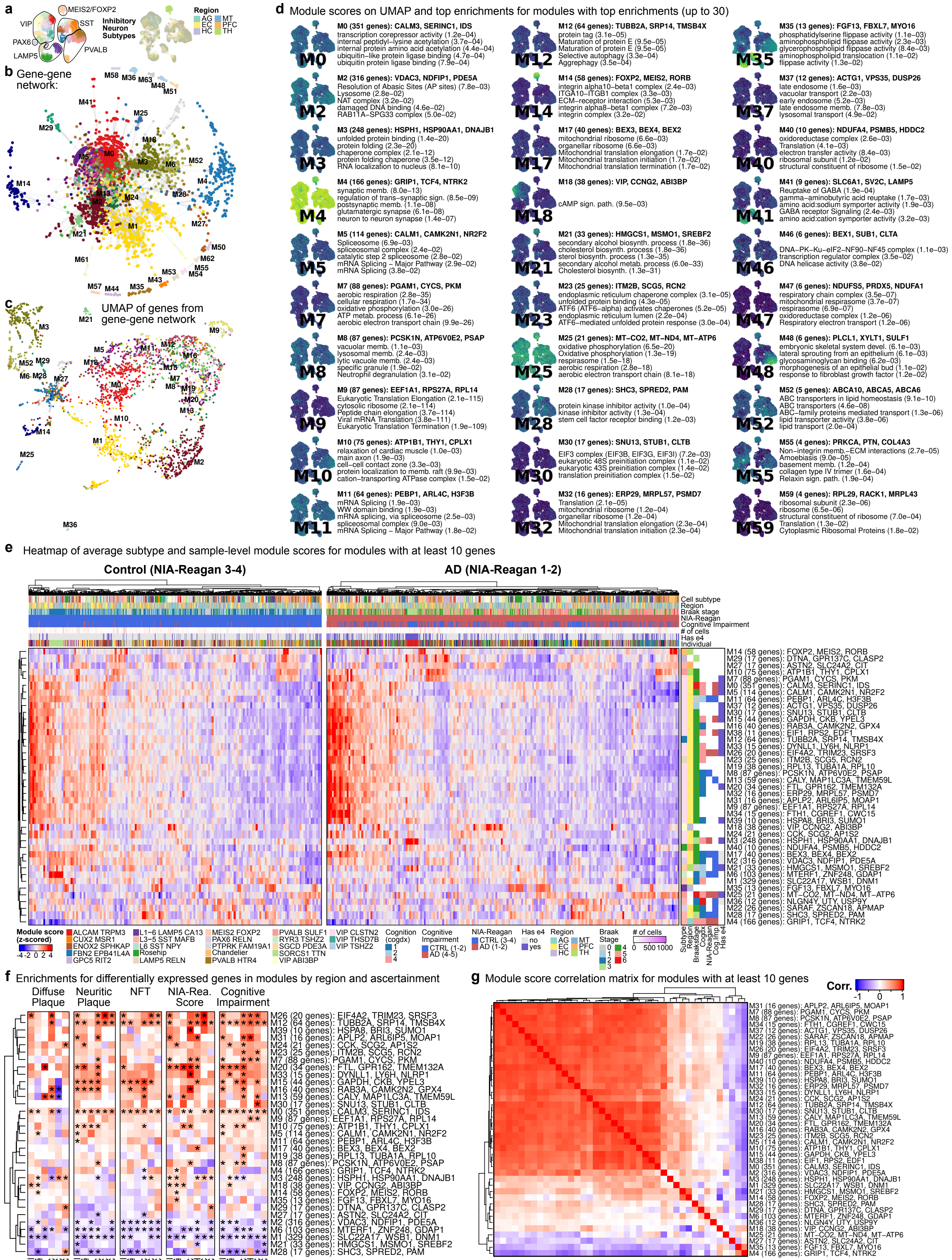

Supplementary Figure 30

Supplementary Figure 30. Inhibitory modules. a, UMAP plots of cells used to detect modules in inhibitory neurons alone, colored by cell subtype and by region of origin. **b,** Gene-gene network from modules method, where each point is a gene, edges connect pairs of genes with above-threshold correlation, and genes are colored by their leiden cluster (module) in the graph. **c,** UMAP of genes from estimated gene-gene correlation from sample-decorrelated matrix. Each point is a gene, colored by the leiden clusters (modules) as in (**b**). **d**, Modules scores on the cell UMAP from (**a**) and the names and p-values of the top gene set enrichments (term size < 500) for the 30 modules with the top enrichments (or top 30 in order of size). Module scores calculated as the average (normalized, log1p) expression of all of the genes in the module. Modules are sorted by number of genes and labeled with the top three genes that are most correlated to the overall module score. **e,** Heatmap of average subtype and sample-level module scores for modules with at least 10 genes. Module scores are z-scored for the heatmap. Top panel annotates columns, giving the properties of each subtype + sample column. Side panel shows hypergeometric enrichments for top z-scoring samples (z > 1) in each metadata column. **f,** Heatmap of hypergeometric enrichments of up (red) or down (blue) AD DEGs in modules for DEGs computed under different conditions and brain regions. Only modules with significant enrichments are shown and rows are clustered hierarchically by Euclidean distance. **g,** Correlation matrix heatmap for module scores for modules with at least 10 genes, with rows and columns hierarchically clustered by Euclidean distance.

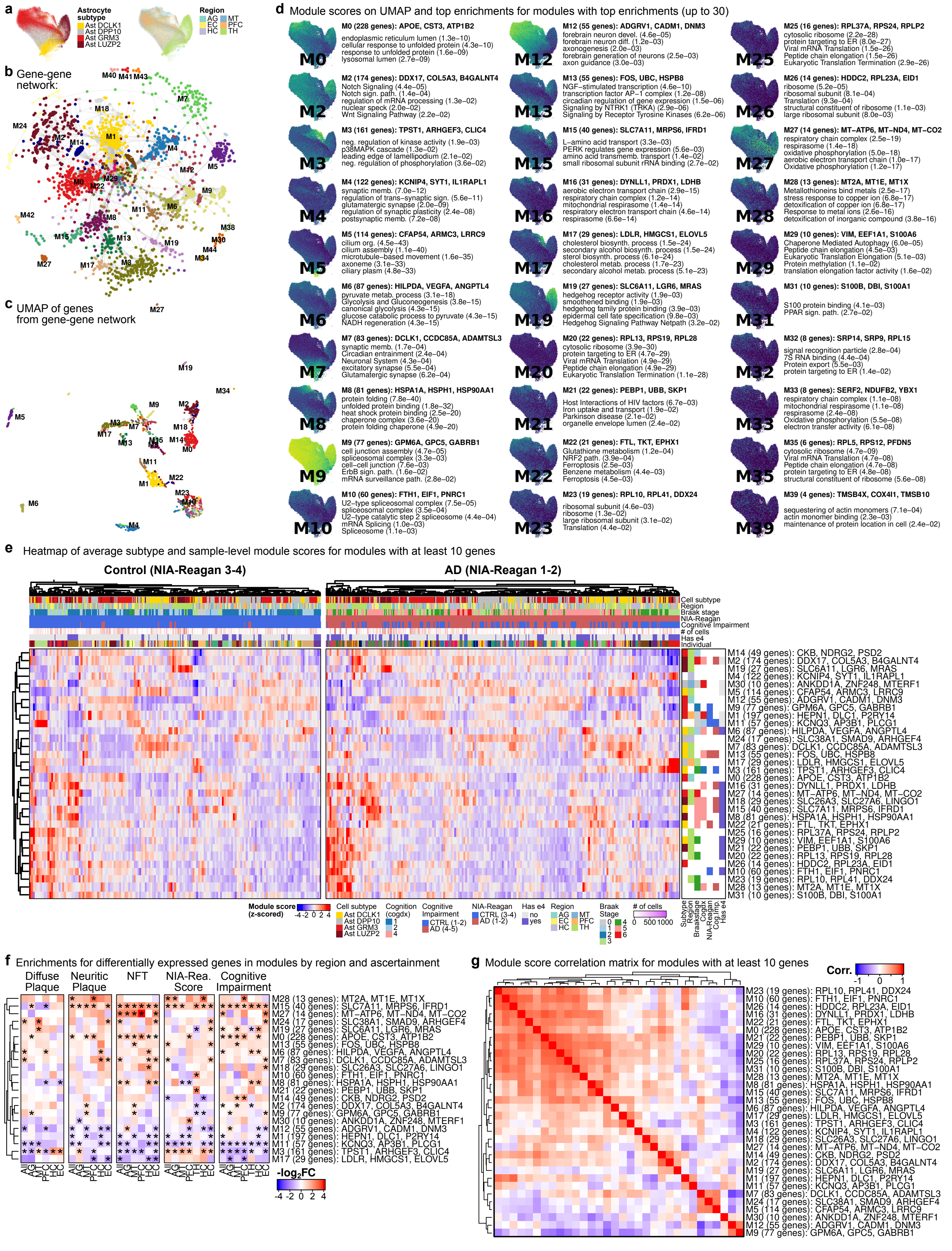

Supplementary Figure 31

Supplementary Figure 31. Astrocyte modules. a, UMAP plots of cells used to detect modules in astrocytes alone, colored by cell subtype and by region of origin. **b,** Gene-gene network from modules method, where each point is a gene, edges connect pairs of genes with above-threshold correlation, and genes are colored by their leiden cluster (module) in the graph. **c,** UMAP of genes from estimated gene-gene correlation from sample-decorrelated matrix. Each point is a gene, colored by the leiden clusters (modules) as in (**b**). **d**, Modules scores on the cell UMAP from (**a**) and the names and p-values of the top gene set enrichments (term size < 500) for the 30 modules with the top enrichments (or top 30 in order of size). Module scores calculated as the average (normalized, log1p) expression of all of the genes in the module. Modules are sorted by number of genes and labeled with the top three genes that are most correlated to the overall module score. **e,** Heatmap of average subtype and sample-level module scores for modules with at least 10 genes. Module scores are z-scored for the heatmap. Top panel annotates columns, giving the properties of each subtype + sample column. Side panel shows hypergeometric enrichments for top z-scoring samples (z > 1) in each metadata column. **f,** Heatmap of hypergeometric enrichments of up (red) or down (blue) AD DEGs in modules for DEGs computed under different conditions and brain regions. Only modules with significant enrichments are shown and rows are clustered hierarchically by Euclidean distance. **g,** Correlation matrix heatmap for module scores for modules with at least 10 genes, with rows and columns hierarchically clustered by Euclidean distance.

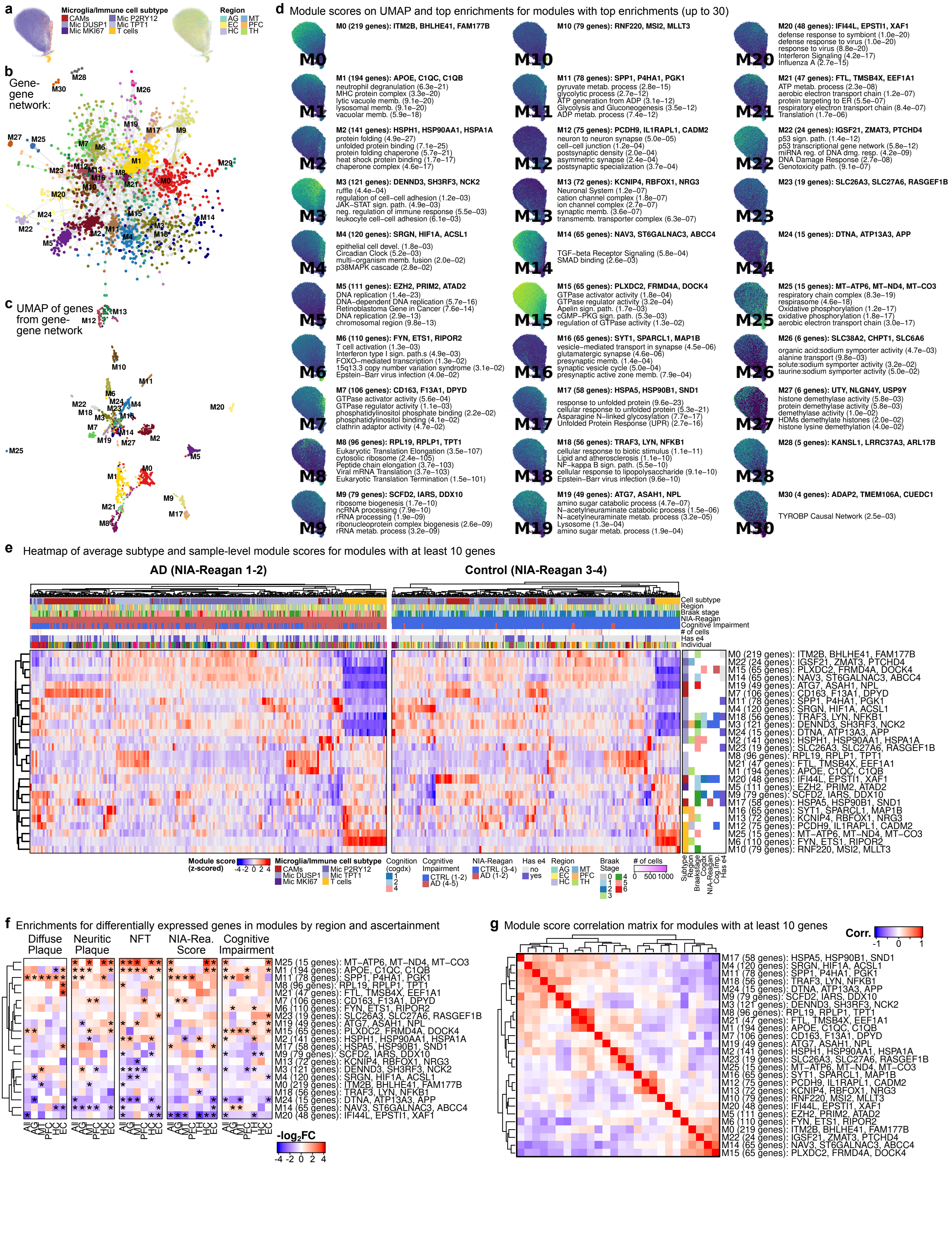

Supplementary Figure 32

Supplementary Figure 32. Microglia/Immune modules. a, UMAP plots of cells used to detect modules in microglia and immune cells alone, colored by cell subtype and by region of origin. **b,** Gene-gene network from modules method, where each point is a gene, edges connect pairs of genes with above-threshold correlation, and genes are colored by their leiden cluster (module) in the graph. **c,** UMAP of genes from estimated gene-gene correlation from sample-decorrelated matrix. Each point is a gene, colored by the leiden clusters (modules) as in (**b**). **d**, Modules scores on the cell UMAP from (**a**) and the names and p-values of the top gene set enrichments (term size < 500) for the 30 modules with the top enrichments (or top 30 in order of size). Module scores calculated as the average (normalized, log1p) expression of all of the genes in the module. Modules are sorted by number of genes and labeled with the top three genes that are most correlated to the overall module score. **e,** Heatmap of average subtype and sample-level module scores for modules with at least 10 genes. Module scores are z-scored for the heatmap. Top panel annotates columns, giving the properties of each subtype + sample column. Side panel shows hypergeometric enrichments for top z-scoring samples (z > 1) in each metadata column. **f,** Heatmap of hypergeometric enrichments of up (red) or down (blue) AD DEGs in modules for DEGs computed under different conditions and brain regions. Only modules with significant enrichments are shown and rows are clustered hierarchically by Euclidean distance. **g,** Correlation matrix heatmap for module scores for modules with at least 10 genes, with rows and columns hierarchically clustered by Euclidean distance.

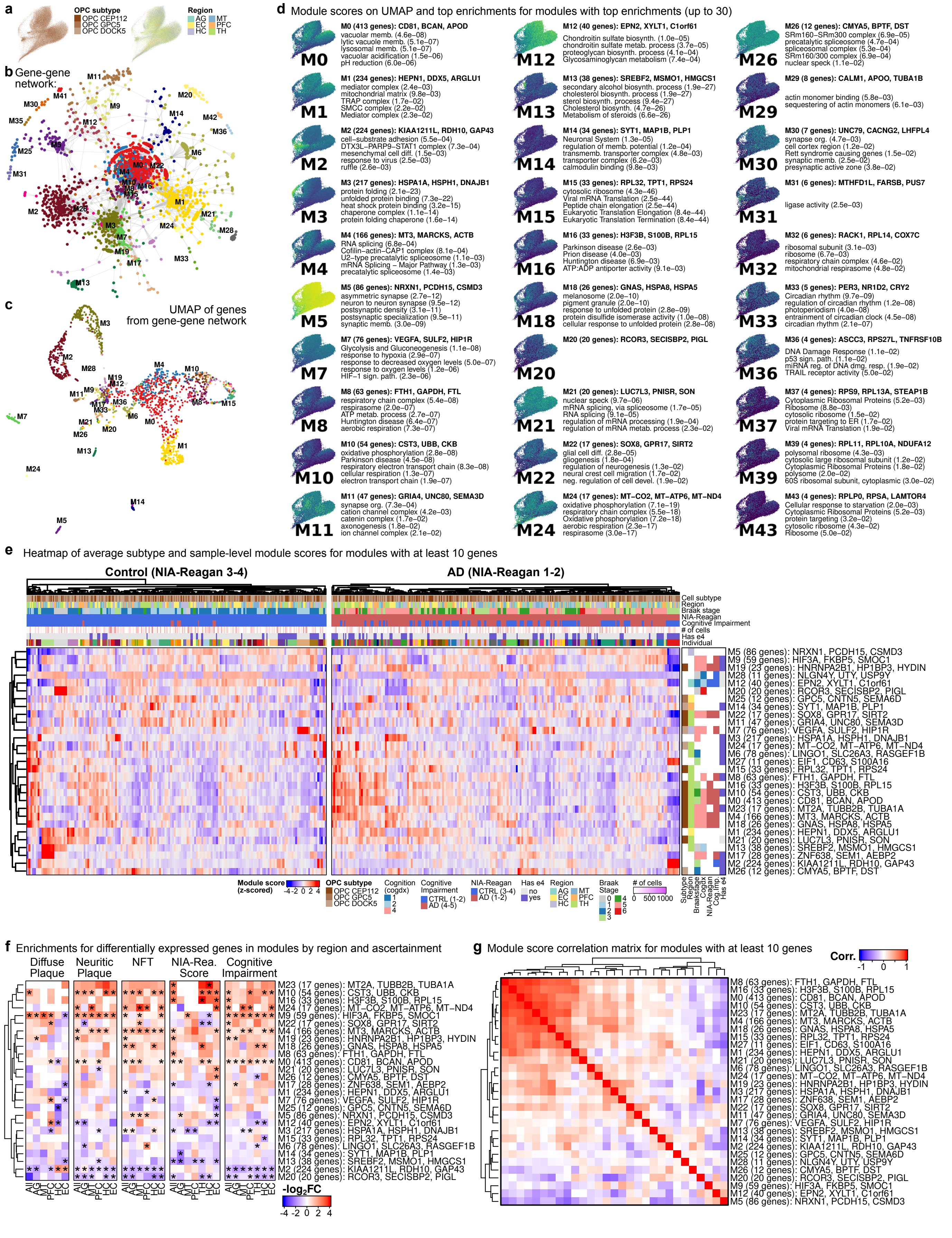

Supplementary Figure 33

Supplementary Figure 33. OPC modules. a, UMAP plots of cells used to detect modules in OPCs alone, colored by cell subtype and by region of origin. **b,** Gene-gene network from modules method, where each point is a gene, edges connect pairs of genes with above-threshold correlation, and genes are colored by their leiden cluster (module) in the graph. **c,** UMAP of genes from estimated gene-gene correlation from sample-decorrelated matrix. Each point is a gene, colored by the leiden clusters (modules) as in (**b**). **d**, Modules scores on the cell UMAP from (**a**) and the names and p-values of the top gene set enrichments (term size < 500) for the 30 modules with the top enrichments (or top 30 in order of size). Module scores calculated as the average (normalized, log1p) expression of all of the genes in the module. Modules are sorted by number of genes and labeled with the top three genes that are most correlated to the overall module score. **e,** Heatmap of average subtype and sample-level module scores for modules with at least 10 genes. Module scores are z-scored for the heatmap. Top panel annotates columns, giving the properties of each subtype + sample column. Side panel shows hypergeometric enrichments for top z-scoring samples (z > 1) in each metadata column. **f,** Heatmap of hypergeometric enrichments of up (red) or down (blue) AD DEGs in modules for DEGs computed under different conditions and brain regions. Only modules with significant enrichments are shown and rows are clustered hierarchically by Euclidean distance. **g,** Correlation matrix heatmap for module scores for modules with at least 10 genes, with rows and columns hierarchically clustered by Euclidean distance.

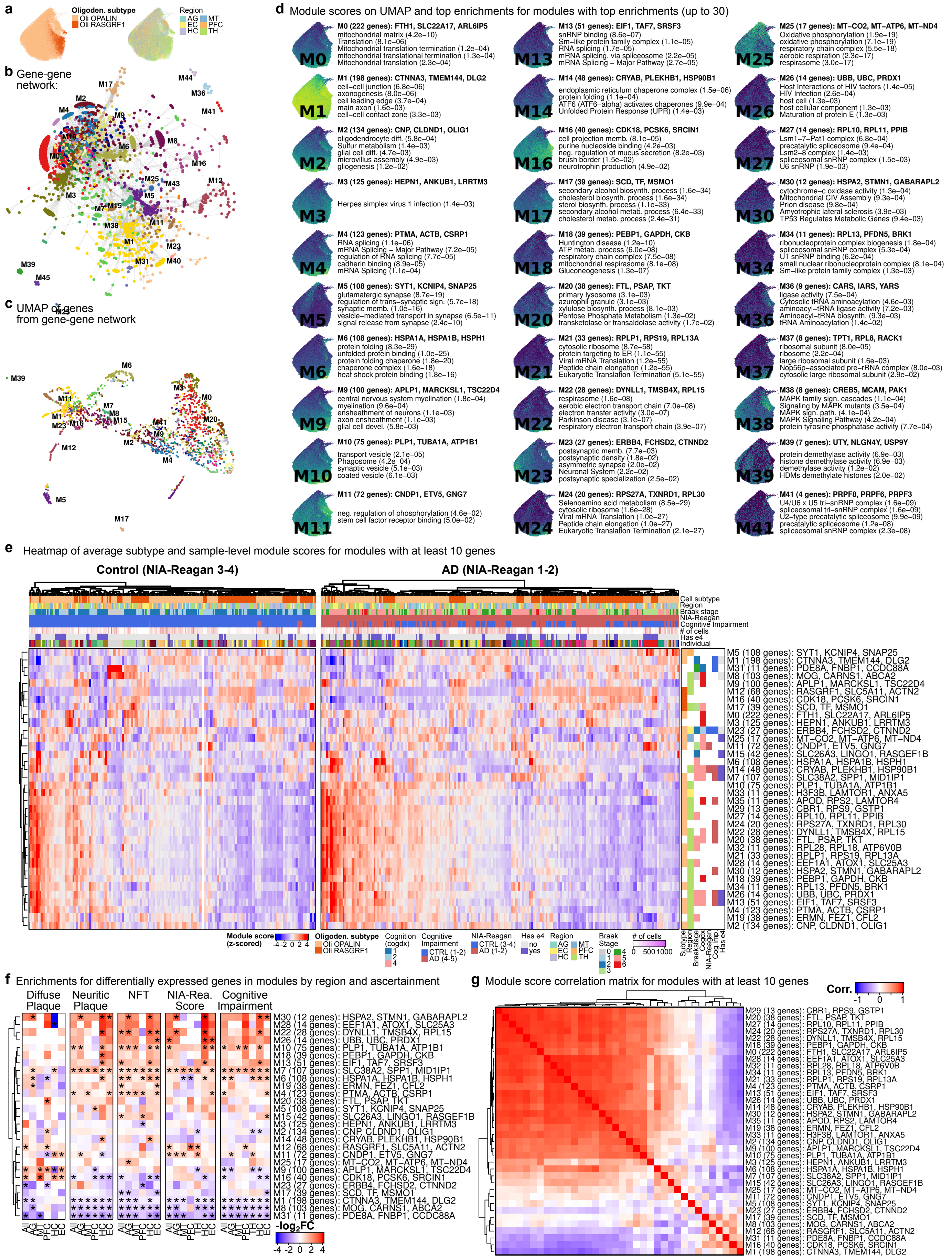

Supplementary Figure 34

Supplementary Figure 34. Oligodendrocyte modules. a, UMAP plots of cells used to detect modules in oligodendrocytes alone, colored by cell subtype and by region of origin. **b,** Gene-gene network from modules method, where each point is a gene, edges connect pairs of genes with above-threshold correlation, and genes are colored by their leiden cluster (module) in the graph. **c,** UMAP of genes from estimated gene-gene correlation from sample-decorrelated matrix. Each point is a gene, colored by the leiden clusters (modules) as in (**b**). **d**, Modules scores on the cell UMAP from (**a**) and the names and p-values of the top gene set enrichments (term size < 500) for the 30 modules with the top enrichments (or top 30 in order of size). Module scores calculated as the average (normalized, log1p) expression of all of the genes in the module. Modules are sorted by number of genes and labeled with the top three genes that are most correlated to the overall module score. **e,** Heatmap of average subtype and sample-level module scores for modules with at least 10 genes. Module scores are z-scored for the heatmap. Top panel annotates columns, giving the properties of each subtype + sample column. Side panel shows hypergeometric enrichments for top z-scoring samples (z > 1) in each metadata column. **f,** Heatmap of hypergeometric enrichments of up (red) or down (blue) AD DEGs in modules for DEGs computed under different conditions and brain regions. Only modules with significant enrichments are shown and rows are clustered hierarchically by Euclidean distance. **g,** Correlation matrix heatmap for module scores for modules with at least 10 genes, with rows and columns hierarchically clustered by Euclidean distance.

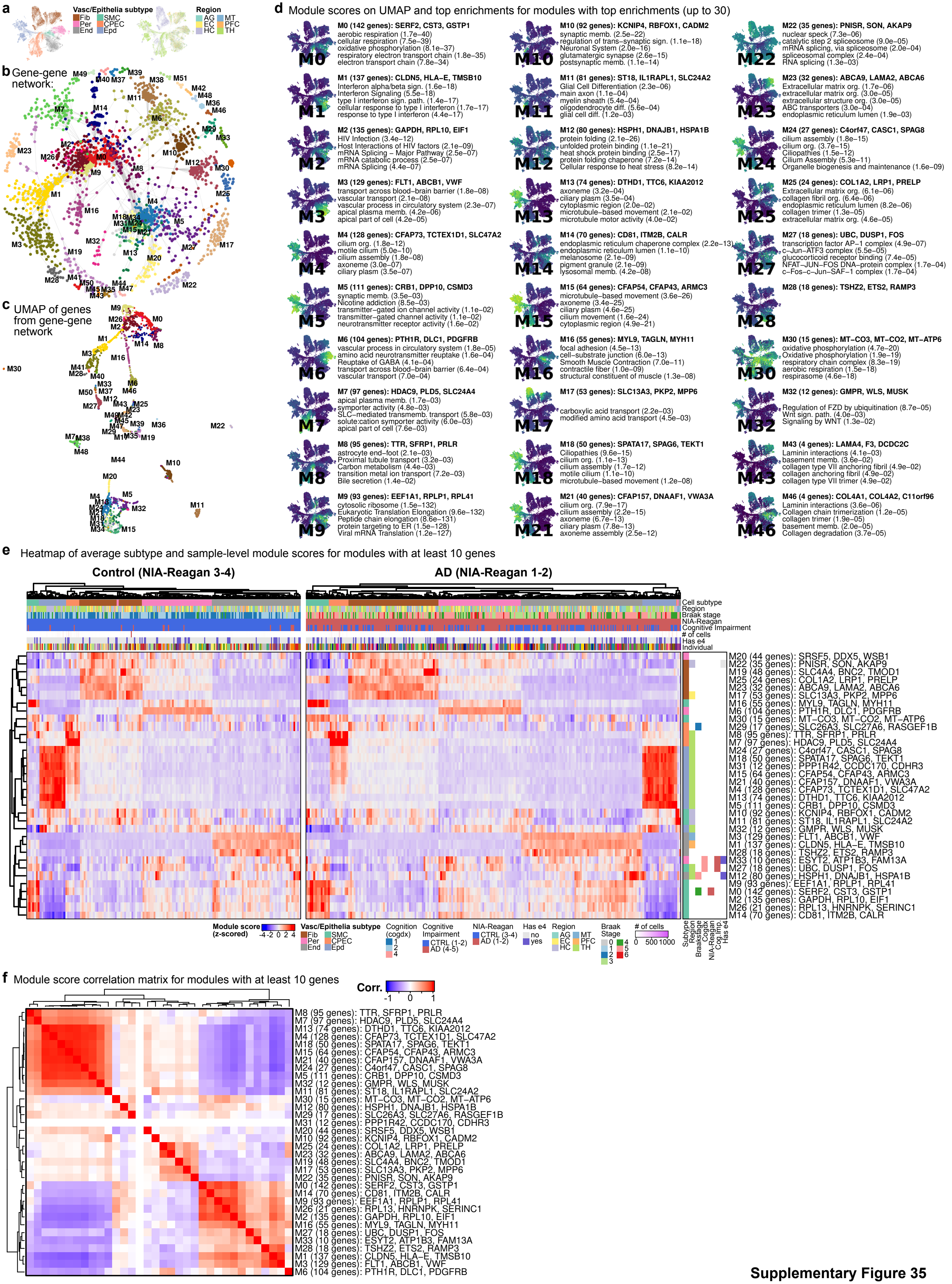

Supplementary Figure 35

Supplementary Figure 35. Vasculature modules. a, UMAP plots of cells used to detect modules in vasculature/epithelia alone, colored by cell subtype and by region of origin. **b,** Gene-gene network from modules method, where each point is a gene, edges connect pairs of genes with above-threshold correlation, and genes are colored by their leiden cluster (module) in the graph. **c,** UMAP of genes from estimated gene-gene correlation from sample-decorrelated matrix. Each point is a gene, colored by the leiden clusters (modules) as in (**b**). **d**, Modules scores on the cell UMAP from (**a**) and the names and p-values of the top gene set enrichments (term size < 500) for the 30 modules with the top enrichments (or top 30 in order of size). Module scores calculated as the average (normalized, log1p) expression of all of the genes in the module. Modules are sorted by number of genes and labeled with the top three genes that are most correlated to the overall module score. **e,** Heatmap of average subtype and sample-level module scores for modules with at least 10 genes. Module scores are z-scored for the heatmap. Top panel annotates columns, giving the properties of each subtype + sample column. Side panel shows hypergeometric enrichments for top z-scoring samples (z > 1) in each metadata column. **f,** Heatmap of hypergeometric enrichments of up (red) or down (blue) AD DEGs in modules for DEGs computed under different conditions and brain regions. Only modules with significant enrichments are shown and rows are clustered hierarchically by Euclidean distance. **g,** Correlation matrix heatmap for module scores for modules with at least 10 genes, with rows and columns hierarchically clustered by Euclidean distance.

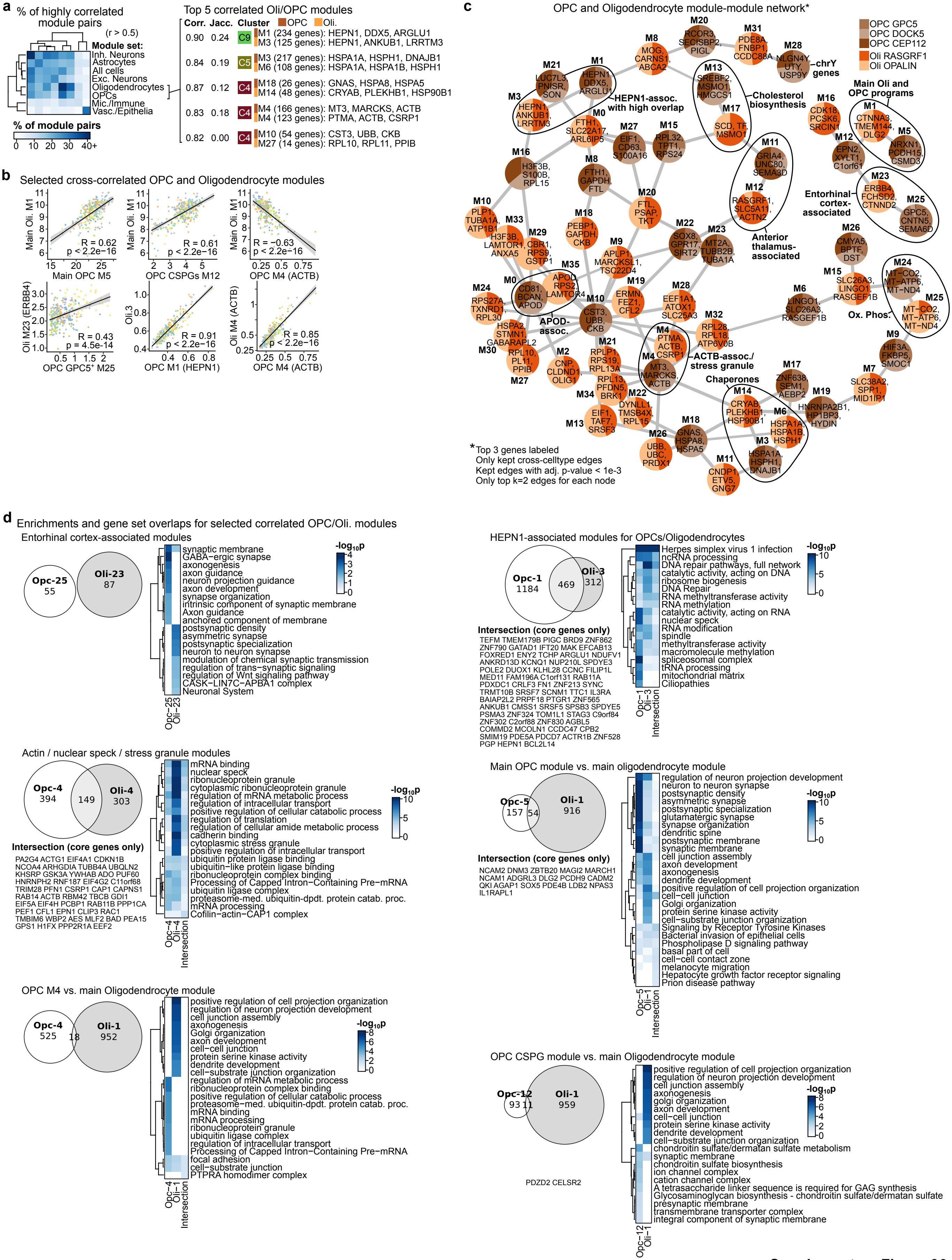

Supplementary Figure 36

Supplementary Figure 36. Cross-correlated pairs of OPC and oligodendrocyte gene expression modules. a, Heatmap showing percent of pairs of modules with high correlation ($r > 0.5$) between each pair of major cell groups (left). Top 5 correlated oligodendrocyte and OPC modules, showing sample-level correlation, jaccard overlap of constituent genes, and top genes for each module (right). **b,** Scatterplots and correlation of scores for selected pairs of OPC and oligodendrocyte modules. Each dot represents scores at the sample-level and is colored by region of origin. Grey area represents the 95% confidence interval around the linear fit. **c,** Module-module network for OPC (brown) and oligodendrocyte (orange) modules from correlation of module pairs at the sample level, keeping only edges between modules detected in different cell types (and where correlation has FDR-adjusted p-value < 0.01). Edges were further pruned to keep the top two edges for each node and the undirected graph was simplified to merge repeated edges. Nodes are colored by module's relative expression in each of the OPC or oligodendrocyte subtypes and groups highlight pairs of modules with similar function or localization. **d,** Enrichments and gene set overlaps for selected correlated OPC and oligodendrocyte modules from (**c**). Gene set overlaps for the full set of genes assigned to each of the modules shown as a scaled venn diagram (euler diagram), with intersecting core genes below. Functional enrichments are shown for each module's genes and their intersection (up to 10 terms per set).

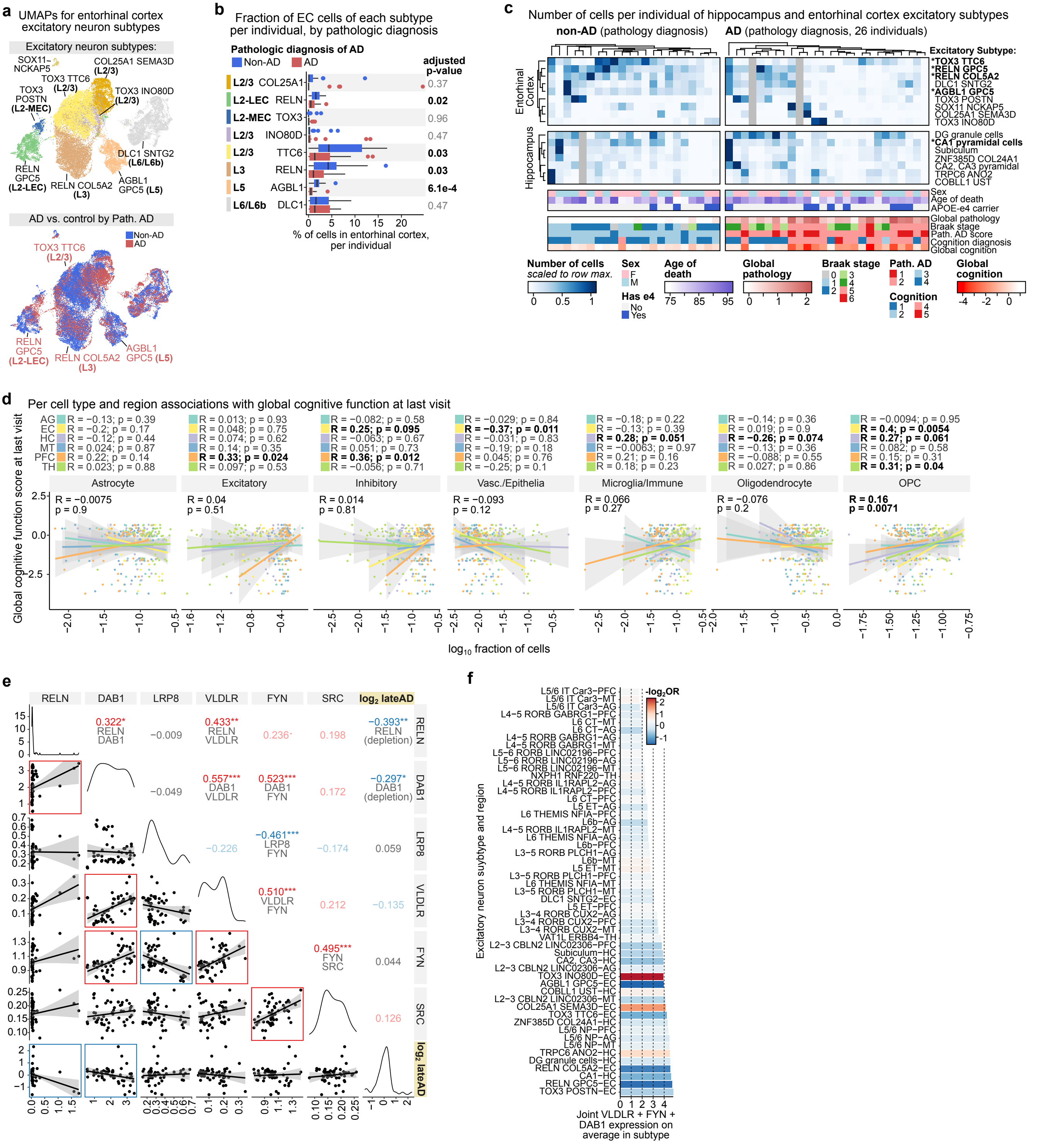

Supplementary Figure 37

Supplementary Figure 37. Neuronal vulnerability, connectivity, and markers. **a,** Entorhinal cortex excitatory subtype UMAP by neuronal subtype and by pathology diagnosis of AD. **b,** Boxplots of fraction of entorhinal cortex excitatory neuron subtypes in each individual, split by AD status, FDR-corrected p-values from one-sided Wilcoxon test. **c,** Heatmap of cell number (per individual, columns) of hippocampus and entorhinal cortex excitatory subtypes (rows). Cells are scaled to the maximum number for each row. Individuals are split by pathology diagnosis of AD, and annotated with covariates (sex, age of death, APOE-OPC-M carrier status) and individual-level AD variables. Heatmap is gray where data is not available (two individuals for the entorhinal cortex, one for the hippocampus). **d,** Scatterplots of major cell type $\log_{10}$ fraction in each of the six regions (colors) against global cognitive function performance at last visit. Lines represent best linear regression fits with 95% CIs, overall correlation and p-value are given in black in the scatter plot and by-region correlations and p-values are given above the plots, with nominal p-value < 0.1 in bold. **e,** Pairwise scatter plots for gene expression at the subtype level for genes in the Reelin signaling pathway, showing strong co-expression between RELN, DAB1, VLDLR, and FYN. **f,** Barplot of joint average expression of VLDLR, FYN, and DAB1 in each neuronal subtype, coloured by its compositional difference ($\log_2$OR) in lateAD.

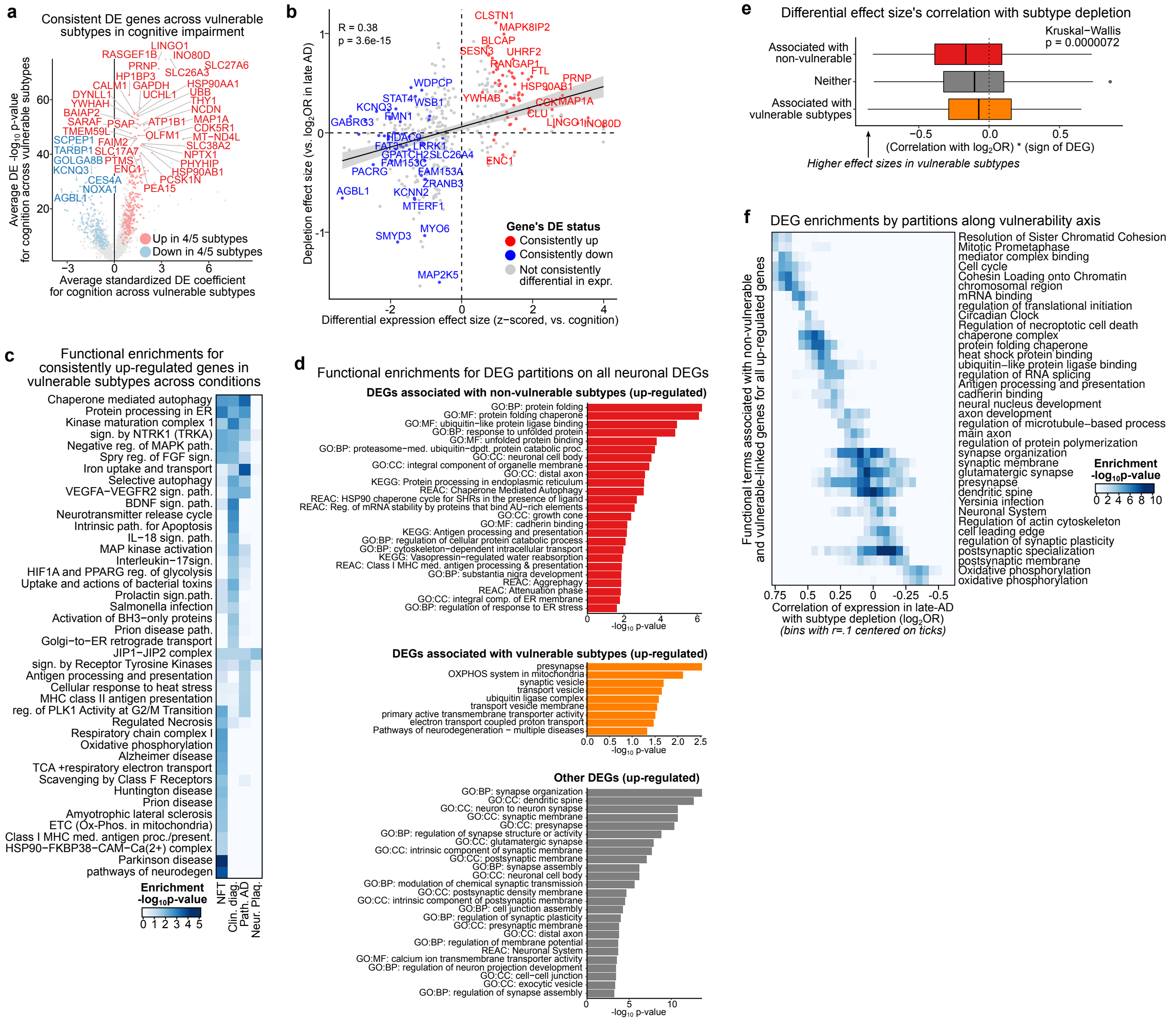

Supplementary Figure 38

Supplementary Figure 38. Neuronal DEGs and DEG partitions. a, Consistent DEGs in AD for AD-vulnerable neuronal subtypes. **b,** Scatterplot of genes' average differential expression effect size across neuronal subtypes versus their effect size in predicting subtype compositional differences (association with subtype vulnerability). Consistently up/down DEGs are coloured in red/blue. **c,** Functional enrichments for consistently up-regulated genes in vulnerable subtypes across conditions. **d,** Functional enrichments for neuronal partitions (top 250 genes by effect size in each set). **e,** Boxplots showing the correlation of genes' differential effect sizes across subtypes with subtype depletion. **f,** Functional enrichments on genes binned across the non-vulnerable to vulnerable spectrum as defined by the correlation of the gene's late-AD expression with the level of subtype depletion in late-AD, across subtypes.

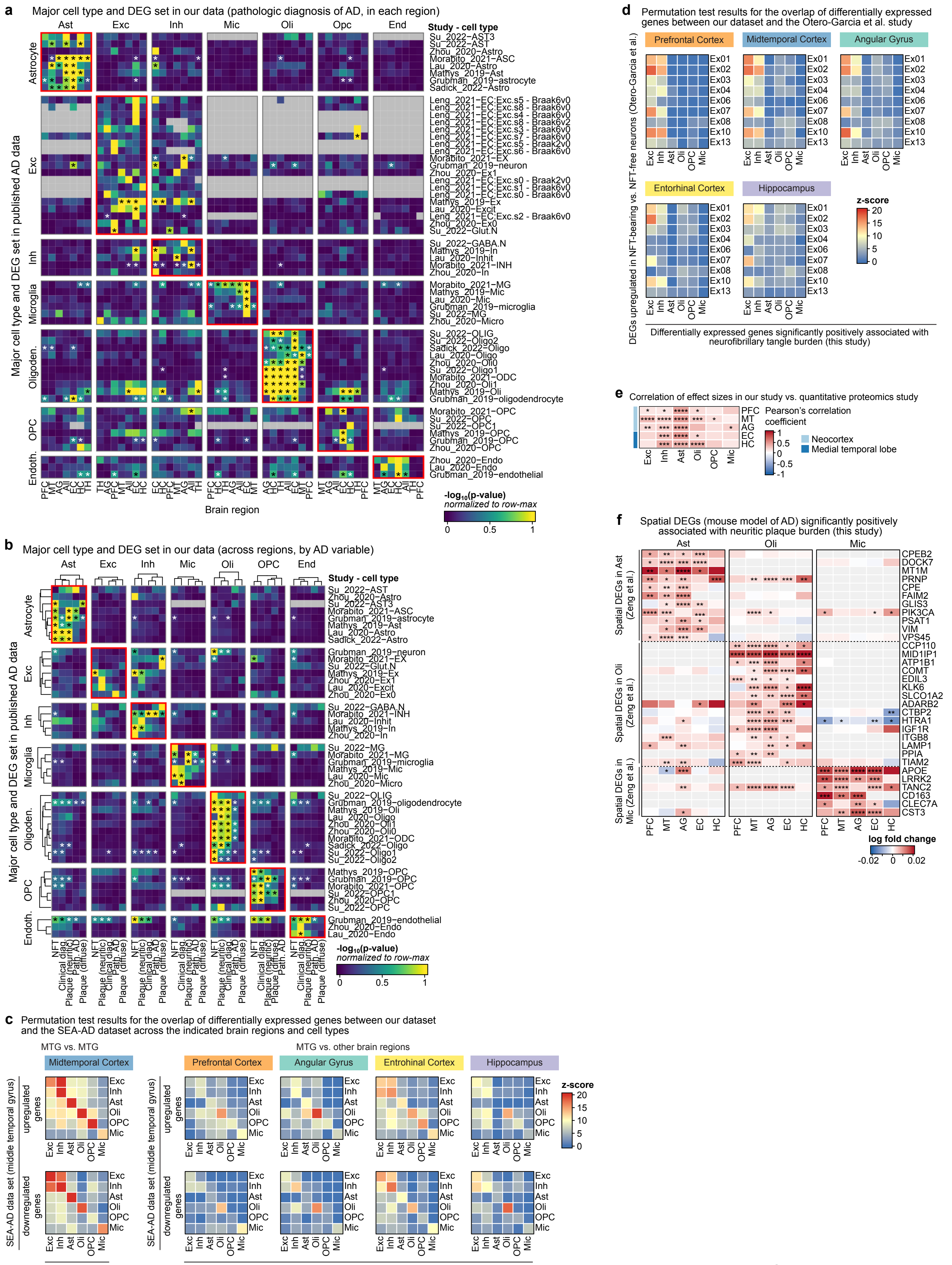

Supplementary Figure 39

Supplementary Figure 39. Regional DEG comparison with outside studies. a-b, Heatmap of -$\log_{10}$ p-values for Pearson correlation between logFC for our differential genes and those for genes in published studies in specific cell types. Comparison is performed for major cell types and endothelial cells for DEG sets computed for pathology diagnosis of AD in each region and over all regions jointly (**a**) as well as for DEGs for five AD ascertainment variables over all regions jointly (**b**). Values are row-normalized to the maximum -$\log_{10}$p-value for a given published DEG set. Cell is starred if p < 1e-3 and correlation > 0.2 (**a**) or > 0.3 (**b**). Cells are gray (not tested) if fewer than 25 genes overlapped in our tested genes with reported DEGs. **c,** Permutation test results for the overlap of differentially expressed genes between our dataset and the SEA-AD dataset across the brain regions and cell types indicated. Heatmaps illustrate the permutation test results for the overlap of DEGs between our dataset (columns) and the SEA-AD dataset (rows). The heatmap displays z-scores, which represent the difference between the observed value of overlap and the mean value of overlap based on the permutation results, divided by the standard deviation of the permutation results. Differentially expressed genes in our dataset were determined based on the neuritic plaque burden and DEGs in the SEA-AD dataset were determined based on the continuous pseudo-progression score of AD severity. Upper row of panels: genes positively associated with AD pathology; Lower row of panels: genes negatively associated with AD pathology. **d,** Permutation test results for the overlap of differentially expressed genes between our dataset and the Otero-Garcia et al. study across the brain regions and cell types indicated (as in **c**). Differentially expressed genes in our dataset were determined based on the neurofibrillary tangle (NFT) burden and DEGs in the Otero-Garcia et al. dataset were determined comparing NFT-bearing and NFT-free neurons (Otero-Garcia et al.). **e,** Correlation between the effect sizes of genes associated with neuritic plaque burden identified in our study and the effect sizes of overlapping differentially expressed proteins observed based on quantitative proteomics data (Johnson et al.). The heatmap displays Pearson's correlation coefficients for the brain regions (rows) and glial cell types (columns) indicated. ****P < 0.0001, ***P < 0.001, **P < 0.01, *P < 0.05 (P values were determined using the cor.test function in R [alternative = "two-sided"], and adjusted for multiple testing using the Benjamini-Hochberg method). **f,** Results of differential gene expression analysis for spatial DEGs identified in a mouse model of Alzheimer's disease (AD) (Zeng et al.). The heatmap displays spatial DEGs significantly positively associated with neuritic plaque burden in at least three brain regions for each glial cell type (astrocytes, oligodendrocytes, and microglia). Effect sizes for genes (rows) in the indicated cell types and brain regions (columns) are shown. Significance levels are indicated as follows: ****p<0.0001, ***p<0.001, **p<0.01, *p< 0.05. P values were determined using Nebula and adjusted for multiple testing with the p.adjust function in R, employing the false discovery rate (FDR) method.

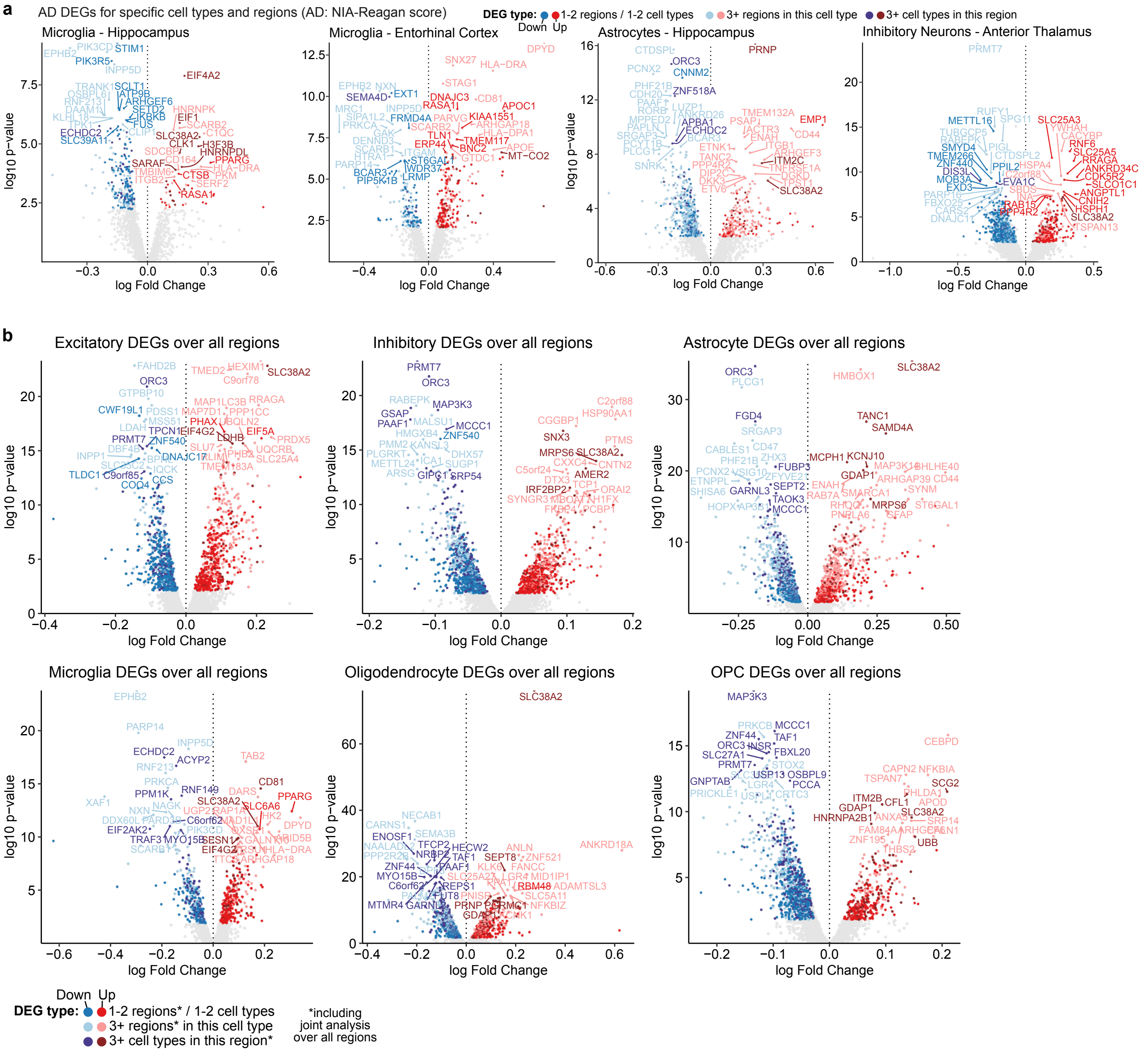

Supplementary Figure 40

Supplementary Figure 40. Regional differential expression changes in AD. a-b, Differential gene volcano plots (log Fold Change against -log10 p-value) for selected cell types and regions (**a**) and for each of the major cell types' DEGs over all regions jointly (**b**). Fold change and p-value for Nebula are plotted. Genes are colored by both their significance and the level of sharing across cell types.

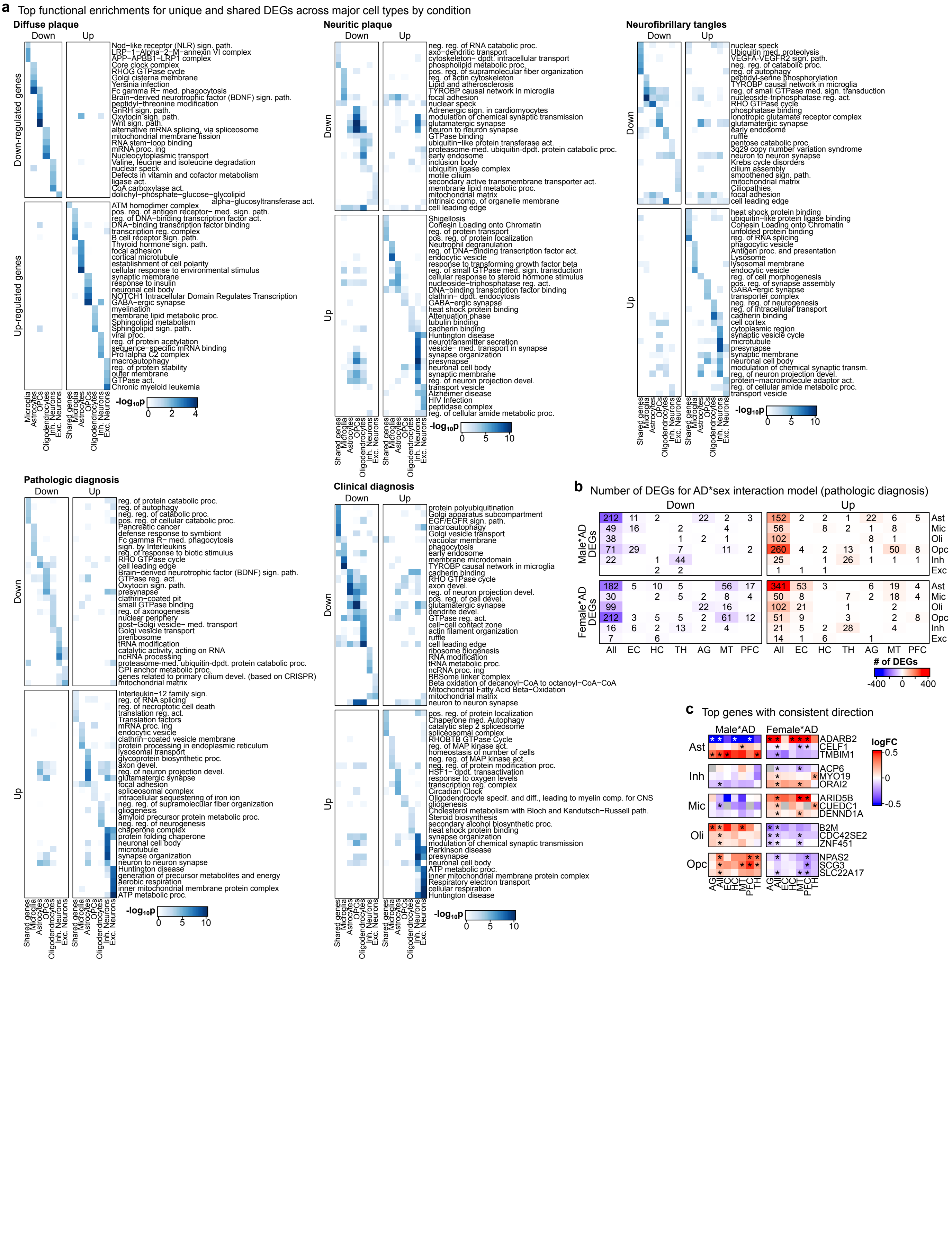

Supplementary Figure 41

Supplementary Figure 41. Cross-region expression differences in AD in major cell types. a, Top functional enrichments for unique DEGs and DEGs shared across cell types (≥3 cell types) across major cell types by condition (up to the top 5 terms with fewer than 500 genes shown per gene set). **b,** Number of DEGs for the interaction between pathologic diagnosis of AD and sex in each major cell type, both across all regions and in each region separately. **c**, Heatmap of male or female-specific AD log fold changes for top example genes with consistent directions across regions in each major cell type.

a R-squared values for predictions of AD status (cognitive level or regional pathology variables) from gene expression module scores at the sample level

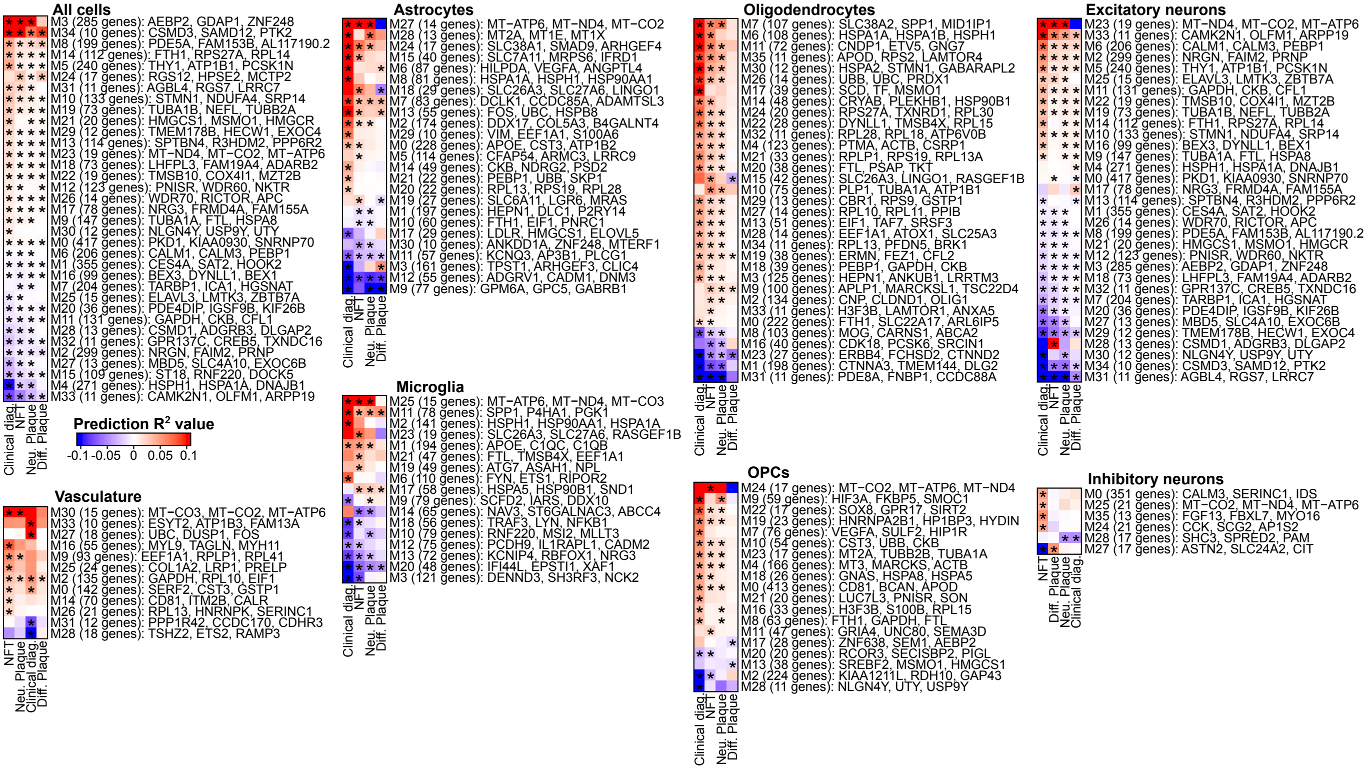

b Top 10 functional enrichments for all genes associated with each glial metabolism-associated module
Up to top 5 differentially expressed genes shown per term (ordered by DEG p-values)

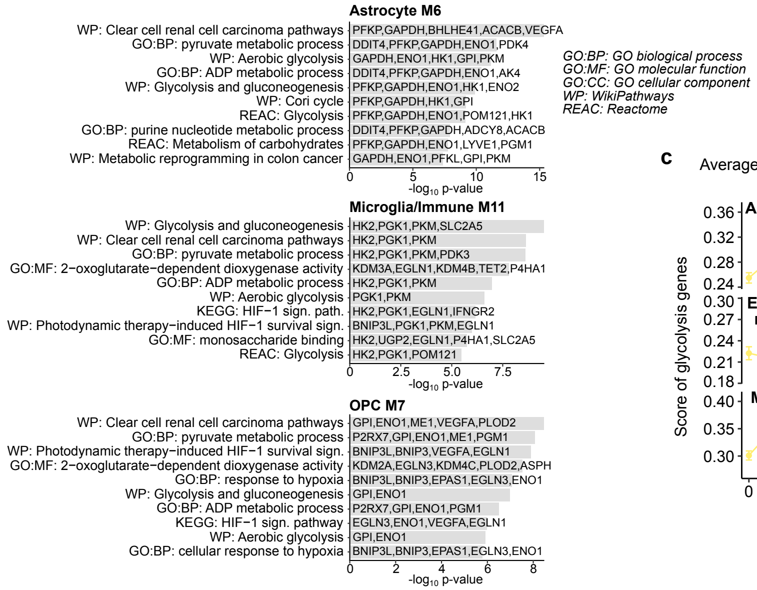

c Average glycolysis pathway expression across regions and ABC score

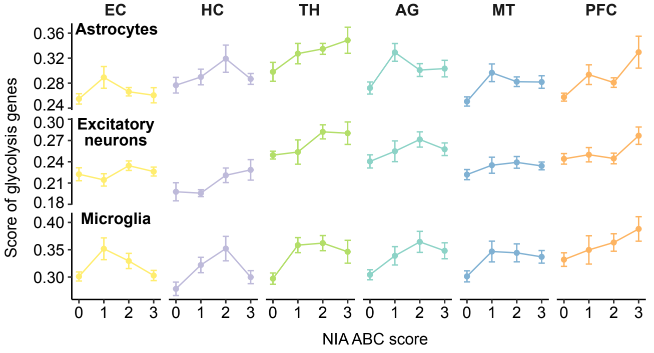

Supplementary Figure 42

Supplementary Figure 42. Module scores versus Alzheimer's disease status. a, Heatmap of r-squared values for predictions of AD status (cognitive level or regional pathology variables) from gene expression module scores at the sample level. Only modules with significant predictions are shown. rows are clustered hierarchically by Euclidean distance. **b,** Top 10 functional enrichments for all genes associated with each glial glycolysis-associated module. Only terms with fewer than 500 genes shown. Terms were pruned by removing terms that had Jaccard similarity > 0.75 with another more significantly enriched term. **c,** Glycolysis pathway expression (pseudo bulk at the sample-level) across ABC score by regions for astrocytes, excitatory neurons, and microglia. Error bars estimated as 1.96*sd by 100 bootstraps, sampling 36 individuals without replacement at a time.

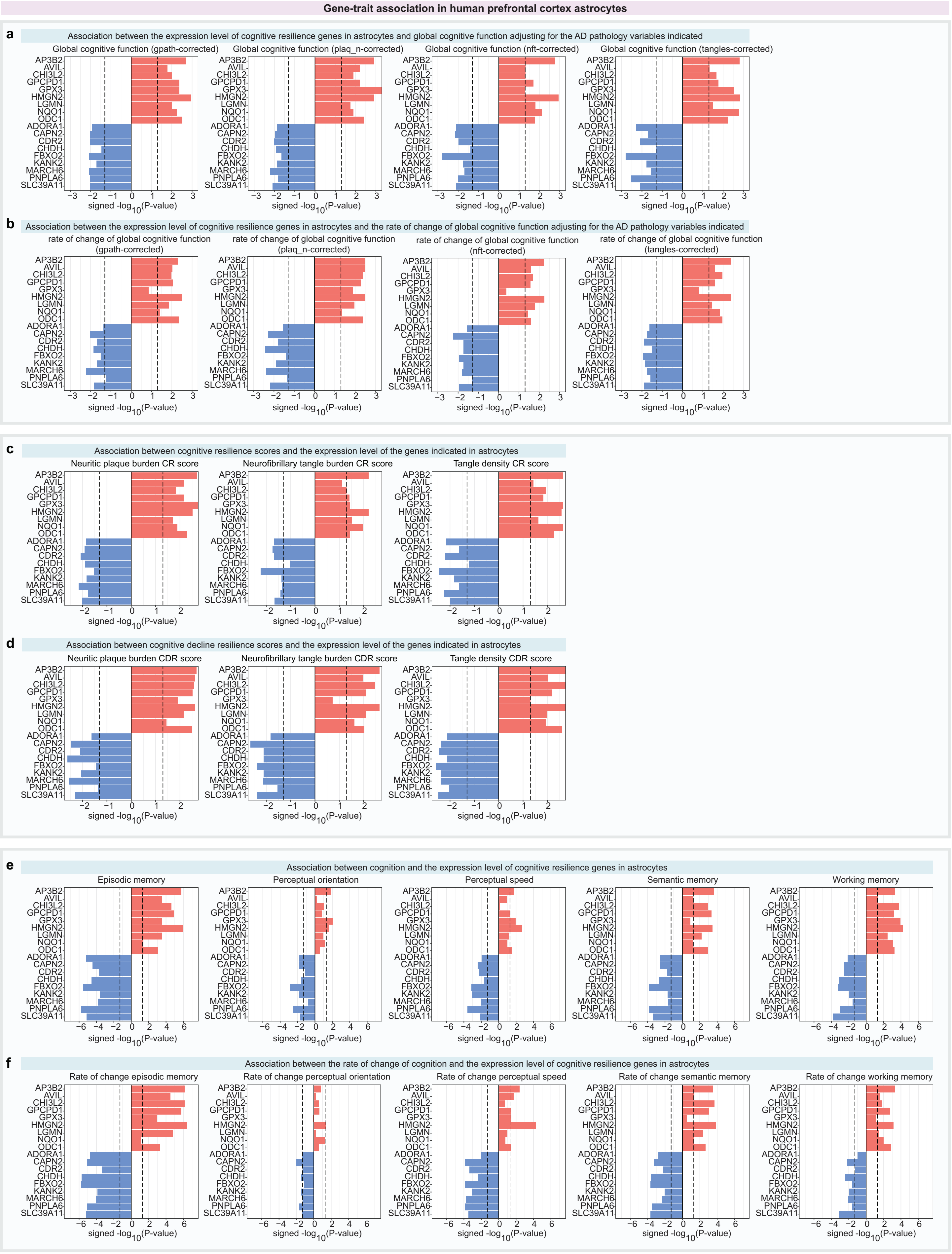

Supplementary Figure 43

Supplementary Figure 43. Gene expression differences in prefrontal cortex astrocytes associated with cognition and cognitive resilience to AD pathology. a-b, Association between the expression level of cognitive resilience genes in prefrontal cortex astrocytes and (**a**) global cognitive function or (**b**) the rate of change of global cognitive function adjusting for the AD pathology variables indicated. The bar plots show association scores (signed $-\log_{10}$ FDR-adjusted P value, where the sign was determined by the direction (positive or negative) of the association). **c-d,** Association between (**c**) cognitive resilience scores or (**d**) cognitive decline resilience scores and the expression level of the genes indicated in prefrontal cortex astrocytes (barplots as in panel **a**). **e-f,** Association between (**e**) cognition or (**f**) the rate of change of cognition and the expression level of cognitive resilience genes in prefrontal cortex astrocytes (barplots as in panel **a**). (**a-f**) Significance testing was conducted using quasi-likelihood F-tests via the glmQLFTest function, as implemented in the R package muscat. P values were adjusted for multiple testing by using the p.adjust function with the BH method as implemented in muscat.

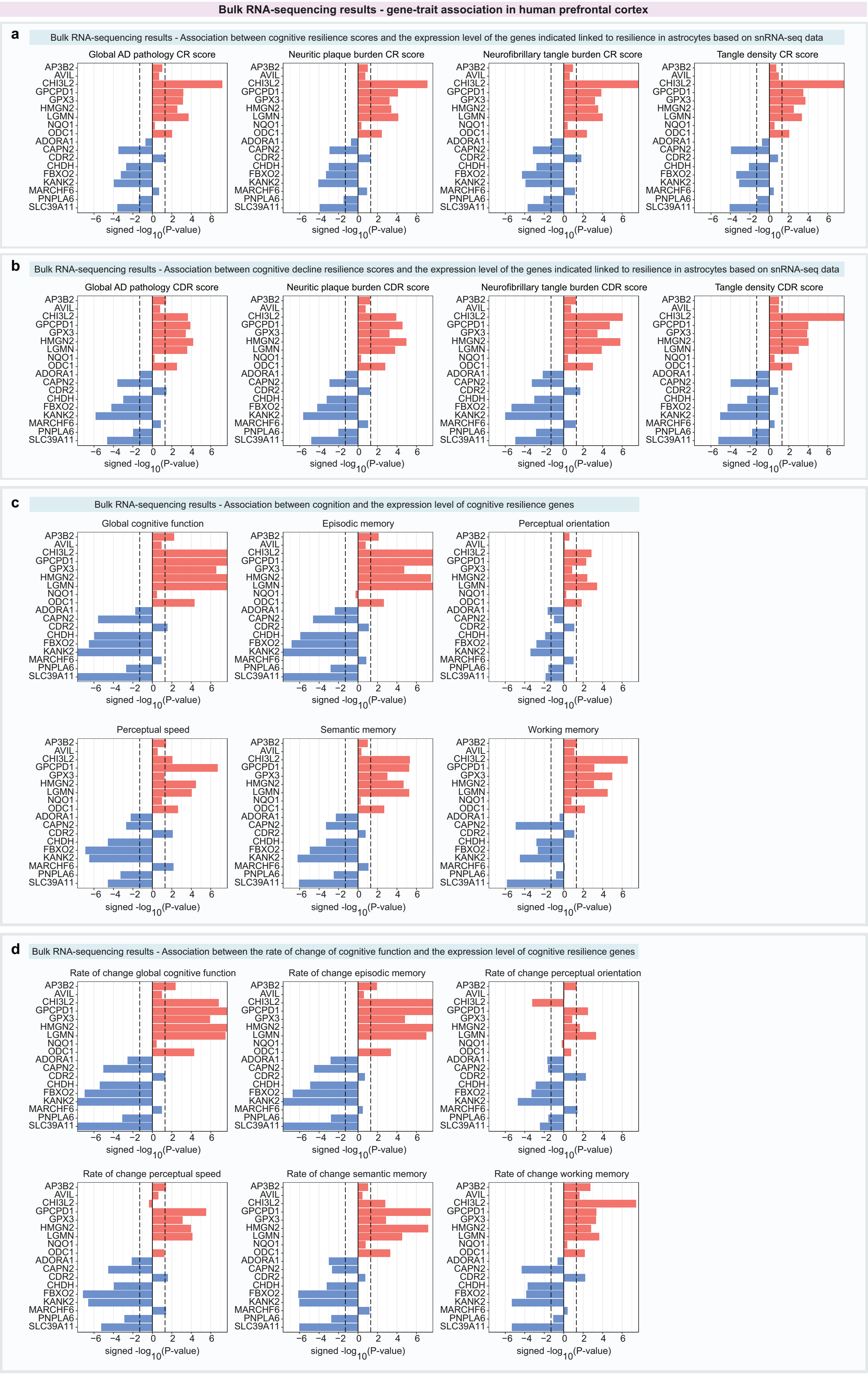

Supplementary Figure 44

Supplementary Figure 44. Confirmation of gene expression differences associated with cognition and cognitive resilience using bulk RNA-sequencing data. a-d, Differential expression analysis results based on bulk RNA sequencing (RNA-seq) data from the dorsolateral prefrontal cortex (DLPFC) of 638 ROSMAP study participants. Association between (**a**) cognitive resilience scores or (**b**) cognitive decline resilience scores, (**c**) cognition, or (**d**) rate of change of cognitive function and expression levels of genes linked to cognitive resilience in astrocytes based on snRNA-seq data. The bar plots show association scores (signed negative log10 FDR-adjusted P value, where the sign was determined by the direction (positive or negative) of the association). (**a-d**) P values were derived using the Wald test as implemented in DESeq2, with adjustments for multiple testing using the Benjamini and Hochberg method.

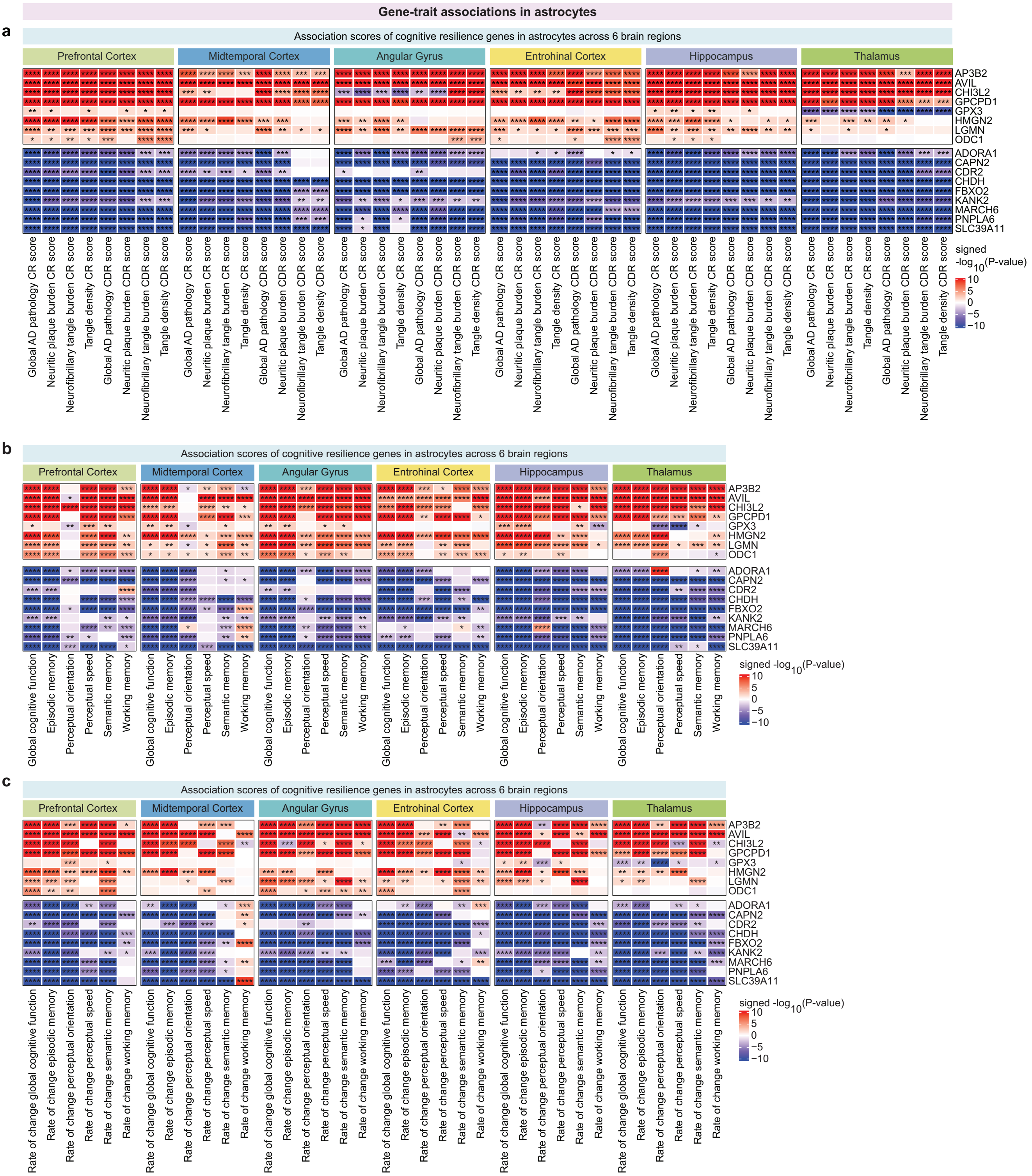

Supplementary Figure 45

Supplementary Figure 45. Gene expression differences in astrocytes associated with cognition, the rate of change of cognition, and cognitive resilience. a-c, Gene-trait association in astrocytes across 6 human brain regions. Association between the expression level of cognitive resilience genes (rows) in astrocytes and measures of (**a**) cognitive resilience, (**b**) cognition, or (**c**) the rate of change of cognition. The heatmap shows association scores (signed negative log10 FDR-adjusted P value, where the sign was determined by the direction (positive or negative) of the association). ****$P < 0.0001$, ***$P < 0.001$, **$P < 0.01$, *$P < 0.05$ (FDR-adjusted likelihood ratio test P value determined by MAST).
