# Supplementary material for: Single-cell multiregion dissection of Alzheimer’s disease
Source: Nature. 2024 Jul 24;632(8026):858–68. doi: 10.1038/s41586-024-07606-7 (PMC11338834; doi:10.1038/s41586-024-07606-7)
Supplement: Supplementary file 2 — Reporting Summary [file 41586_2024_7606_MOESM2_ESM.pdf]

nature portfolio

Corresponding author(s): Li-Huei Tsai and Manolis Kellis

Last updated by author(s): Apr 19, 2024

Reporting Summary

Nature Portfolio wishes to improve the reproducibility of the work that we publish. This form provides structure for consistency and transparency in reporting. For further information on Nature Portfolio policies, see our Editorial Policies and the Editorial Policy Checklist.

Statistics

For all statistical analyses, confirm that the following items are present in the figure legend, table legend, main text, or Methods section.

n/a	Confirmed	
☐	☒	The exact sample size (*n*) for each experimental group/condition, given as a discrete number and unit of measurement
☒	☐	A statement on whether measurements were taken from distinct samples or whether the same sample was measured repeatedly
☐	☒	The statistical test(s) used AND whether they are one- or two-sided<br>*Only common tests should be described solely by name; describe more complex techniques in the Methods section.*
☐	☒	A description of all covariates tested
☐	☒	A description of any assumptions or corrections, such as tests of normality and adjustment for multiple comparisons
☐	☒	A full description of the statistical parameters including central tendency (e.g. means) or other basic estimates (e.g. regression coefficient) AND variation (e.g. standard deviation) or associated estimates of uncertainty (e.g. confidence intervals)
☐	☒	For null hypothesis testing, the test statistic (e.g. *F*, *t*, *r*) with confidence intervals, effect sizes, degrees of freedom and *P* value noted<br>*Give P values as exact values whenever suitable.*
☒	☐	For Bayesian analysis, information on the choice of priors and Markov chain Monte Carlo settings
☒	☐	For hierarchical and complex designs, identification of the appropriate level for tests and full reporting of outcomes
☐	☒	Estimates of effect sizes (e.g. Cohen's *d*, Pearson's *r*), indicating how they were calculated

Our web collection on statistics for biologists contains articles on many of the points above.

Software and code

Policy information about availability of computer code

Data collection: no software was used

Data analysis:
Gene counts were obtained by aligning reads to the GRCh38 genome using Cell Ranger software (v.3.0.2) (l0x Genomics).
We used SCAN PY (vl.6) to process and cluster the expression profiles and infer cell identities.
We called doublets using DoubletFinder (v2.0).
The R package Seurat (v.3.2) was used for UMAP visualizations and to determine marker genes.
Prism 9 software was used for histogram visualizations and for statistical analyses comparing the number of genes and transcripts detected per cell.
The Differential Search tool of the Allen Brain Atlas data portal (v.7) (https://human.brain-map.org/microarray/search) was used to compare microarray data from different human brain regions.
The Consensus Non-negative Matrix factorization (cNMF) (vl.3) package implemented in python was used for the NMF analysis.
The gene regulatory network analysis was performed using pySCENIC (v0.10.4).
MAST (vl.16.0) and Nebula (vl.1.7) were used for the differential expression analyses.
The gprofiler2 package in R DEG was used for DEG enrichments.
Cell-cell communication events were predicted using the LIgand-receptor ANalysis frAmework (LIANA) (v0.1.12).
The differential expression analysis comparing vulnerable to non-vulnerable inhibitory neuron subtypes was performed with the R package dreamlet (v0.99.6).
Genes associated with cognitive resilience were identified using the R package muscat (v1.12.1).
Bulk RNA-seq differential expression analysis was performed using DESeq2 (v1.38.3).
Further statistical analyses and visualizations were implemented in R version 4.0.3 {2020-10-10).
Gene expression modules were determined using the scdemon (v0.9.0) framework. The code for the scdemon method for module detection

from single-cell RNA-seq is available at https://github.com/KellisLab/scdemon.
Code for analysis is available at https://github.com/cboix/admultiregion_analysis and Zenodo (11051021, https://doi.org/10.5281/zenodo.11051021).

For manuscripts utilizing custom algorithms or software that are central to the research but not yet described in published literature, software must be made available to editors and reviewers. We strongly encourage code deposition in a community repository (e.g. GitHub). See the Nature Portfolio guidelines for submitting code & software for further information.

Data

Policy information about availability of data

All manuscripts must include a data availability statement. This statement should provide the following information, where applicable:

- Accession codes, unique identifiers, or web links for publicly available datasets
- A description of any restrictions on data availability
- For clinical datasets or third party data, please ensure that the statement adheres to our policy

Single-nucleus RNA-seq profiling data is available from Synapse (www.synapse.org) in coordination with the ROSMAP project. Data is accessible at https://www.synapse.org/#!Synapse:syn52293442 (as part of The MIT ROSMAP Single-Nucleus Multiomics Study https://www.synapse.org/#!Synapse:syn52293417). The data is available under controlled use conditions set by human privacy regulations. To access the data, a data use agreement is needed. This registration is in place solely to ensure anonymity of the ROSMAP study participants. A data use agreement can be agreed with either Rush University Medical Center (RUMC) or with SAGE, who maintains Synapse, and can be downloaded from their websites (https://www.radc.rush.edu/; https://adknowledgeportal.synapse.org/). Additional processed data as well as integrative visualization and exploration of the atlas are available through http://compbio.mit.edu/ad_multiregion/ and https://ad-multi-region.cells.ucsc.edu/.

We also downloaded the following public single-cell gene expression datasets: Human Multiple Cortical Areas SMART-seq (https://portal.brain-map.org/atlases-and-data/rnaseq/human-multiple-cortical-areas-smart-seq), human DLPFC (https://www.synapse.org/#!Synapse:syn51123521), SEA-AD MTG (https://sea-ad-single-cell-profiling.s3.amazonaws.com/index.html#MTG/RNAseq/), SEA-AD DLPFC (https://sea-ad-single-cell-profiling.s3.amazonaws.com/index.html#DLPFC/RNAseq/), human dLGN (https://portal.brain-map.org/atlases-and-data/rnaseq/comparative-lgn), multiple human brain regions (https://console.cloud.google.com/storage/browser/linnarsson-lab-human;tab=objects?authuser=0&prefix=&forceOnObjectsSortingFiltering=false), multiple cortical areas and the hippocampal formation of the mouse brain (https://portal.brain-map.org/atlases-and-data/rnaseq/mouse-whole-cortex-and-hippocampus-10x), nine regions in the adult mouse brain (http://dropviz.org/), and Mouse Brain Atlas (http://mousebrain.org/).

Research involving human participants, their data, or biological material

Policy information about studies with human participants or human data. See also policy information about sex, gender (identity/presentation), and sexual orientation and race, ethnicity and racism.

Reporting on sex and gender	We selected 48 individuals from the Religious Orders Study and Rush Memory and Aging Project (ROSMAP), both ongoing longitudinal clinical-pathologic cohort studies of aging and dementia, in which all participants are brain donors. Individuals were balanced between sexes (male:female ratios 13:13 in AD, 11:11 in NoAD) (sex was determined based on self-reporting).
Reporting on race, ethnicity, or other socially relevant groupings	No socially constructed or socially relevant categorization variables were used in this study.
Population characteristics	We selected 48 individuals from the Religious Orders Study and Rush Memory and Aging Project (ROSMAP), both ongoing longitudinal clinical-pathologic cohort studies of aging and dementia, in which all participants are brain donors. For the purpose of this study, individuals were selected based on the modified NIA-Reagan diagnosis of Alzheimer's disease and the Braak stage score (Braak stage 0,1,2, n=20; Braak stage 3,4, n=14; Braak stage 5,6, n=14), with 26 individuals having a positive pathologic diagnosis of AD and 22 individuals having a negative pathologic diagnosis of AD. Individuals were balanced between sexes (male:female ratios 13:13 in AD, 11:11 in NoAD), matched for age (medians 86.6 years (AD) and 86.0 years (NoAD)), and postmortem interval (medians 5.9 hours (AD) and 6.3 hours (NoAD)).
Recruitment	No donors were recruited, the tissue has been obtained from participants in the Religious Order Study.
Ethics oversight	The Religious Orders Study and Rush Memory and Aging Project were approved by an IRB of Rush University Medical Center.

Note that full information on the approval of the study protocol must also be provided in the manuscript.

Field-specific reporting

Please select the one below that is the best fit for your research. If you are not sure, read the appropriate sections before making your selection.

☒ Life sciences ☐ Behavioural & social sciences ☐ Ecological, evolutionary & environmental sciences

For a reference copy of the document with all sections, see nature.com/documents/nr-reporting-summary-flat.pdf

Life sciences study design

All studies must disclose on these points even when the disclosure is negative.

Sample size	No explicit calculations were performed to determine sample size. Rather, we aimed to analyze brain tissue from an equal number of men and women and at least 11 individuals per group. Therefore we analyzed brain tissue from 26 individuals having a positive pathologic diagnosis of AD and 22 individuals having a negative pathologic diagnosis of AD. The sample size of 48 was chosen based on findings from our previous study, which also included the same number of participants. This previous study demonstrated that a sample size of 48 is sufficient to detect significant differences between individuals diagnosed with and without Alzheimer's Disease.
Data exclusions	Low quality snRNA-seq libraries were excluded and the exclusion criteria are described in the manuscript as follows. We kept only protein coding genes and filtered out cells with over 20% mitochondrial or 5% ribosomal RNA, leaving 1.47M cells over 48 individuals and 283 samples across all regions. We separately called doublets using DoubletFinder and flagged and removed clusters with strong doublet profiles and clusters showing strong individual-specific batch effects, leaving a final dataset of 1.35M cells.
Replication	Verification of the single-nucleus RNA-seq data was performed through validation using RNA in situ hybridization on post-mortem brain tissue. These experiments validated the findings derived from snRNA-seq. The snRNA-seq experiment was performed once. The RNA in situ hybridization (RNAscope) experiments shown in Figure 1i, Figure 2d, Figure 3g, Figure 4o-p, and Figure 5j-l were each performed once. Similarly, the IHC experiments shown in Figure 3h-i were each performed once.
Randomization	The study participants were allocated into groups based on Braak stage.
Blinding	Investigators were not blinded to group allocation. The outcome measures used in our snRNA-seq analysis are objective, relying on standardized computational methods, which reduces the potential for bias that blinding seeks to mitigate.

Reporting for specific materials, systems and methods

We require information from authors about some types of materials, experimental systems and methods used in many studies. Here, indicate whether each material, system or method listed is relevant to your study. If you are not sure if a list item applies to your research, read the appropriate section before selecting a response.

Materials & experimental systems

n/a	Involved in the study	
☐	☒	Antibodies
☒	☐	Eukaryotic cell lines
☒	☐	Palaeontology and archaeology
☒	☐	Animals and other organisms
☒	☐	Clinical data
☒	☐	Dual use research of concern
☒	☐	Plants

Methods

n/a	Involved in the study	
☒	☐	ChIP-seq
☒	☐	Flow cytometry
☒	☐	MRI-based neuroimaging

Antibodies

Antibodies used

• Anti-reelin
o Host: monoclonal mouse
o Catalogue name: Anti-Reelin Antibody, a.a. 164-496 mreelin, clone G10
o Catalogue number: Millipore Sigma MAB5364
• Anti-NeuN
o Host: polyclonal serum from guinea pig
o Catalogue name: NeuN antibody
o Catalogue number: Synaptic System 266004
• Anti-Amyloid ß
o Host: Monoclonal rabbit
o Catalogue name: β-Amyloid (D54D2) XP® Rabbit mAb
o Catalogue number: 8243S
• Anti-Phospho-tau
o Host: Polyclonal rabbit
o Catalogue name: Phospho-Tau (Ser396)
o Catalogue number: 44-752G

Validation

• Anti-reelin:
Millipore Sigma application statement: Detect Reelin using this Anti-Reelin Antibody, a.a. 164-496 mreelin, clone G10 validated for use in IH & WB.
• Anti-NeuN
Silencing CA1 pyramidal cells output reveals the role of feedback inhibition in hippocampal oscillations.
Adaikkan C, Joseph J, Foustoukos G, Wang J, Polygalov D, Boehringer R, Middleton SJ, Huang AJY, Tsai LH, McHugh TJ

Nature communications (2024) 151: 2190. 266 004 IHC; tested species: mouse
• Anti-Amyloid ß
Cell Signaling Technology Specificity/Sensitivity statement: β-Amyloid (D54D2) XP® Rabbit mAb recognizes endogenous levels of total β-amyloid peptide (Aβ). The antibody detects several isoforms of Aβ, such as Aβ-37, Aβ-38, Aβ-39, Aβ-40, and Aβ-42. This product detects transgenically expressed human APP in mouse models.
• Anti-Phospho-tau
Invitrogen Advanced Verification statement: This Antibody was verified by Cell treatment to ensure that the antibody binds to the antigen stated.

Plants

Seed stocks

Report on the source of all seed stocks or other plant material used. If applicable, state the seed stock centre and catalogue number. If plant specimens were collected from the field, describe the collection location, date and sampling procedures.

Novel plant genotypes

Describe the methods by which all novel plant genotypes were produced. This includes those generated by transgenic approaches, gene editing, chemical/radiation-based mutagenesis and hybridization. For transgenic lines, describe the transformation method, the number of independent lines analyzed and the generation upon which experiments were performed. For gene-edited lines, describe the editor used, the endogenous sequence targeted for editing, the targeting guide RNA sequence (if applicable) and how the editor was applied.

Authentication

Describe any authentication procedures for each seed stock used or novel genotype generated. Describe any experiments used to assess the effect of a mutation and, where applicable, how potential secondary effects (e.g. second site T-DNA insertions, mosiacism, off-target gene editing) were examined.
