# Supplementary material for: Single-cell multiregion dissection of Alzheimer’s disease
Source: Nature. 2024 Jul 24;632(8026):858–68. doi: 10.1038/s41586-024-07606-7 (PMC11338834; doi:10.1038/s41586-024-07606-7)
Supplement: Supplementary file 3 — Supplementary Tables 1–10 and a Supplementary Table guide. [file 41586_2024_7606_MOESM3_ESM.zip › Supp_table_guide.pdf]

Supplementary Tables 1-10

Supplementary Table 1. Sample metadata
Supplementary Table 2. Gene expression marker lists. Marker genes were determined using Seurat's FindMarkers function, applying the Wilcoxon rank-sum test, with adjustment for multiple comparisons via the Bonferroni method.
Supplementary Table 3. Gene expression programs from cNMF
Supplementary Table 4. Transcriptional regulons from SCENIC
Supplementary Table 5. Modules and module scores for GABAergic, glutamatergic, and projecting neurons
Supplementary Table 6. Gene expression modules
Supplementary Table 7. Gene expression module covariate enrichments
Supplementary Table 8. Genes associated with subtype vulnerability. Results for inhibitory neurons were obtained using the dreamletCompareClusters function as implemented in the R package dreamlet.
Supplementary Table 9. Differentially expressed genes for AD pathology and AD diagnosis
Supplementary Table 10. Pathology-biased differentially expressed genes, for neuritic plaque or NFT burden
